# Analysis of financial convergence between the BRICS and OECD countries

**DOI:** 10.1371/journal.pone.0310950

**Published:** 2025-02-03

**Authors:** Nasim Iranmanesh

**Affiliations:** MA in Economics, Shahid Bahonar University of Kerman, Kerman, Iran; University Putra Malaysia: Universiti Putra Malaysia, MALAYSIA

## Abstract

Financial convergence is a process for establishing a relationship among the financial markets of different countries; as a result of such process the rates of similar financial assets in different markets and countries become very close to each other. Some factors might create financial convergence. of which the trade and international capital flows among countries, the presence of banks and other financial institutions in the international arena, the availability of clear and accurate information of markets and financial organizations, and the existence of similar infrastructure and their characteristics from economic, legal and cultural perspectives can be mentioned. The issue of financial convergence with the aim of achieving global financial markets and taking advantage of its capabilities and characteristics would be very important for all countries, especially the emerging countries. This study examines financial convergence in the money and capital markets of the Organization for Economic Co-operation and Development (OECD) and BRICS countries in the period of 2007–2020. The study utilizes the Panel Convergence Methodology and Cluster Analysis (Philips and Sul methodology) along with a clustering algorithm. The findings indicate a lack of overall convergence between OECD and BRICS countries in both financial markets (money and capital) this is due to the lack of similar economic infrastructure, differences in the size of the economy, and variations in trade, financial and monetary freedom indicators, trade relations, and capital transfers. The cluster test in the money market confirms the existence of 3 convergence clubs among the studied countries. The convergence of emerging BRICS countries in the money market, with a large number of OECD developed countries, is a confirmation of the development of their banking sector in the recent years. On the other hand, the capital market survey also shows the presence of 5 convergence clubs between OECD and BRICS countries. Besides, it’s been shown that South Africa, along with Lithuania, Turkey, Slovakia and Latvia, has established a divergent group.

## 1. Introduction

The term globalization was originated in the 1990s and refers to a process of change in which political and economic boundaries are blurred, the connections are extended and cultural interactions among countries are increased. Economic researchers have examined globalization from different dimensions; Krugman defined globalization as the phenomenon of increased integration of global markets. Using the definition of Organisation for Economic Co-operation and Development (OECD), Prof. Cole has defined globalization as an evolving model of cross-border activities of firms, which encompasses international investment, trade, collaboration to innovate, marketing, development and production. Sankliffe-Glenn believes that globalization, in addition to the expansion of capitalism, is recognizable by the interconnectedness of most economies [1]. From the above definitions, it might be concluded that globalization leads to the elimination of regulations and the integration of markets, which leads to convergences as to economic convergence and financial convergence [2]. Economic integration is a type of trade policy that reduces or eliminates discriminatory trade barriers and restrictions between members and the allied countries; whereas financial convergence can be interpreted as the increased financial relations of a country with the outside world. In fact, financial convergence occurs when multiple financial markets merge, leading to a situation where the rates in the financial markets gradually equalize.

Financial convergence can be considered as a result of changes in financial services. Definite property rights, valid accounting standards, and legal mechanisms are the primary factors having role in creating the convergence process [3] and [4]. In addition to what was mentioned above, deregulation, economic integration, harmonization of regulations, corporate governance rules [5], increased trade agreements to enhance trade connections, and greater monetary policy coordination to maintain exchange rate stability and decrease disparities in real interest rates [6], are also factors that contribute to the convergence of financial system characteristics.

Financial convergence allows countries to access more resources at a lower cost, diverse financial instruments, and higher quality financial services that offer better financial intermediation [7]. According to the European Central Bank (2007), increased convergence of financial markets through price stabilization results in a more efficient distribution of risks and also enhances financial stability; besides, financial convergence contributes to the establishment of foreign banks, institutions and financial companies in a country. New established institutions are managed with new technologies that in general lead to development and transformation in the financial sector of the country. These developments are likely to change the restrictions on foreign direct investment, portfolios, borrowing and other financial markets that are more prevalent in developing countries.

Improvement in the level of financial convergence can be associated with higher levels of economic activity; thus, intensifying competition and importing financial services, economic growth might be fulfilled through the enhancement of the domestic financial systems performance. In fact, this relationship remains valid when the level of financial development, human development index, institutional quality, and macroeconomic environment are controlled [8].

Assessment of financial convergence is essential for a wide range of countries, because integration, regardless of any real geographical boundaries, is taking place across the world; therefore, countries aiming to benefit from convergences and integrations, attempt to get closer to other countries through different treaties.

According to the explanations provided, the main purpose of this study is to investigate the financial convergence (in money and capital markets) between BRICS member countries and OECD member countries. On one side, the number of countries which are members of these groups is very high; on the other side, a number of countries have developed economies that have a lot of capital and also are looking for a new environment that might lead them to a more productive investment. This research means examining the financial convergence for a significant number of countries in the world. According to [9], countries with more developed financial systems are more inclined to converge in financial markets. In contrast, emerging countries with significant investment opportunities lacking essential capital and also economies with insufficient liquidity are included in these groups. Based on [10] convergent financial markets enhance the borrowing capacity in emerging economies. Furthermore, being on the way of global markets and taking advantage of technological advancements and capital outflows will pave facilitate the development of these countries. These countries have understood the necessity of integration in the global financial markets for years and they are catching up with their Developed counterparts in recent years and it is noteworthy that during the crisis period of 2008 2009, convergence in their financial markets has reduced less compared to developed countries [11].

The method used in this research that evaluates convergence at different levels using clustering method has been proposed by PS (Phillips and Sul) [12]. In most researches, the convergence hypothesis test has been investigated by the beta, sigma, unit root and co-integration analysis convergence method. [13] states that methods such as beta and sigma are suitable for growth models [14]. Also express that unit root co-integration methods fail when several stable trends are found in the data. But compared to the classical models, the advantage of the cluster approach is that this model examines convergence using a nonlinear model regardless of the preconditions of classical models. This approach includes the experience of countries in transition dynamics, but it does not consider the hypothesis of the development of homogeneous technology, which is a common assumption in most studies. This is very important because the study of any convergence of growth factors using standard static panel test is not valid under technological heterogeneity [12].

Due to the importance of convergence in financial markets, this article addresses the following questions: Is there a general convergence in the money market between OECD and BRICS countries? Is there a general convergence in the capital market between the OECD and BRICS countries? Can any emerging or transition BRICS countries establish an integrated financial market with a group of OECD countries? Are OECD countries converging in each of the financial markets? Are the financial markets of the BRICS countries convergent? In this research, after presenting the introduction, the economic structure of the investigated countries, the concept of financial convergence and the research background have been provided in the second section. The third and fourth sections are dedicated to the research method and model data, respectively. The model is estimated in the fifth section. Discussion and conclusion are discussed in the last two sections.

## 2. Theoretical foundations and research background

In this section, the most important economic indicators of the countries are examined. Then financial convergence is described and the final part also has been assigned to the research background.

### 2.1. Study of economic indicators of BRICS and OECD countries

In the current situation dominating the world, the countries and economic powers are each seeking to strengthen their position in the new world order by forming regional and international political and economic unions and blocs. Given the purpose of the research on financial convergence, it would be very important to pay enough attention to the economic structure of countries. To do so GDP growth indicators, monetary, financial and trade freedom, as well as the inflow and outflow of foreign direct investment are examined. These indicators show the level of economic development of each country. They are the strongest drivers and the most basic ones for convergence among financial markets. So, investigation of them is of particular importance.

- **Trade Freedom Index:**

The trade freedom index is determined based on two factors: the weighted average tariff rate and non-tariff barriers (including quantity, price, regulations, customs restrictions, and direct investment and government intervention).

**Fig 1:**
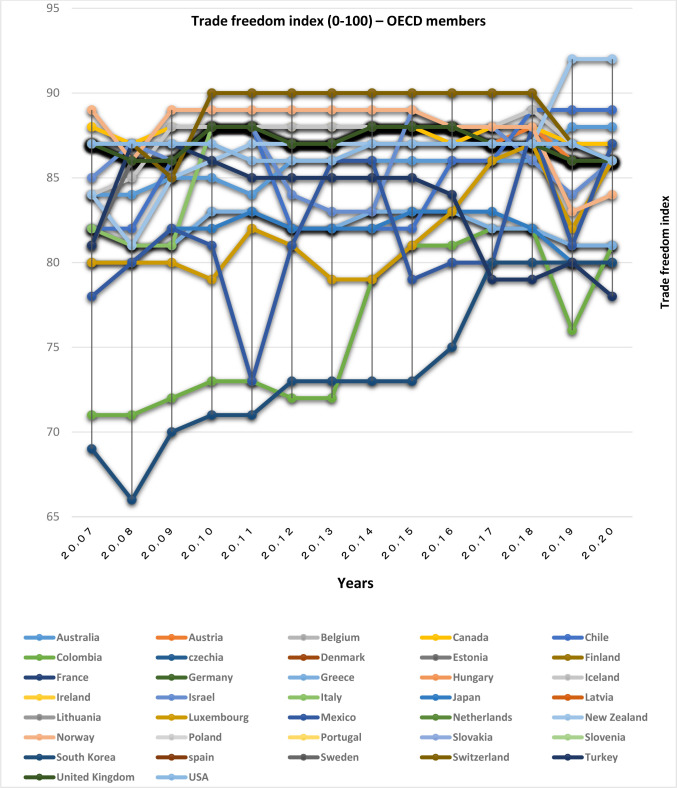
Trade freedom index (0–100), for OECD members from 2007 to 2020. The data are obtained from the Heritage Institute (Foundation). Source: Heritage Institute (Foundation).

**Fig 2:**
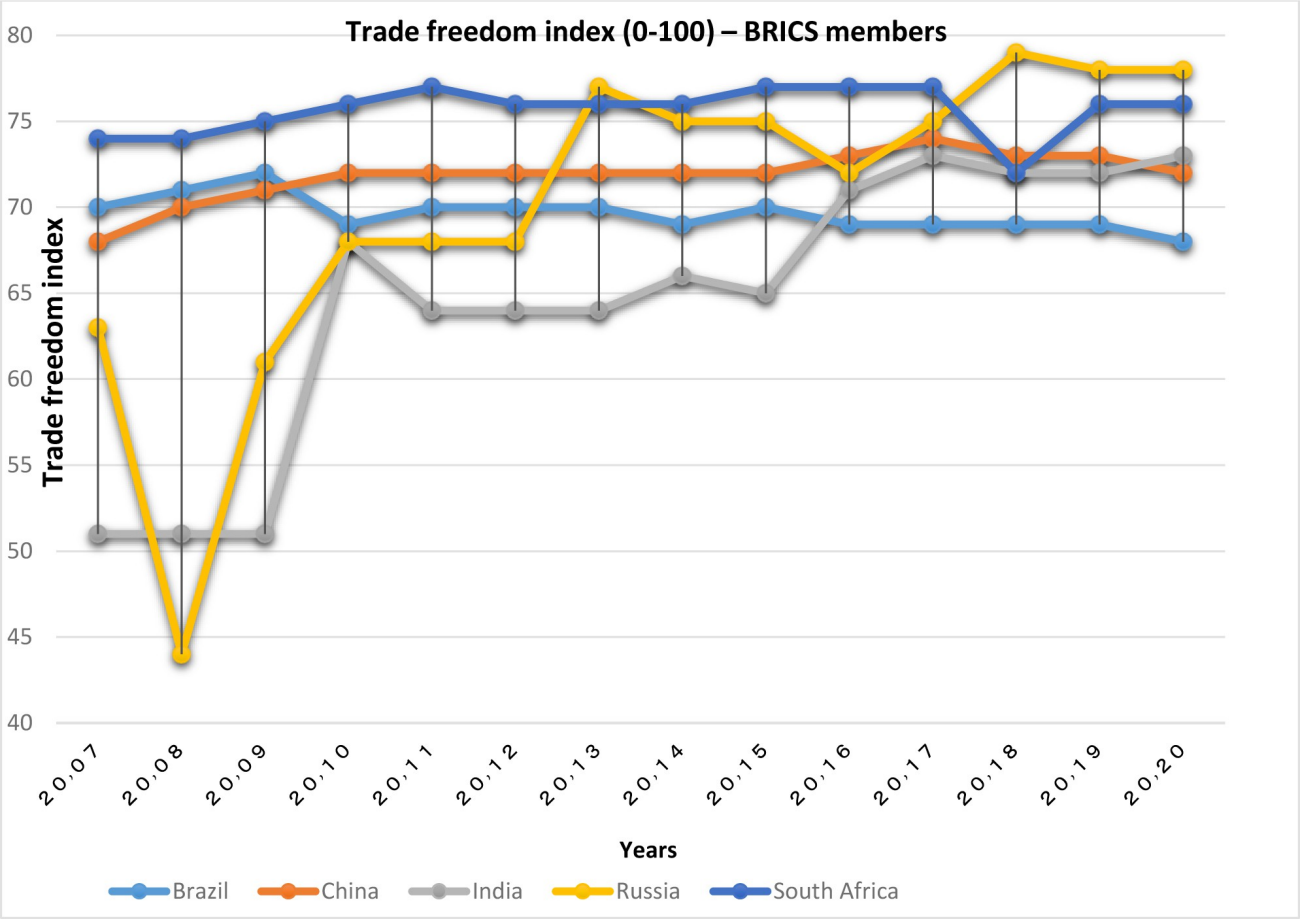
Trade freedom index (0–100), for BRICS members from 2007 to 2020. The data are obtained from the Heritage Institute (Foundation). Source: Heritage Institute (Foundation).

According to Figs 1 and 2, among the OECD countries, the lowest value of this index is related to South Korea and Colombia; the value of this index for other countries in this group is between 80 and 90. Among the BRICS countries, Russia and India, having removed trade barriers, have improved their trade freedom index since 2008. China, Brazil and South Africa are in a favorable position in this index. However, the trade freedom of OECD countries is higher than the BRICS group.

- **Monetary Freedom Index:**

The Monetary Freedom Index is investigated based on two factors: the weighted median inflation rate in the last three years, and price control.

**Fig 3:**
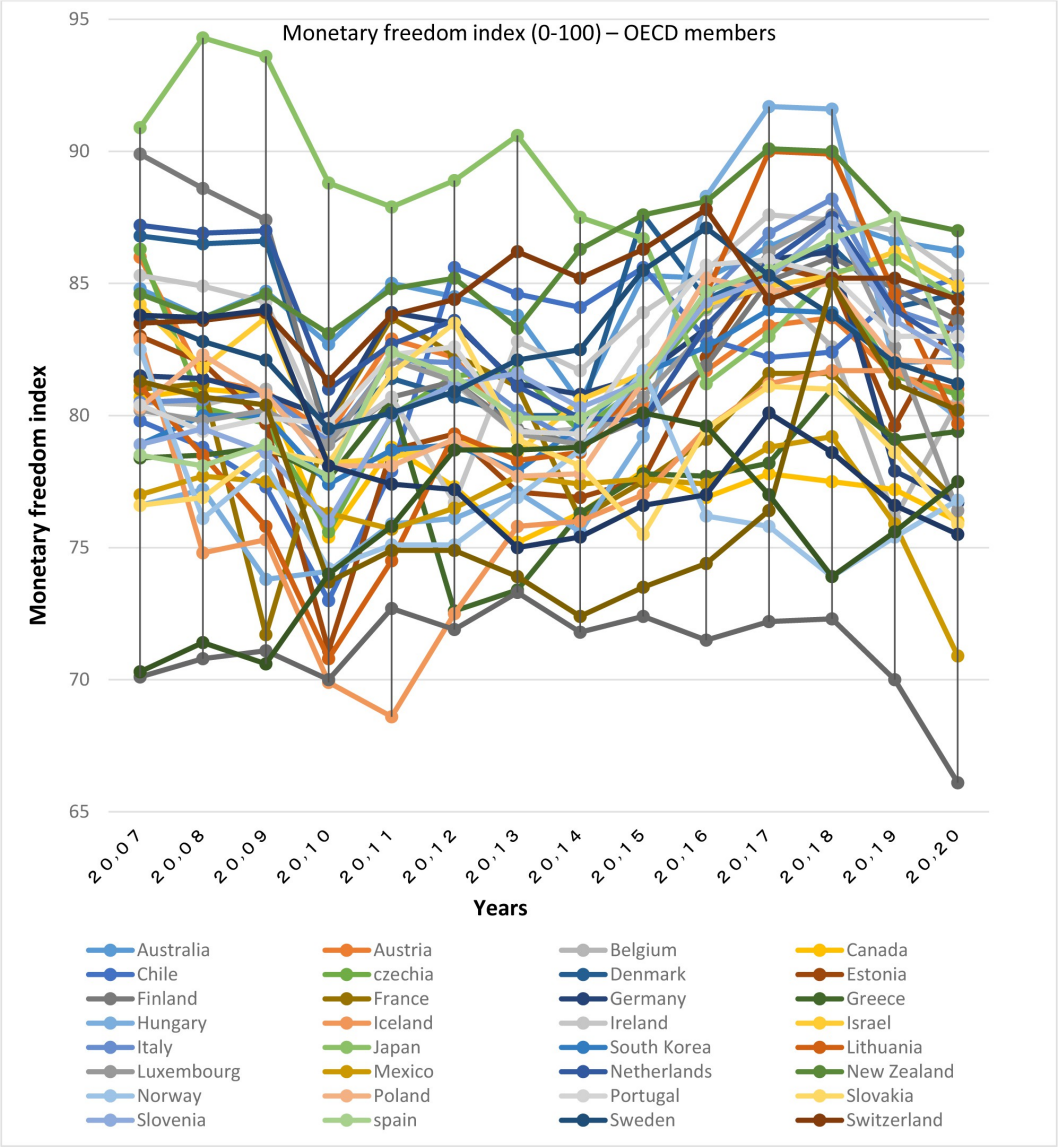
Monetary freedom index (0–100), for OECD members from 2007 to 2020. The data are obtained from the Heritage Institute (Foundation). Source: Heritage Institute (Foundation).

**Fig 4:**
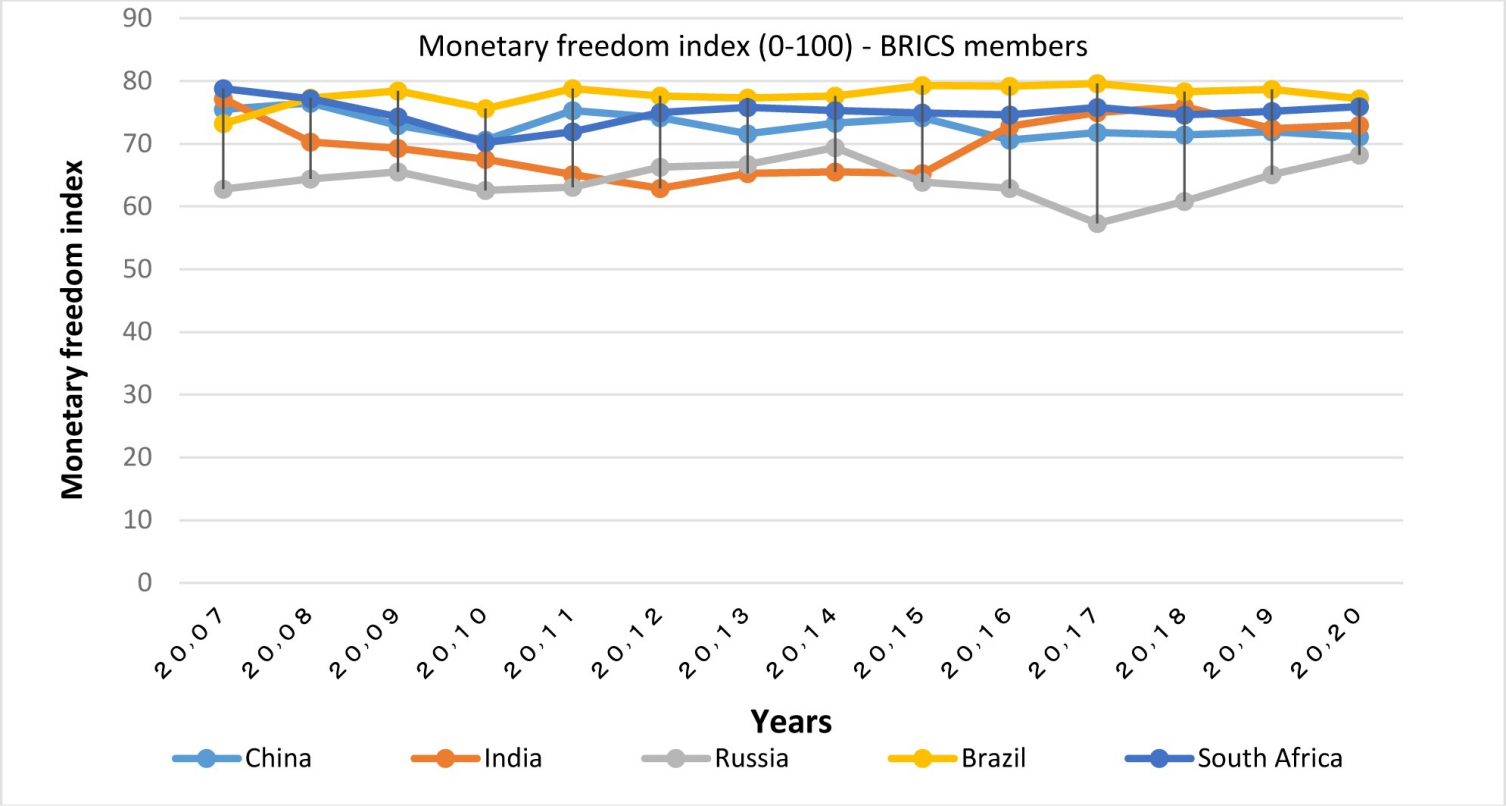
Monetary freedom index (0–100), for BRICS members from 2007 to 2020. The data are obtained from the Heritage Institute (Foundation). Source: Heritage Institute (Foundation).

According to Figs 3 and 4, OECD members are almost in the same range (in the range of 75–85) in terms of the monetary freedom index. The highest rates are respectively in Japan, Australia, Denmark, Switzerland, New Zealand and the lowest rate is in Turkey. Beside, with regard to this issue Hungary and Lithuania have made a significant progress in the recent years; though, the fluctuations in the index of monetary freedom is greater in BRICS countries (77–58), among which the highest value is related to South Africa and China, and the lowest is related to Russia.

Overall, a review of these charts shows that OECD members are in a higher position in terms of monetary freedom than the BRICS member countries. However the BRICS group is yet higher than the global average. It should be noted that according to [15], monetary freedom, being directly related to the development of the financial sector, reflects price stability, effective price control and business freedom.

- **Financial Freedom Index:**

**Fig 5:**
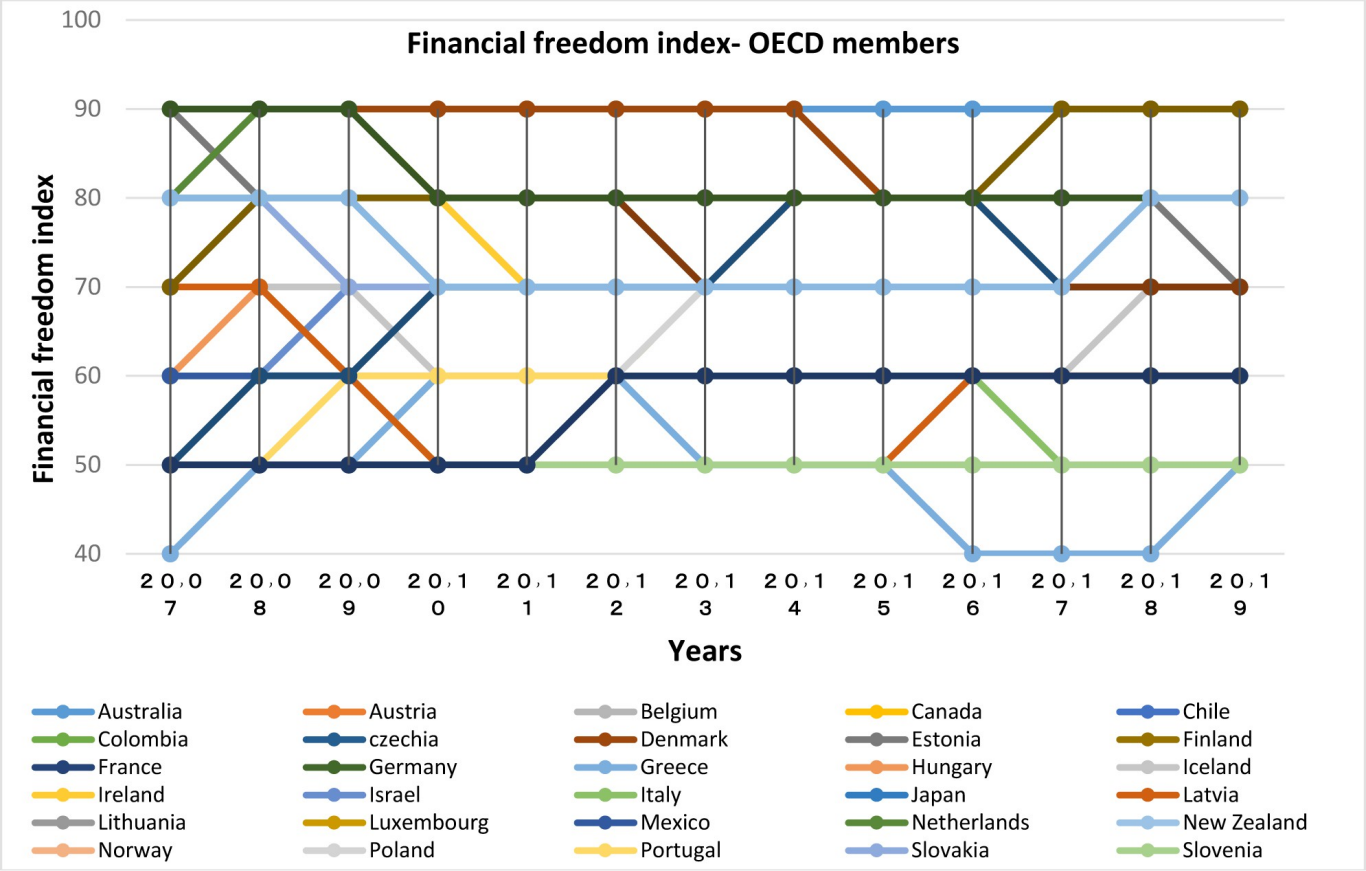
Financial freedom index (0–100), for OECD members from 2007 to 2020. The data are obtained from the Heritage Institute (Foundation). Source: Heritage Institute (Foundation).

**Fig 6:**
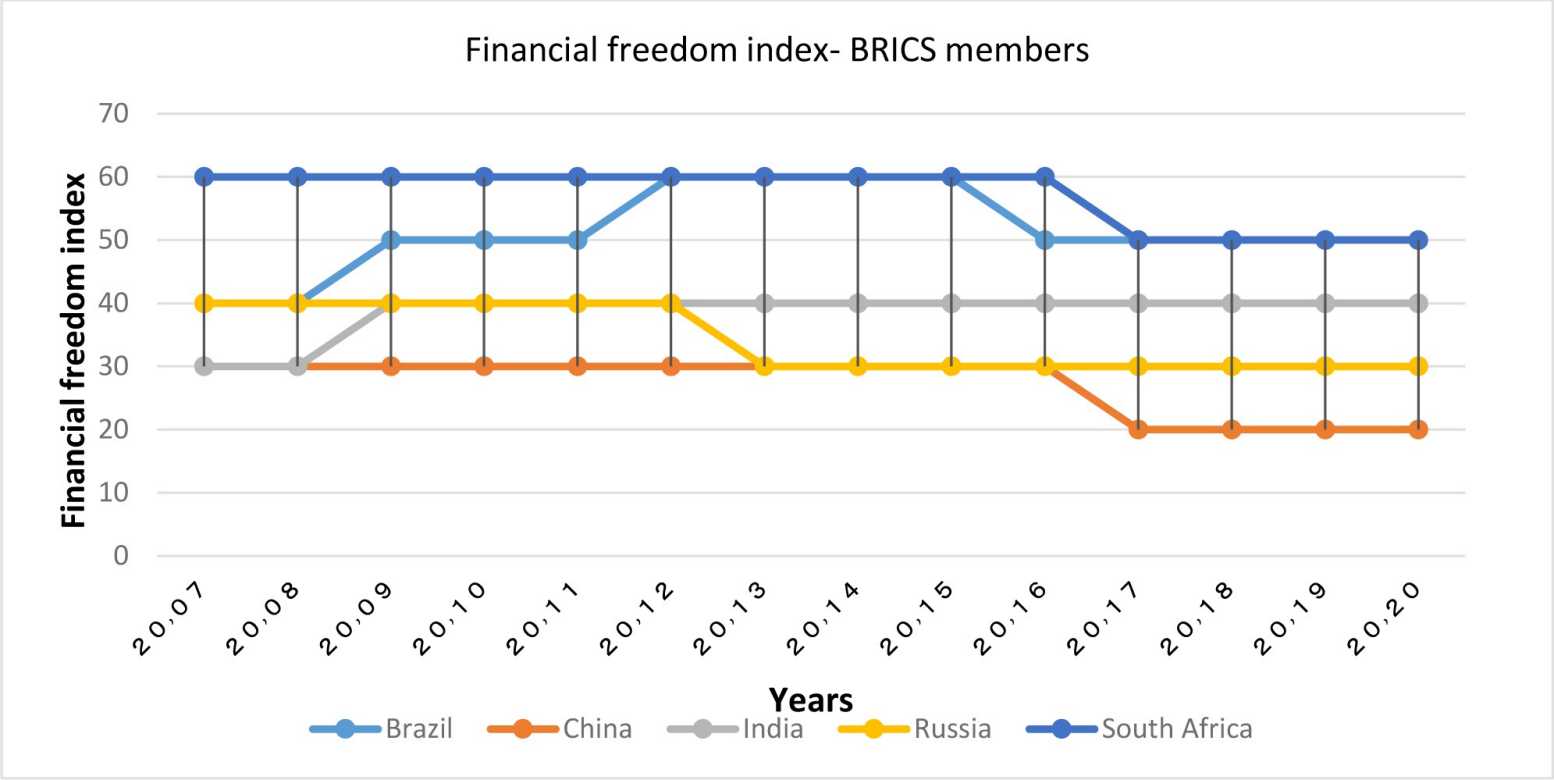
Financial freedom index (0–100), for BRICS members from 2007 to 2020. The data are obtained from the Heritage Institute (Foundation). Source: Heritage Institute (Foundation).

Figs 5 and 6 demonstrate that there is a high level of dispersion in terms of financial freedom among OECD countries, but most of them have the highest rankings in the world. The highest values of this index are attributed to Australia (first rank in the world in 2020), Denmark, Switzerland and South (Switzerland and South Korea has had significant advancements in the level of financial freedom with the index value of the former country changing from 70 in 2007 to 90 in 2017, and index value of the later country changing from 50 to 90). The lowest level of the index has been attributed to Greece and Slovenia. BRICS members have had a lower financial freedom index than the OECD group, with China and Russia having the lowest index, and South Africa and Brazil having the highest index. Examining the two groups, it might be stated that the level of financial freedom of OECD countries is above the global average (except Greece), while in the BRICS group, only South Africa and Brazil are at the global average.

Financial freedom is a measure of banking efficiency that indicates the degree independency of the banking system from government control, and also the degree of interventions in the banking sector; therefore, in countries with higher profiles in both groups, the role of central banks in countering banking fraud and designing facilitating rules is more significant.

In addition, financial freedom in the capital and credit market increases the efficiency of financial institutions, and decreases intermediation costs [15].

Analyzing the economic freedom indices mentioned, it would be evident that the monetary and financial freedom indices differ based on the level of development [16], the monetary and financial development rate in OECD member countries is higher than that in BRICS countries.

- **Investigation of capital inflows and outflows:**

Capital flows are important indications for financial integration, they significantly increase the development of financial institutions and market, and thus deepen the financial systems particularly in emerging economies [17]. Moreover, Countries having more financial convergence with other countries have higher levels of capital investments [8]. Therefore, it can be said that the relationship between capital flows and the convergence of financial markets is a two-way relationship.

**Fig 7:**
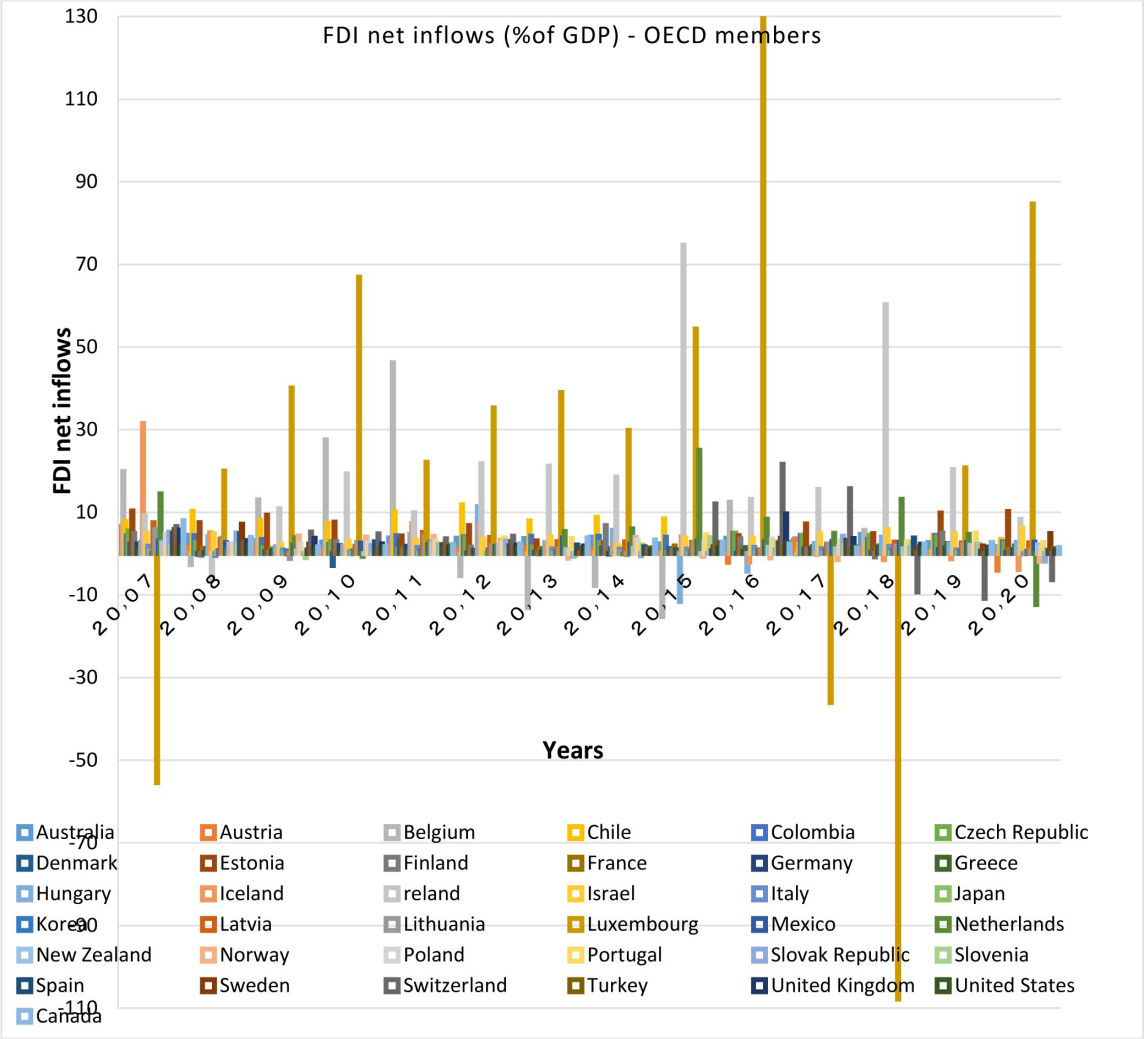
FDI net inflows (%of GDP) for OECD members from 2007 to 2020. This series shows net inflows from foreign investors, and is divided by GDP. The data are obtained from the World Bank. Source: World Bank.

**Fig 8:**
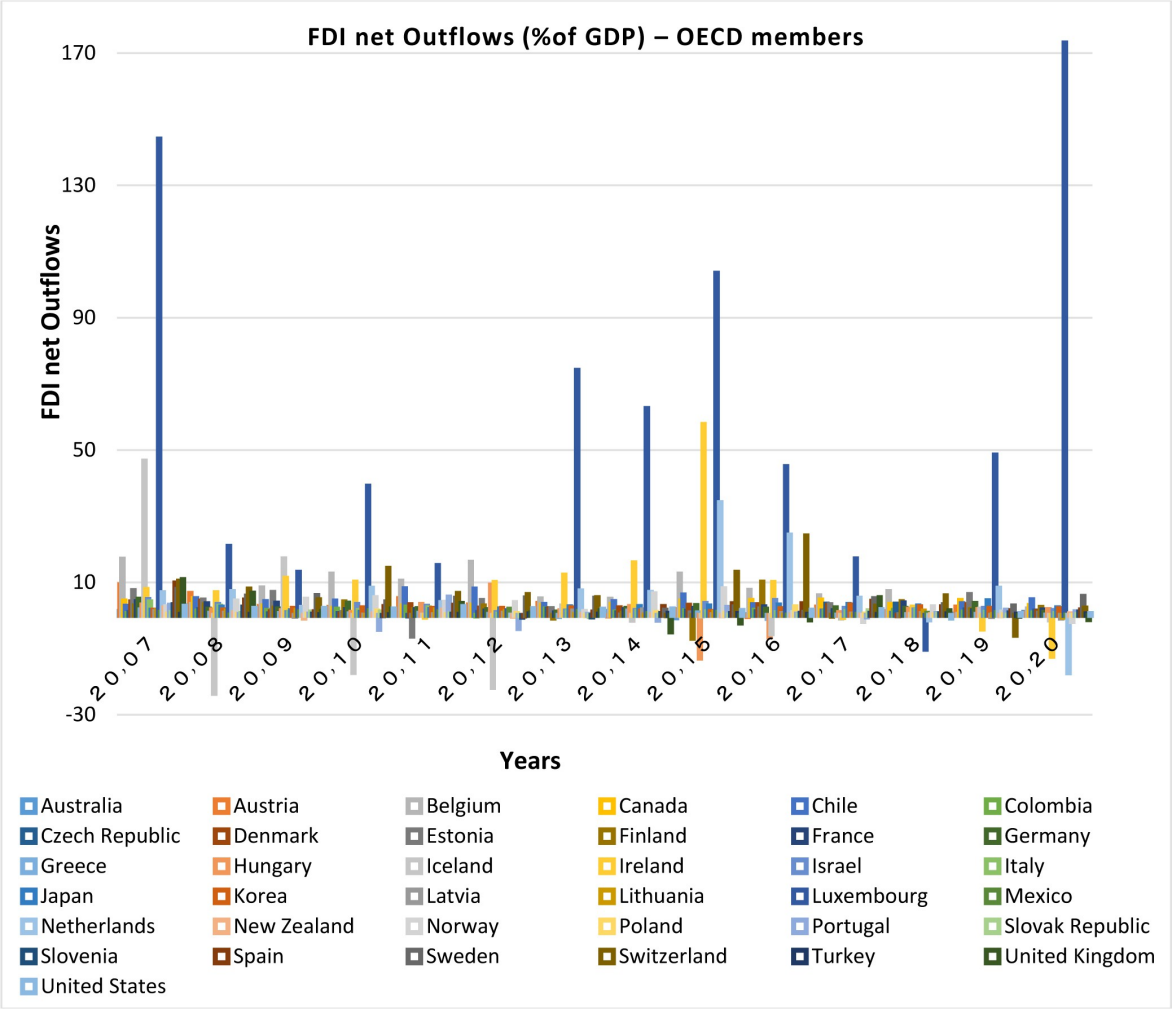
FDI net Outflows (%of GDP) for OECD members from 2007 to 2020. This series shows net outflows of investment to the rest of the world, and is divided by GDP. The data are obtained from the World Bank. Source: World Bank.

**Fig 9:**
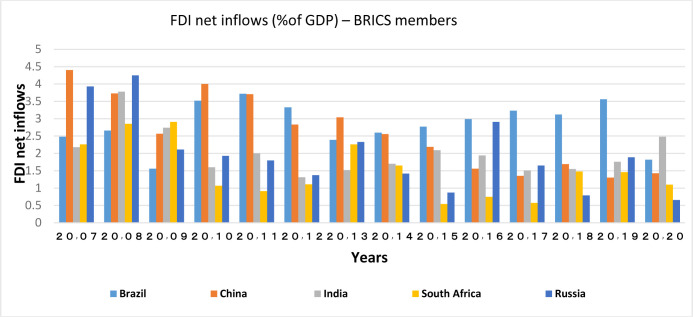
FDI net inflows (%of GDP) for BRICS members from 2007 to 2020. This series shows net inflows from foreign investors, and is divided by GDP. The data are obtained from the World Bank. Source: World Bank.

**Fig 10:**
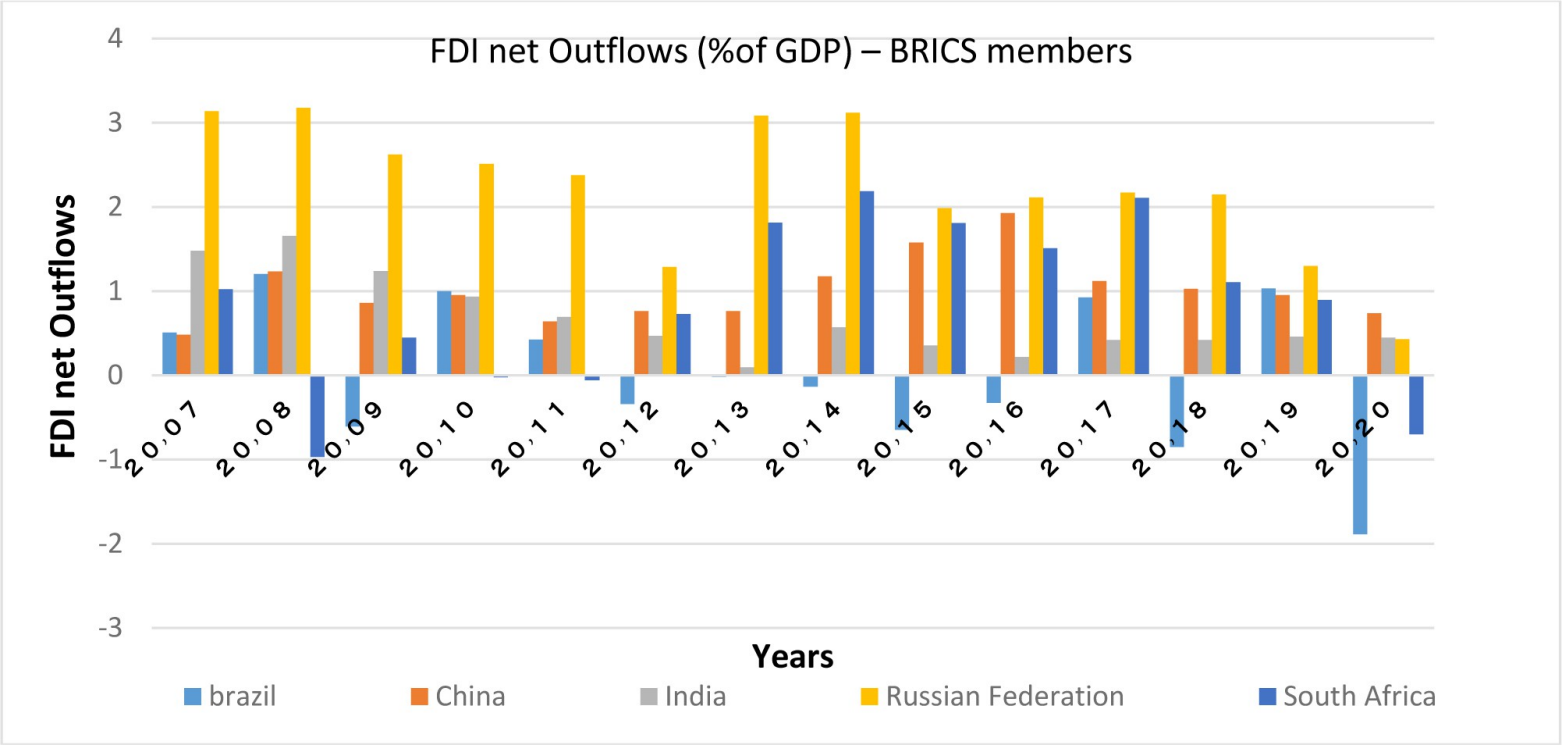
FDI net Outflows (%of GDP) for BRICS members from 2007 to 2020. This series shows net outflows of investment to the rest of the world, and is divided by GDP. The data are obtained from the World Bank Source: World Bank.

Capital inflows and outflows are almost always examined very accurately, because the capability of foreigners to invest in a country and also residents’ capability to invest abroad is of the theoretical concepts of financial openness. According to Figs 7–10, over the years under study, FDI inflows and outflows have been within the same range for OECD countries (excluding Luxembourg and Ireland fluctuations). BRICS countries have shown more volatility and dispersion in terms of capital inflows and outflows. Considering the charts, although in 2020, the outbreak of the Covid-19 caused a sharp decline in the inflow of capital to many countries of the world BRICS countries, except for South Africa, have recorded a positive rate in this regard.

FDI outflow also decreased by 48% in the OECD group. This decrease was mainly due to negative outflows from the Netherlands, and a significant reduction in capital outflows from Germany and Japan. Capital outflows were also negative in Ireland, Austria, Norway and the United Kingdom, but they were positive in Luxembourg and Sweden. In this regard, the United States is not included in the top 20 FDI Outflows, because the return on accumulated foreign income of its multinational companies has been high. Besides, the FDI output flow in the BRICS group has been declining (except in Russia). Even in Brazil and South Africa the FDI output flow has been negative. In general, the capital outflows in OECD countries has been much higher than that in BRICS countries.

- **GDP growth:**

**Fig 11:**
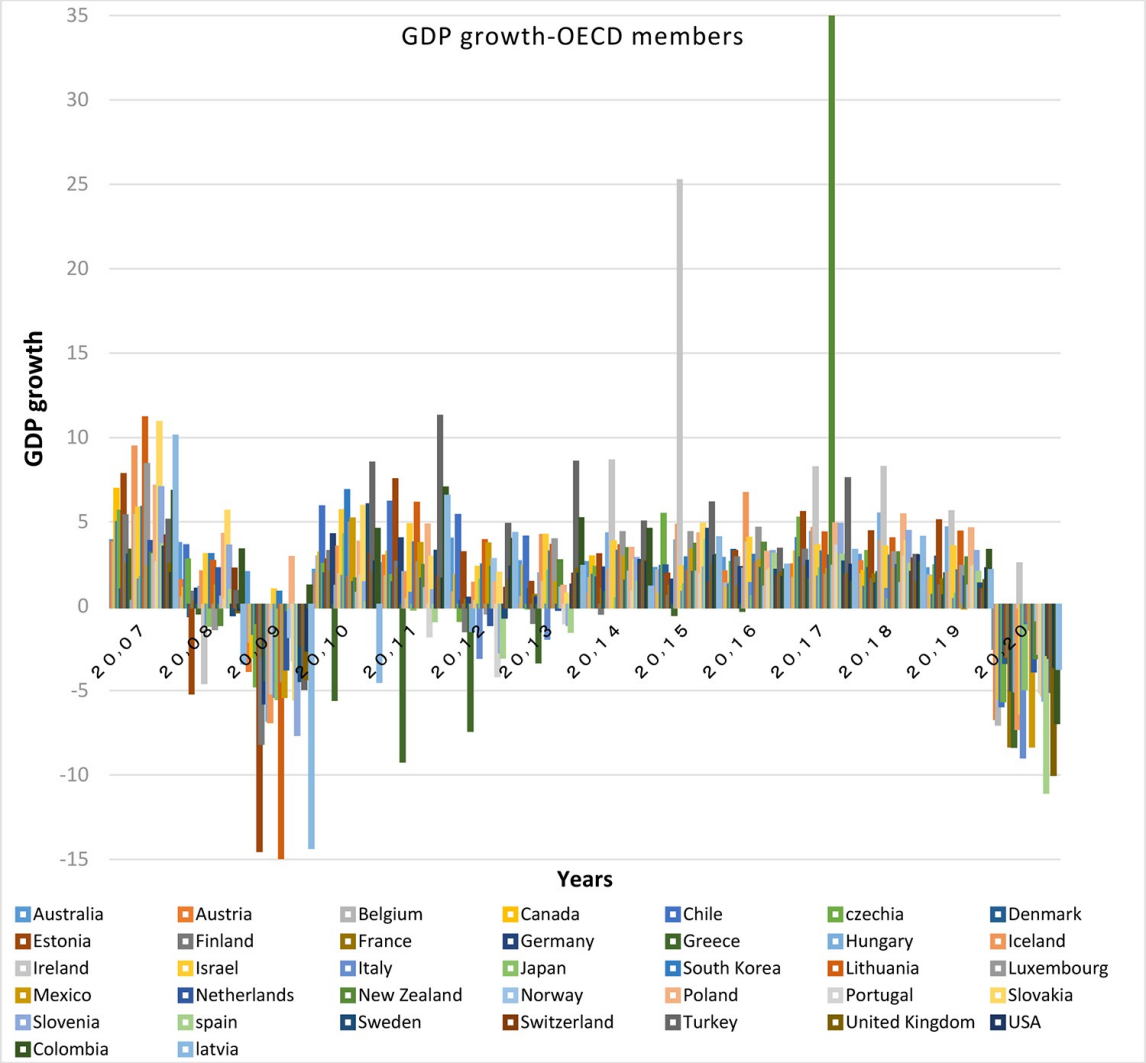
GDP growth-OECD members, from 2007 to 2020. The data are obtained from the World Bank. Source: World Bank.

**Fig 12:**
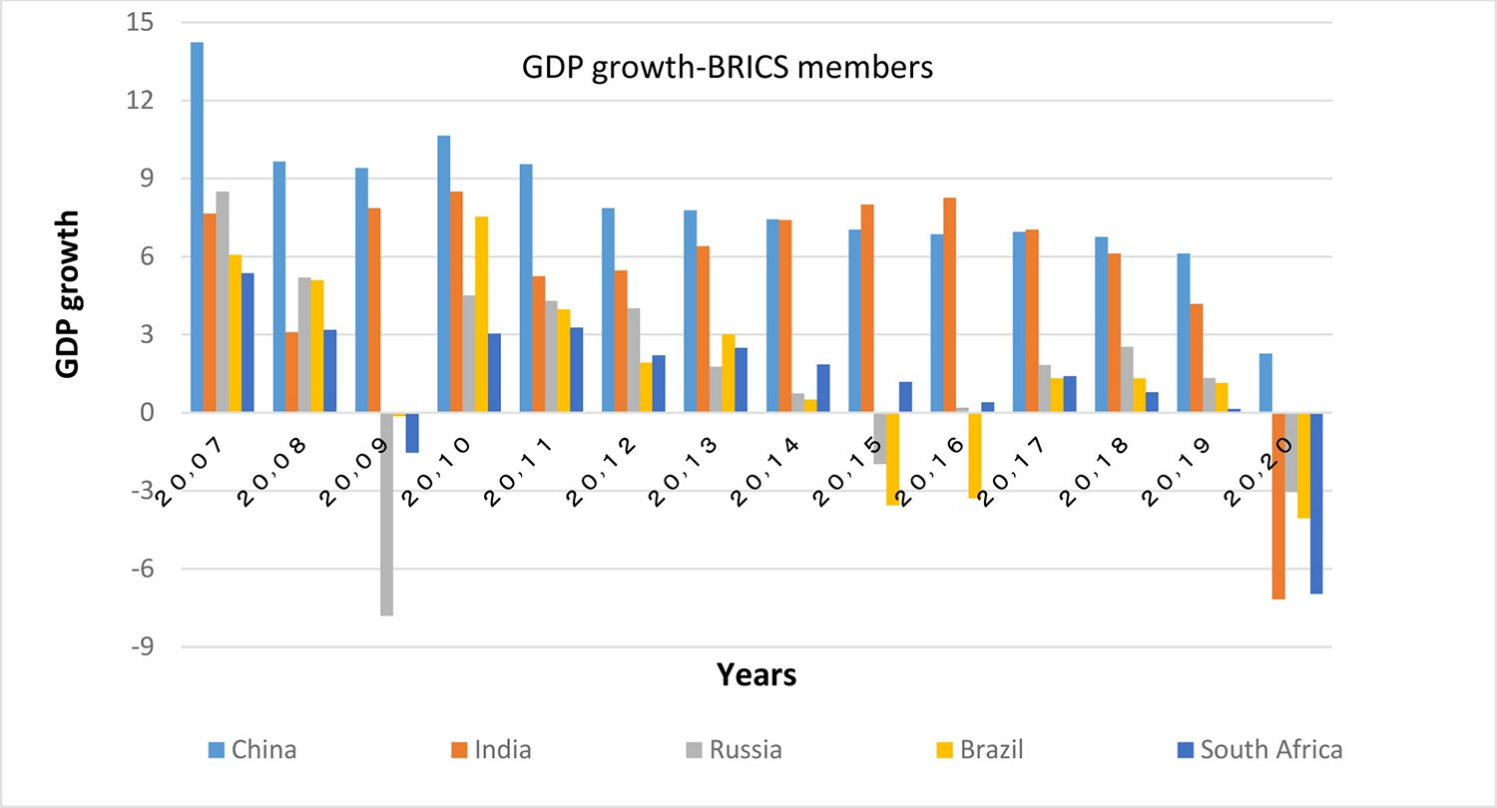
GDP growth-BRICS members, from 2007 to 2020. The data are obtained from the World Bank. Source: World Bank.

According to Figs 11 and 12, the distance between BRICS countries is more than that between the OECD countries in terms of GDP growth. Among them, China and India have the highest growth respectively. All BRICS members have acceptable GDP growth.

The presence of the four emerging BRICS countries among the top ten economies in the world can be attributed to several reasons, one of the main ones being that these countries have met the global standard, and their trade markets are expanding; Moreover, the OECD group and the economies of the member countries have been consistently ranked among the top countries in terms of GDP. Their names are still shining among the strongest economies in the world.

### 2.2. The concept of financial convergence

Over the past three decades, an increasing pressure on financial systems has been observed worldwide, mostly arising from liberalization and globalization. Financial convergence is an investment with positive net return rate that can be achieved by removing false trade barriers and agent movements. Numerous factors can provide the basis for financial convergence, among which factors such as the international capital inflows and outflows among countries, the presence of domestic markets in the international arena, the increase of banking relations among different countries, transparency increase and availability of clear and accurate information about markets, financial institutions of countries and legal and cultural infrastructures are the most important ones that can be mentioned [18].

#### Financial convergence is defined by two criteria

The first criterion is the cross-border financial activity. Thus, financial convergence can be defined according to the definition of the International Monetary Fund (IMF) (2008) “the level of relationship among countries through cross-border financial assets, and total gross foreign assets and debt to GDP”. This definition is the concept of financial globalization that states the greater the dependence of the financial markets of different countries on international assets and debts, the greater the convergence rate would be. According to this criterion, the presence of restrictions on the way of exchange or free access to the market, would prevent the free movement of capital and convergence.

The second criterion is the level of convergence and integration among markets. Financial openness, free access and the existence of financial and trade relations between countries is a necessary condition not enough for integration. Two markets might be totally open to each other, with yet imperfect integration; because the structure of the markets keeps them completely distinct. To put it more clearly, economic and legal structures, the existence of different rules and regulations of markets, and cultural differences are in the path of market participants like a steep and upward slope, which creates an incomplete convergence among markets [19]. This is while the most ideal form of convergence is absolute convergence; that is, similar assets in different markets and countries have exactly the same rates, but in practice what is formed in the real world is imperfect or incomplete convergence.

### 2.3. Research background

Convergence and integration of financial markets is considered as one of the most important changes in global financial markets and a document of globalization of economies. For years, researchers have been analyzing the convergence among financial markets. Each of the researcher based on their findings, has introduced effective or inhibiting factors of convergence. Most of them believe that the factor of widespread financial crises has an effect on convergence, but they do not agree on the type of this effect.

[19] Has claimed that the global financial crisis has led to stricter banking regulations, as a result of which global banks have left Latin America, also other emerging markets have experienced miss-treatments. These issues have reduced the economic prosperity of these countries, so strong investment and capital markets seem highly needed in them. These countries cannot easily contract in their financial markets, so improving financial integration can be a good solution. In this paper, researchers using the method of PS conclude that the analyses confirm the lack of financial integration in the seven Latin American countries. In other emerging markets, such as East Asia and Eastern Europe, financial integration is greater than that in the Latin America.

[20] State that although the convergence of global financial markets is a gradual phenomenon, some events accelerate this process. They show that after the financial crisis of 2008, the Latin American stock markets have experienced a higher level of convergence which has been revealed by the correlation among the annual returns of their stock market indices [21]. Also show that Latin American financial markets have become more convergent after the global financial crisis of 2008. It seems that not only in the case of Latin American financial markets, but also in the case of Asian financial markets the impact the financial crisis on convergence has been different:

[6] After examining the financial convergence in the Asian stock market, have concluded that there is both global and regional integrations. The speed of convergence has decreased after the financial crisis in 2008 [22]. Have examined the effect of financial integration and economic growth in 33 Asian countries from 1980 to 2015. It was shown that the financial integration for volume-based and equity based measures significantly effects economic growth in Asia during the pre-crisis period. After the crisis, there would be no relationship between financial integration and economic growth; [23] by examining the convergence of Asian stock markets via the [12] test found that in the pre-crisis period there was no convergence at the Asian stock markets; by contrast, in the post-crisis period the Asian markets have appeared to be integrated both at global and regional level.

Other researchers have also investigated the existence or non-existence of financial convergence in different countries and economic groups. The following are some of the most important of these studies, which their researches include to some extent the countries studied in this article:

[24] investigated the financial convergence among Central and Eastern European countries (CEE) for the period of 2007 to 2014 via the PS method. Findings indicate that CEE financial markets do not form a convergence club. The gap in financial markets has also widened since the global financial crisis. To achieve financial convergence, the authors suggest that CEE countries make further structural reforms; besides, [25], using the OTC index, confirm the process of divergence in the financial markets of CEE countries during the global financial crisis.

The research of [26] shows that some of the Eastern European countries (Estonia, Latvia, Lithuania, Slovakia and Slovenia) that joined the Eurozone during 2007 and 2015, have achieved a significant financial and economic convergence, due to the changes they made with regard to improving the institutional quality of countries and determining to strengthen relations with the west using the beta convergence method, [27] in their convergence process examined the financial crisis as a turning point in the evolution of financial systems in the EU. They have claimed that in the process of leveraging the economy, this crisis is a turning point in the growth of banks and. Dynamic systems can be interpreted as credit-centric bubble collapsing with the crisis, leading to a decline in economic influences. Researchers believe that convergence in European countries is now greater than that before the crisis [28]. States that after the introduction of Euro in 1999, the Eurozone countries gradually became more integrated financially. But this trend was completely reverse with the onset of the financial crisis in 2007; nevertheless, policy interventions in 2012 helped stabilize financial markets and gradually improve financial integrity. [29] for the period of 1963 to 2012 examined whether banking actions and stock markets have undergone a convergence trend among EU countries or not. And also whether the transition from the European monetary system to the single currency has led to financial integration in the last 5 decades or not. The results of this study has shown that banking and stock market actions in Europe are converging over time. For European countries in the Eurozone and European countries outside the Eurozone for the period of 2001–2017, [30], using Sigma convergence, examine whether European financial systems have undergone a convergence trend or not. They concluded that these countries converge in investment funds and equity, bank loans, and trade credits respectively.

As the process of financial integration in the European Union has been successful in most cases, thus it would be better for the European Union to accelerate financial integration.

[5] Examined the convergence of financial assets in OECD countries for the period of 1980–2005 using Beta and Sigma convergence tests. The results confirmed beta convergence. The existence of beta convergence without Sigma convergence means that financial systems are likely to remain unique [31]. Tested the hypothesis of convergence of stock market price index in the OECD group of countries for the period of 2007–2017 by the PS methodology. The results of the study indicated lack of general convergence of the studied stock markets. [32], after investigating the hypothesis of financial convergence in OECD countries, found no significant evidence of convergence over time. Their results have indicated that while the behavior of the world’s largest active financial institutions is converging irrespective of home domicile, their activities are not necessarily leading to the general global homogenization of financial forms and financialization convergence hypothesis.

[33] Conducted a study analyzing financial convergence in insurance companies and pension funds using the Sigma convergence method. They considered 12 American companies, 10 European companies, and 8 Asian companies using annual data from 2012–2019. Researchers found that the financial convergence of global pension and insurance sectors has brought significant changes to the global financial system. They believe that in recent years, financial intermediaries like insurance companies and pension funds, as strategic investors, have increased their influence in global markets. Thus, they have been given the opportunity to stabilize the financial systems of developed countries and speed up the formation of stock markets in transition countries.

In recent years, emerging countries have been heavily discussed in the conversations about the convergence of financial markets. These countries have recognized that their economic growth, progress and development are closely linked to the convergence of their financial markets, particularly with developed countries. This is important for international investors looking to expand the geographic scope of their portfolio diversification strategies [34], using the fractional integration framework, have examined the long-term and short-term relationship among the BRICS real estate investment trust markets and the United States, Australia and the United Kingdom for the period of 2012 to 2017. The results indicate that there is no convergence among the studied markets and only in the short run the financial markets are affected by the markets of developed countries. They also found that when the market portfolio of developed countries is added to the BRICS market portfolio, investor risk is reduced as the diversity is increased. on the other hand, [35] by evaluating the financial convergence of APEC countries, which is a combination of developed, emerging and developing countries, have concluded that although Banking markets are divergent, these countries have achieved significant convergence in the stock market. They consider the reason for the divergence to be the diversity and heterogeneity of the APEC member countries, ranging from highly developed and wealthy nations to low-income countries. Also they believe that the convergence of the stock market can be associated with direct foreign currency flows to these countries [36]. Evaluated the stock market integration of emerging Asian countries by examining long-term relationships among markets using Gregory and Hansen’s co-integration tests and detrended Cross Correlation coefficients. The results indicate that all emerging markets show evidence of both global and regional integration.

[37] Examined the impact of globalization and financial integration on the economic growth of China, India and Brazil. Researchers in the current research conducted the study using the Johansen Integrated Unit Root Test and the Causality Test for the period of 1990–2016. The results revealed that there is a long-term relationship among all indicators for the three countries, but errors were only corrected for Brazil and China. It was found that in China, the factors of financial integration and globalization influenced per capita GDP, whereas this relationship was not observed in Brazil and India. If the financial convergence among multiple countries is confirmed, it raises the question of how this convergence could affect macroeconomic variables.

[8, 17, 38, 39] in their research have shown that financial integration and liberalization might have a positive and significant effect on economic growth; as financial convergence plays a vital role in economic growth via sharing the risk between countries, cross-border capital association, investment and financial information, [40]. Furthermore, the increase of stock market liquidity and possibility of the presence of more foreign banks, which increases the efficiency of the domestic banking system, promote economic development by accelerating productivity growth. [41, 42], state that although the integration of financial markets has a positive effect on economic growth, this effect is insignificant and can be ignored. [43]; conversly, economists like [44, 45], by addressing issues such as the informal financial market, market failure, and asymmetric information, have pointed out the negative effects of financial co-integration on economic growth. The findings of [46] confirm that the relationship between the integration of financial markets and China’s economic growth is negative in the long term, and financial integration imposes a potential risk on the economy by limiting economic growth. [47], also believes that the impact of financial integration on the entire macroeconomics being insignificant might increase the risk of financial crisis contagion among countries. Other researchers such as [48, 49] in their research done for a large number of countries have concluded that the effect of financial integration on economic growth is different, because it depends on various structural factors.

According to the findings of [50], financial integration can have a negative or positive effect on economic growth, which completely depends on other variables such as the level of financial development, economic development, human capital, institutional quality, and macroeconomic framework. Examining the studies conducted by researchers, it can be seen that empirical evidence does not provide similar results in terms of financial convergence. So far no scientific study has specifically examined the financial convergence between the OECD and BRICS groups. In fact, further research in this area seems to be required.

## 3. Research method

Most convergence testing studies have examined convergence through three methods:

1. Beta convergence is divided into two groups: conditional beta and absolute beta. A) Absolute convergence occurs when economies are driven to a steady state with different growth rates, where the differences depend on their initial conditions. B) Conditional convergence, on the other hand, takes into account the different economic structures of countries, resulting in different levels of steady state. Each economy tends towards its steady state, and the further it is from this state, the higher the growth rate. 2. Sigma convergence is based on the standard deviation behavior of variables over time, serving as a necessary condition for reducing fluctuations of variables over time. This concept was first introduced by [51–53]. 3. The third method is random convergence that was proposed by [54], as well as in [55–57]. In this type of convergence, the test is based on equations with a random trend. The unit root test was also proposed by [58–60] to test the convergence hypothesis that tests the data trend with and without differencing [61, 62].

But [13] stated that beta and sigma convergence were more suitable for growth models. Authors i.e. [56, 63] also point to some of the problems associated with these tests, particularly in producing uncertain and biased results; first, the concepts of growth models for "absolute" convergence and "club" convergence are not explicit. Second: Different tests such as testing the null hypothesis are not necessary and therefore are not directly comparable. Third: Most tests are based on specific and limited assumptions about the structure of the panel [31]. When there are several stationary trends in the data, unit-root co-integration methods fail either [14].

In this research, the panel club convergence, and the cluster method proposed by PS are used which is a relatively new methods. In essence, this method tests whether the dispersion among the cross-sectional units of the variable used decreases over time or not. Compared to other convergence tests, i.e. the Sigma or Beta convergence tests, this test has the advantage of being more general in some respects. No specific assumption states the stability of the variable in question or the existence of the required common factors. The advantages of the mentioned method might be presented as follows; first, there is no need to provide preconditions of the classical models. Second, this method, using a nonlinear time-varying factor model, examines convergence. Third, this approach includes the experience of countries in transition dynamics, while the hypothesis of homogeneous technological progress, which is a widespread assumption in most growth studies, has not been considered [12].

### 3.1. The nature of the Phillips and Sul cluster method

In the Phillips and Sul’s approach (2007), known as the log(t) regression convergence test, convergence occurs as in the form of decreased distance, and scattering among the sections occurs over time. The general framework of the model is as follows, in which a panel model for the variable Xit where i = 1, 2… N and t = 1, 2, …, T denote the number of units and the sample size.

The following simple linear factor model is the starting point of the test:

$$X_{it}=\delta_{i}\mu_{t}+\varepsilon_{it}$$ (1)

Where, *ε*_*it*_ and *μ*_*t*_ are unobservable components. PS modified the primary model as follows:

$$x_{it}=\delta_{it}\mu_{t}$$ (2)

And the common factors are separated from the idiosyncratic components as follows:

$$x_{it}=g_{it}+a_{it}$$ (3)

Where g_it_ represents systematic components and a_it_ denotes transitory components. To separate the common and non-systematic components in the panel, PS modified the equation as follows:

$$X_{it}=\left(\frac{g_{it}+a_{it}}{\mu_{t}}\right)\mu_{t}=\delta_{it}\mu_{t}$$ (4)

In other words, x_it_ has been decomposed into a common component "*μ*_*t*_" and a non-systematic component *δ*_*it*_", both of which are time functions and measure the economic distance between the trend component and *μ*_*t*_ and x_it_. To test the convergence of components *δ*_*it*_, [12] defined the transition element *δ*_*it*_ by the following equation:

$$h_{it}=\frac{X_{it}}{\frac{1}{N}\sum_{i=1}^{N}X_{it}}=\frac{\delta_{it}}{\frac{1}{N}\sum_{i=1}^{N}\delta_{it}}$$ (5)

The variable h_it_ is called the relative transition parameter and defines a unique path for each country i relative to the panel average. Thus, h_it_ actually examines the kinetic growth trend of country i around the fixed point *μ*_*t*_. The transition curve average for all countries is equal to one for each set of data at any time. Therefore convergence is obtained when *h*_*it*_→1 for all I, as *t*→∞. In the case of convergence within the clubs, the transition paths narrow to different steady-state equilibria, which can be above or below the crosssection average of one.

[12] proposed log(t) regression to test the null hypothesis of convergence, they introduced two concepts of convergence, namely absolute convergence and relative convergence. The following null hypothesis is presented for the concept of relative convergence:

$$H_{0}:\delta_{it}=\delta \;\alpha \geq 0$$ (6)

The opposite assumption will be as follows:

$$H_{1}:\delta_{it}\neq \delta \;\alpha <0$$ (7)

For the concept of absolute convergence, the null hypothesis is presented as follows:

$$H_{0}:\delta_{it}=\delta \;\alpha \geq 1$$ (8)

Then, in order to estimate the log(t) test, in the first step the cross-sectional variance ratio H_1_/H_t_ must be calculated:

$$H_{t}=\frac{1}{N}\sum_{i=1}^{N}{\left(h_{it}-1\right)}^{2}$$ (9)

The following OLS regression is performed as follows:

$$log\left(\frac{H_{1}}{H_{t}}\right)-2log(log(t))=\hat{a}+\hat{b}logt+{\hat{\mu }}_{t}$$ (10)

[12] Recommended that regression gets started from a point such as t. that t = [rT], [rt] + 1,… T and (r> 0). The main parameters of the convergence test i.e. b depend on a. Accordingly, PS estimated the full value of logt equal to $\hat{b}=2\hat{\alpha }$, where $\hat{\alpha }$ is an estimate of the coefficient α under the null hypothesis [12]. Determined r = 0.3 in case of T≥100 and r = 0.2 for T≤50, based on their simulation experiments. If t-statistic of the coefficient b at the level of 5% which has a normal distribution, is less than the critical value of -1.65, the null hypothesis of convergence would be rejected.

In this method, the rejection of null hypothesis does not imply the absence of convergence in the subgroups of the panel. There might be cluster or group convergence among countries. To determine a club convergence, PS proposed a clustering algorithm, which is described in detail in the next section. This method, although being simple, makes it possible to calculate general convergence, overall divergence, convergence in subgroups, and divergence in units.

### 3.2. Cluster algorithm for determining club convergence

According to PS’s log-t test (2007), considering some definite values, a simple algorithm has been proposed in order to sort the panel into convergent subgroups. This algorithm consists of four steps:

#### Step 1: Sorting by last observation

N sections in the panel are sorted in the descending order based on the last *X*_*it*_ observation.

#### Step 2: Core group formation

Based on the calculation of the logarithm t for k members in the panel, where the condition 0 <k <2 is met, the principal convergent members are calculated using the statistic $t_{\hat{b}}(k)$ and the log-t regression fitness. The group core size is calculated by maximizing $min\;t_{\hat{b}}(k)>-1.65$. This process continues until the value of tb^^ is greater than -1. 65. by obtaining all values less than -1. 65 the core of the group is formed with k* = *k*−1 members.

#### Step 3: Adding each section sequentially to the core group

When the core of the group is formed, each of the remaining sections is added separately, and the logarithm t would be calculated. New sections are included in the group if $t_{\hat{b}}>c$, where c is the critical value (0≤c). This process is implemented with the aim of preserving the sections and creating the first convergence club.

#### Step 4: Recursion and stopping rule

Establishing a subgroup of converging units, all remaining units are tested jointly for convergence; In other words, the second group consists of all units outside the first group. If the null hypothesis ($t_{\hat{b}}(k)>-1.65$ is not rejected, there would be an additional convergence subgroup in the panel. If the null hypothesis ($t_{\hat{b}}(k)>-1.65$ is rejected, steps 1,2 and 3 would be repeated for the remaining units. If no other subgroup is observed, it could be concluded that the remaining units are divergent.

## 4. Data

The statistical population of this study includes the member countries of the Organization for Economic Cooperation and Development (OECD) and also the BRICS countries that were studied in the period 2007 to 2020. The reason for selecting the start of the review period is the financial crisis of 2007 and taking into account the impact of this year on the global financial markets. This study focuses on two specific financial sectors: the money market and the capital market. The variable of Bank credit to the private sector is used to examine the financial convergence in the money market. Bank credit to the private sector is considered as one of the most important indicators of the financial market. The availability of credit, facilitating new investments, is essential for the private sector, as it enables the transfer of deposits and savings to the crucial sectors of the economy.

The stock market capitalization as a percentage of GDP is used for evaluating the convergence in the capital market, which is determined by the total value of shares available for listed domestic companies on the stock exchange. Exceptions include investment funds, unit trusts, and companies the sole business purpose of which is to hold shares of other listed companies.

## 5. Model estimation

Since convergence is a long-run concept, the hodrick and prescott filter is used to extract the trend component from the set, as recommended by [12]. Then the general process of the data is used to examine convergence.

### 5.1. Drawing the relative transition path

**Fig 13:**
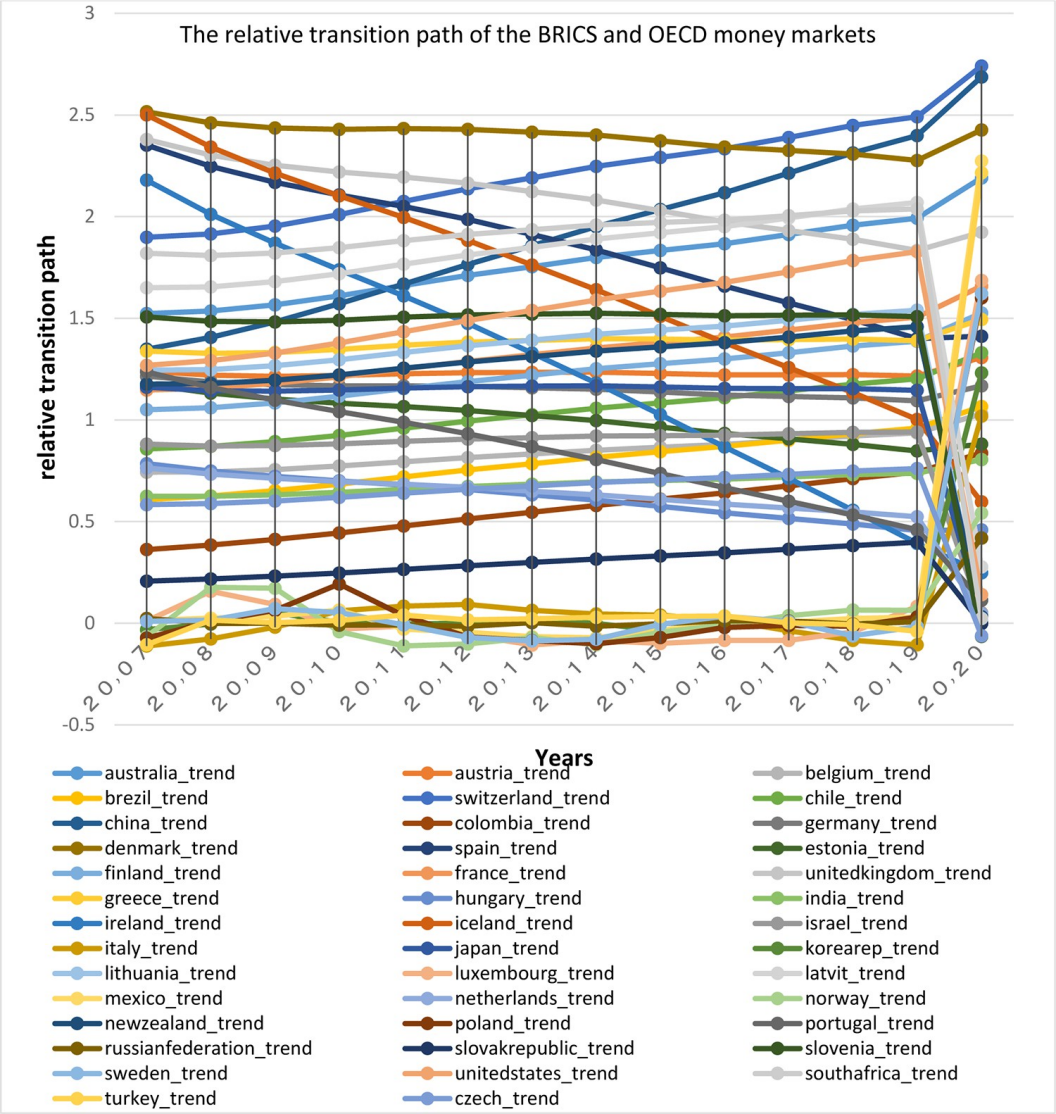
The relative transition path of the BRICS and OECD money markets, from 2007 to 2020. The relative transition path (h_it_) are calculated according to Eq 5 for the money markets of the two groups of OECD and the BRICS. Source: research findings.

**Fig 14:**
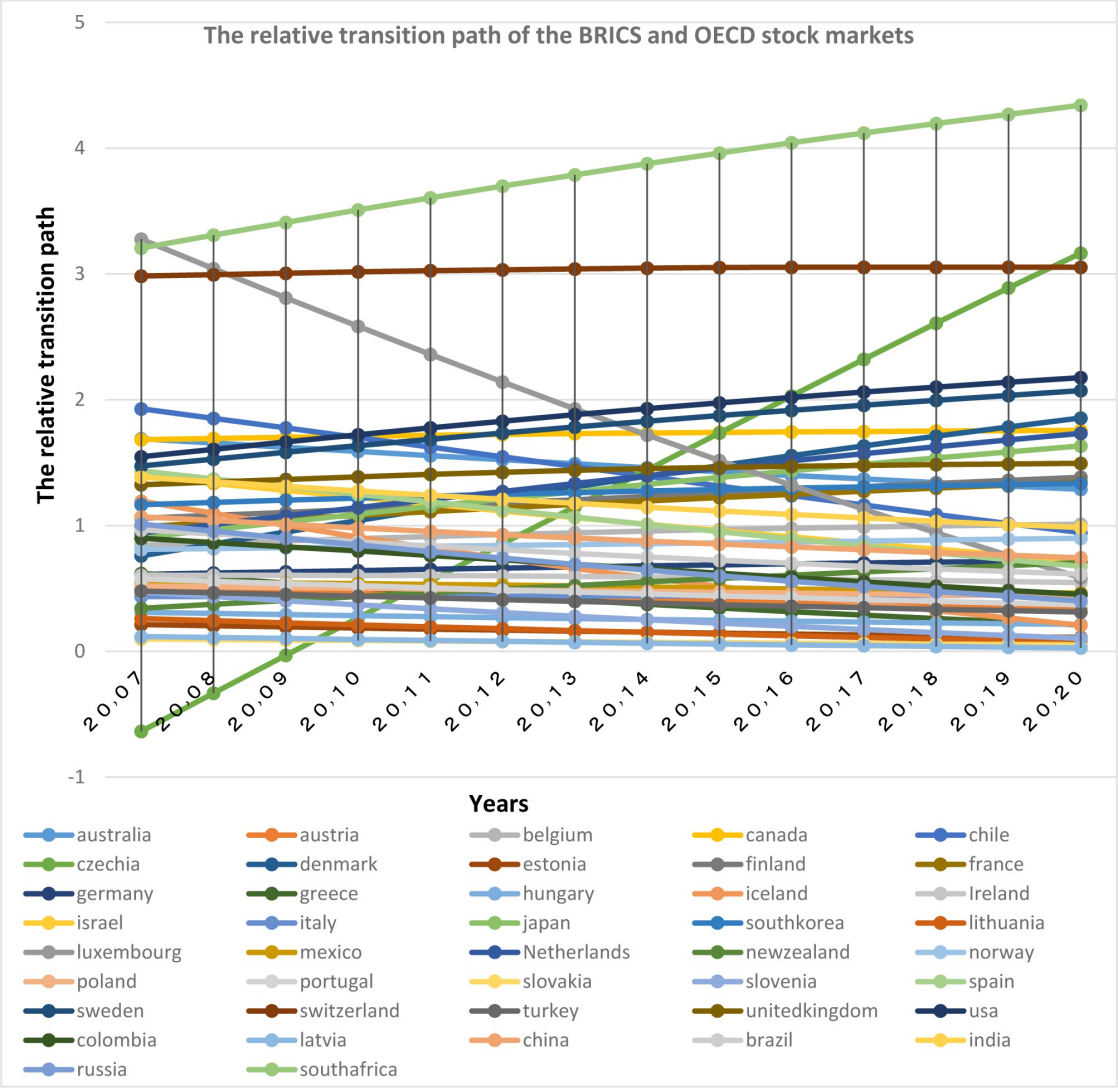
The relative transition path of the BRICS and OECD stock markets, from 2007 to 2020. The relative transition path (h_it_) are calculated according to Eq 5 for the stock markets of the two groups of OECD and the BRICS. Source: research findings.

First, the relative transfer parameters (h_it_) are plotted according to Eq 5 for the money and capital markets of the two groups of the Organization for Economic Cooperation and Development (OECD) and the BRICS in the period 2007 to 2020. Convergence occurs when the hit parameter of countries converges to number one; therefore, observing the relative transfer path diagrams might give researchers a view on the presence or absence of convergence. As can be seen relative transition diagrams (Figs 13 and 14) are not converged to number one. Thus, it can be concluded that there is no overall convergence among the selected financial markets. However given the closeness of the charts of some countries, it can be said that a group of countries tend to have convergence.

### 5.2. Estimation of financial convergence model in money market

After examining the relative transfer path of the variables, the log-t regression equation is then fitted for general estimation of the convergence. Table 1 provides the results related to the convergence of the Bank credit to the private sector index for both BRICS and OECD countries. The t-test results indicate that the null hypothesis of convergence is rejected, as the t-test value is lower than the critical value of -1.65. This suggests that based on the studied index, there is no overall convergence among all sample countries. Subsequently, the study examines the possibility of cluster convergence among countries and are classifies them based on the t-statistic. According to Table 2, the results of the club clustering algorithm for the money market index shows that there are five converging clubs during the period under study. Then, at the suggestion of [12], the inter-cluster convergence test is performed. In this test, if the null hypothesis is not rejected, the relevant clusters might be merged into a larger cluster. The estimation results indicate a possibility of merging between the first and second clusters as well as the third and fourth clusters, so the number of clusters is reduced from 5 to 3.

**Table 1:** The result of overall convergence estimates of the money market between the BRICS countries and the OECD.

	Coefficient	T- Statistic	S.E
Logt	-0.99	-22.91	0.043

Note: The null hypothesis of convergence is rejected at the 5% level if tb < 1.65

**Table 2:** Classification of money market convergence clubs between the BRICS and OECD countries.

Club	Countries	Coefficient	T- Statistic
Club1	Switzerland, China, Denmark	2.4	3.04
Club2	Australia, United Kingdom, South Korea, Norway, New Zealand, Sweden, Germany	0.52	5.74
Club3	Greece, France, Finland, Spain, Colombia, Chile, Slovakia, Portugal, Netherlands, Luxembourg, Japan	0.68	5.88
Club4	India, Estonia, Brazil, Belgium, Austria, Russia, Poland, Mexico, Italy, Israel, Iceland, Czech Republic, South Africa, USA, Canada, Turkey	0.25	3.53
Club5	Hungary, Ireland, Lithuania, Latvia, Slovenia	1.49	4.82
CLUB1+2	10	0.23	4.42
CLUB2+3	-	-0.17	-3.37
CLUB3+4	26	0.003	0.051
CLUB4+5	-	-0.67	-5.74

Note: The null hypothesis of convergence is rejected at the 5% level if tb < 1.65

After the integration of the clusters, it would be revealed that among the BRICS members, China is converging with Denmark, Switzerland, New Zealand, South Korea, United Kingdom, Australia and Germany in the first club. The coefficients also show that the speed of convergence among China, Denmark and Switzerland is significant and there is a relatively strong convergence among these countries.

The other BRICS countries, namely India, Brazil, Russia and South Africa, are included in one cluster, which indicates the convergence of these countries with each other in their own group. Also, these countries have been able to form a convergence group with some of the OECD members including Greece, France, Finland, Spain, Colombia, Chile, Slovakia, Portugal, the Netherlands, Luxembourg, Japan, Estonia, Belgium, Austria, Poland, Mexico, Italy, Israel, Iceland, Czech Republic and the United States. Among the OECD members, only Lithuania, Latvia, Ireland, Hungary and Slovenia are in the third club. Having the lowest level of Bank credit assigned to the private sector, and unlike other OECD countries, they have experienced a downward trend in this index in recent years; so they won’t be able to integrate with other countries under investigation.

In the discussion of bank credit to the private sector, the limited number of clusters, and the location of countries in three broad clusters indicate the similarity of the banking sector of many of them to each other. The global average of bank credits to the private sector in 2020 was 58.91 percent, showing that all first and second club countries have an index higher than the global average. When this index is about 70% of GDP and more, the number indicates that the country has a developed financial system.

Domestic credit assigned to private sector in China with an annual growth rate of 2.89% has changed from 111% in 2000 to 182.4% in 2020. Evolution of China’s banking system and the use of Internet-based banking and investment has been one of the reasons for this significant growth, which has put it in a same cluster with developed countries, all of which are among the top ten countries in terms of world rankings. This growing trend can be observed in other BRICS countries either in a way that according to the World Bank’s Development Indicators, the Bank credit to the private sector for Brazil with a growth of 12% over the previous year, reached 70.19% in 2020. This index for India has reached 55.25 percent in the same year with an average growth rate of 3.23 percent. For Russia it has been 59.97 percent, while in 2000 the growth rate was only 16.82 percent. In South Africa with an index of 69.26 the situation is also satisfactory. Therefore, given the significant progress of the BRICS countries over the recent years, their convergence with a large number of developed OECD countries is obvious. But contrary to the first and second club countries, for the third club countries this index has been descending during these years reaching less than 50%.

Different factors are involved in the development of a country’s banking system, the most important of which are the bank interest rate spread, the monetary independence of countries, and the elimination of the governmental control over banks. The term bank interest rate spread refers to the difference between the ratio of income received from the loan to the total loans and the interest rate paid on the deposit to the total deposit (lending rate minus deposit rate, %), which indicates the financial system efficiency. Therefore, with plotting bank interest rate spread charts, the factors most important in the development of the financial system of the member countries in each club are compared together. It shall be noted that since the data of bank interest rate spread is not available for all countries, lending rate charts are plotted for more precise investigations.

**Fig 15:**
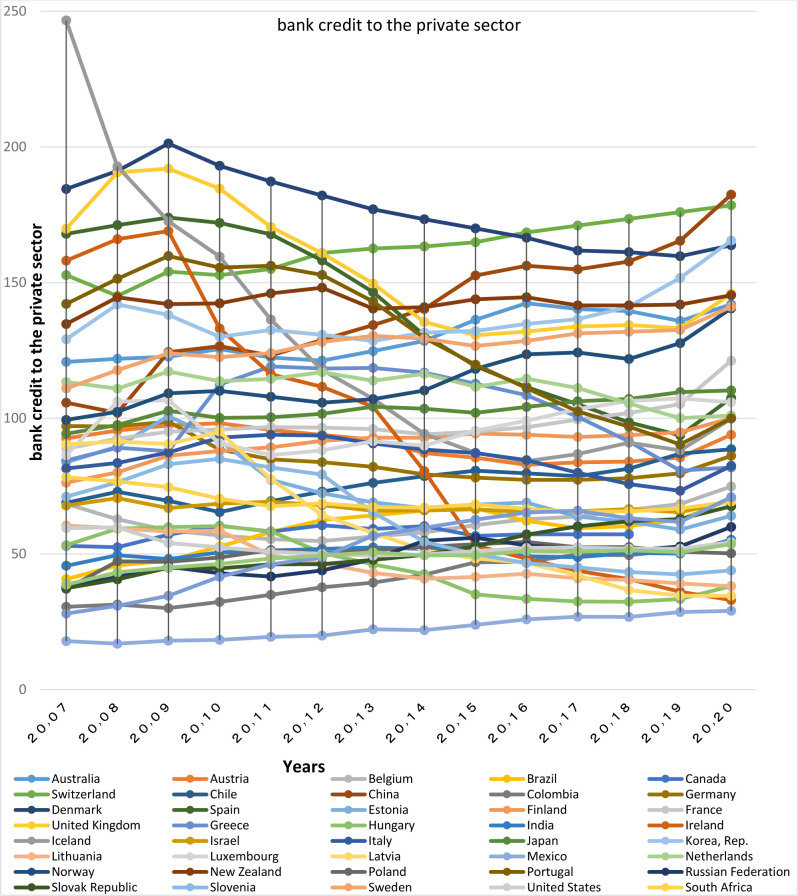
Bank credit to the private sector of BRICS and OECD countries for 2007 to 2020. The data are obtained from the World Bank. Source: World Bank.

**Fig 16:**
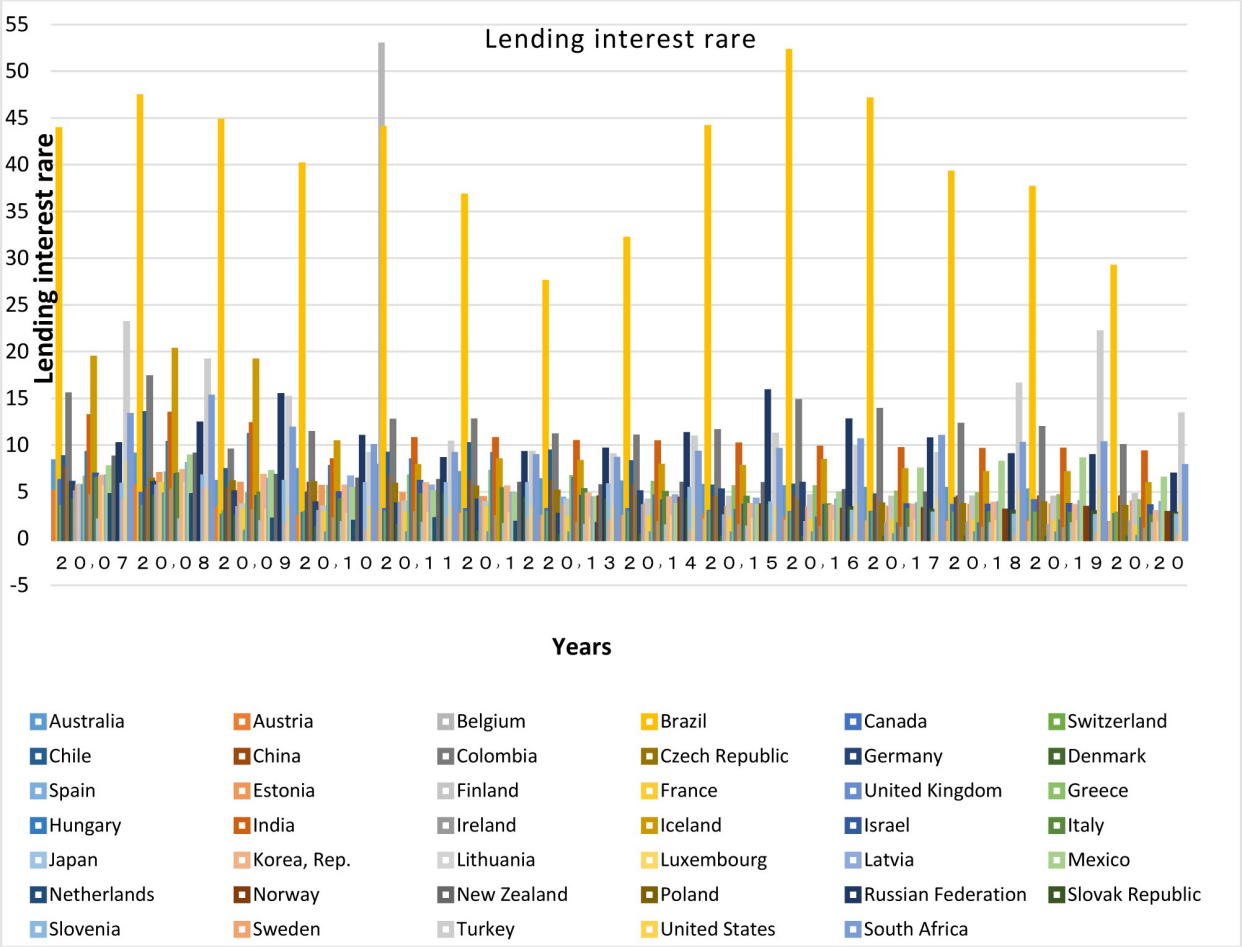
Lending interest rate of BRICS and OECD countries for 2007 to 2020. The data are obtained from the World Bank. Source: World Bank.

**Fig 17:**
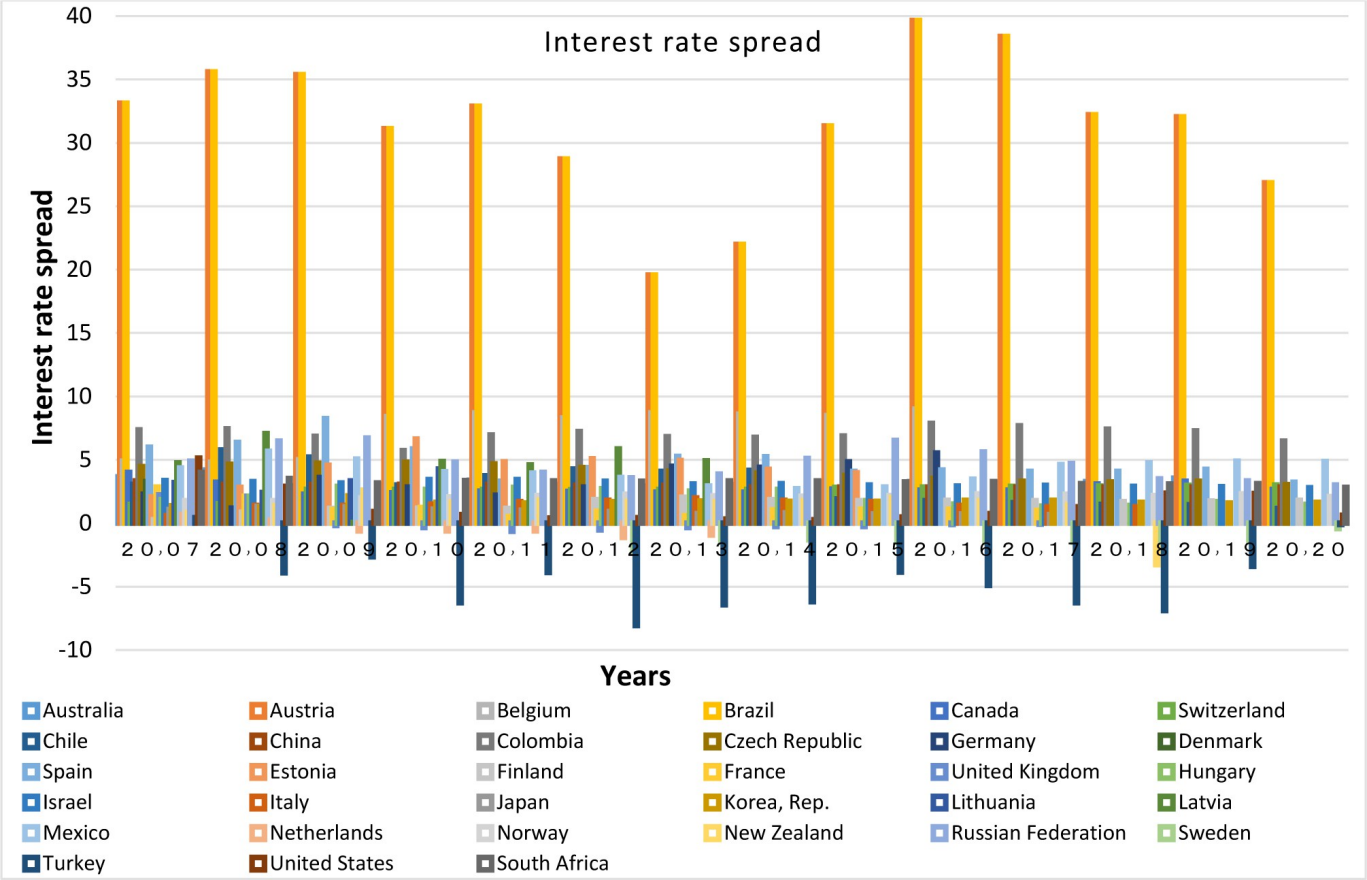
Interest rate spread of BRICS and OECD countries for 2007 to 2020. The data are obtained from the World Bank. Source: World Bank.

Based on these Figs 15–17, it’s appeared that the countries with a higher percentage of bank credit to the private sector, have bank interest rate spreads at lower levels in the chart; Therefore, it is found that the countries in the same clubs have similar Figured fluctuations in terms of bank credit to the private sector index, and their lending rates and bank interest rate spread. This shows the similarity of their infrastructure with the banking sector depth. In recent years, the bank interest rate spread in China has dropped to 2.85. Low spread and loan rates in China are strong reasons for the increase in the Bank Credit to the Private Sector index, and the convergence with other prominent countries in this regard. Loan interest rate and bank spread rate for South Africa, India, Russia and Brazil have been declining in recent years. In South Africa, the lending rate has decreased from 14.5 to 7.7 percent, and the bank interest rate spread has decreased to 2.85 percent in the last twenty years. Moreover, In India the interest rate on loans has declined from 12.08 to 9.15 percent, and in Russia it has decreased from 24.43 to 6.7%. Although these countries have attempted to keep their loan rates below the global average, Brazil, despite of significant declines in these rates, still has higher rates than other BRICS and OECD members; nevertheless, Brazil has taken important steps towards the independence of the central bank from the country’s political system; this is a clear reason for the presence of this country in the related club, and its convergence with the respective countries.

#### 5.3. Estimation of financial convergence model in the capital market

To examine the general convergence in the capital market based on the stock market capitalization index, Table 3 shows the results of the log (t) regression estimation. Given the coefficient b which is less than zero (b<0), and the t-statistic which is less than the critical value of -1.65, the null hypothesis of convergence in the whole sample is rejected at the 5% level; so due to the lack of overall convergence, the possibility of cluster convergence among the stock markets of the studied countries has been examined. According to Table 4, the results of the club convergence algorithm indicate 5 converging clubs during the period under study. The convergence test estimates done within the identified clusters show the inability of designated clubs to merge with each other, so the number of clubs yet remains 5. Among the clusters, India along with Australia, Switzerland, United Kingdom, Belgium, Canada, Czech Republic, Denmark, Finland, France, Japan, South Korea, the Netherlands, Sweden, Mexico and the United States is positioned in the first club. The stock market capitalization index in India accounted for 99% of GDP in 2020. That is, the Stock market capitalization (percent of GDP) index has grown steadily in India over the past 15 years (except for the fall of 2010), and GDP has grown in the recent years, and now this ratio has reached the highest level (almost equal to the global average level). Thus, India, due to its significant progress, has been able to be in the same convergence club with strong and well-established stock markets such as Canada (with the index of 160%), USA (194%), South Korea (126%), Switzerland (267%), Japan (133%) and the United Kingdom (131%).

**Table 3:** The result of overall convergence estimation of the stock market between the BRICS and the OECD members.

	Coefficient	T- Statistic	S.E
Logt	-1.35	-164.40	0.008

Note: The null hypothesis of convergence is rejected at the 5% level if tb < 1.65

**Table 4:** Convergence clubs classification of stock market between the BRICS and OECD countries.

Club	Countries	Coefficient	T- Statistic
Club1	Australia, Belgium, Canada, Czech Republic, Denmark, Finland, France, Japan, South Korea, Netherlands, Sweden, Switzerland, United Kingdom, India, USA, Mexico	0.125	1.33
Club2	Chile, Germany, Ireland, Israel, Italy, Luxembourg, New Zealand, Norway, Poland, Spain, Colombia, China, Brazil, Russi	0.155	3.94
Club3	Austria, Iceland, Portugal	0.45	5.88
Club4	Greece, Hungary	1.10	0.25
Club5	Estonia, Slovenia	0.80	4.94
Not convergent Group	Lithuania, Slovakia, Turkey, South Africa, Latvia	-1.58	-216.05
Club1+2	-	-1.49	-18.17
Club2+3	-	-0.11	-3.16
Club 3+4	-	-0.99	-70.55
Club 4+5	-	-1.01	-54.06
Club5+ Group6	-	-1.58	-247.1

Note: The null hypothesis of convergence is rejected at the 5% level if tb < 1.65

China, Brazil and Russia, being convergent with Chile, Germany, Ireland, Israel, Italy, Luxembourg, New Zealand, Norway, Poland, Spain and Colombia, are in the second club. China Stock Market is considered as one of the 20 strongest markets in the world, the stock market value of which has been regarded to be fair with the index of 82.96. Given the fact that the value of the stock market is about 50% of GDP and more, a developed stock market is represented; therefore, the Brazilian market, with the index of 68.41, is considered as an advanced market. Conversely, Russia, with the index of 46.83, has the lowest stock market value among the BRICS countries; though it has been growing steadily since 2014, when the stock market was experiencing the lowest value.

Austria and Portugal are in the third club, Greece and Hungary are in the fourth club, and Estonia and Slovenia are in the fifth club. Various reasons might contribute to the non- convergence of these countries with more stock markets of their group and BRICS markets. Some of the reasons include weake economic situation, small economies, and differences in political, economic and institutional aspects of countries [64].

The stock market of South Africa is different from that in other BRICS countries, because in this country the ratio of stock market capitalization to GDP with an index of 313.4 is much higher than that in other countries studied. That’s why this country has not been placed in any convergence club. High stock market value does not necessarily mean the active statue of the market. The stock market can consist of several large companies the shares of which are rarely traded. Despite its high stock market value, South Africa has a relatively small number of listed companies on the market. Other than South Africa, Lithuania, Slovakia, Turkey and Latvia are in the non-convergent group, where fluctuations and weak levels of stock market development, the susceptibility by global economic crises and restrictions on money transfers, and government interventions, etc. can be considered of its factors.

## 6. Discussion

As mentioned in the Theoretical Foundations section, the necessary condition for establishing convergence among countries is the existence of trade freedom and the trade relations between them. A sufficient condition for this issue would be the availability of similar economic infrastructure; therefore, after examining financial convergence in the money and capital markets, and identifying convergent clubs, the necessary and sufficient conditions for convergence between countries in the two financial markets are examined separately in this section.

### Money market

In order to assess the necessary condition, first of all the trade between convergent countries is studied. According to [65], [66–68], the development of trade relations and trade openness as a feature of globalization, is of the most important factors of financial market convergence.

**Fig 18:**
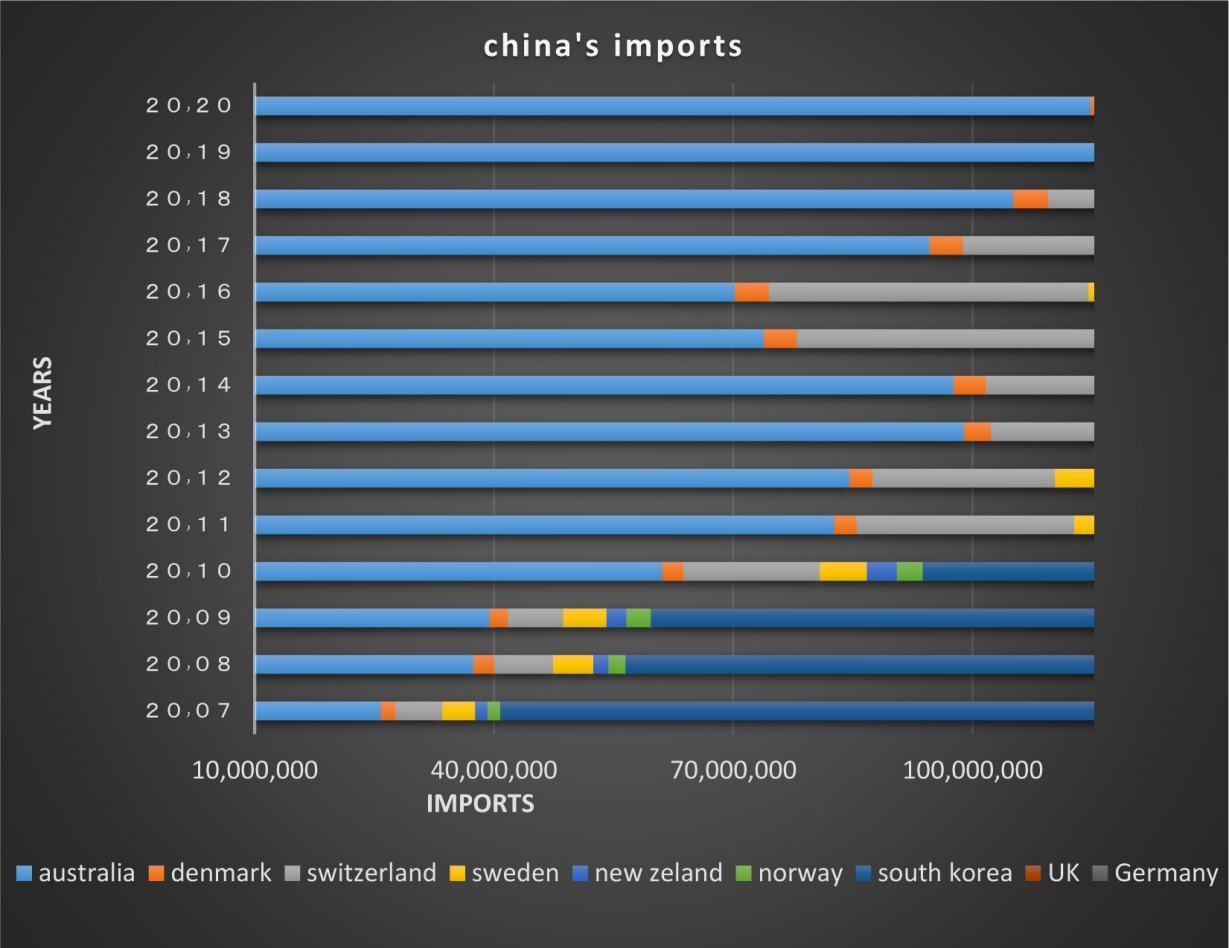
China’s imports from other member countries of the convergence club in the money market, 2007–2020. Source: Trade Map.

**Fig 19:**
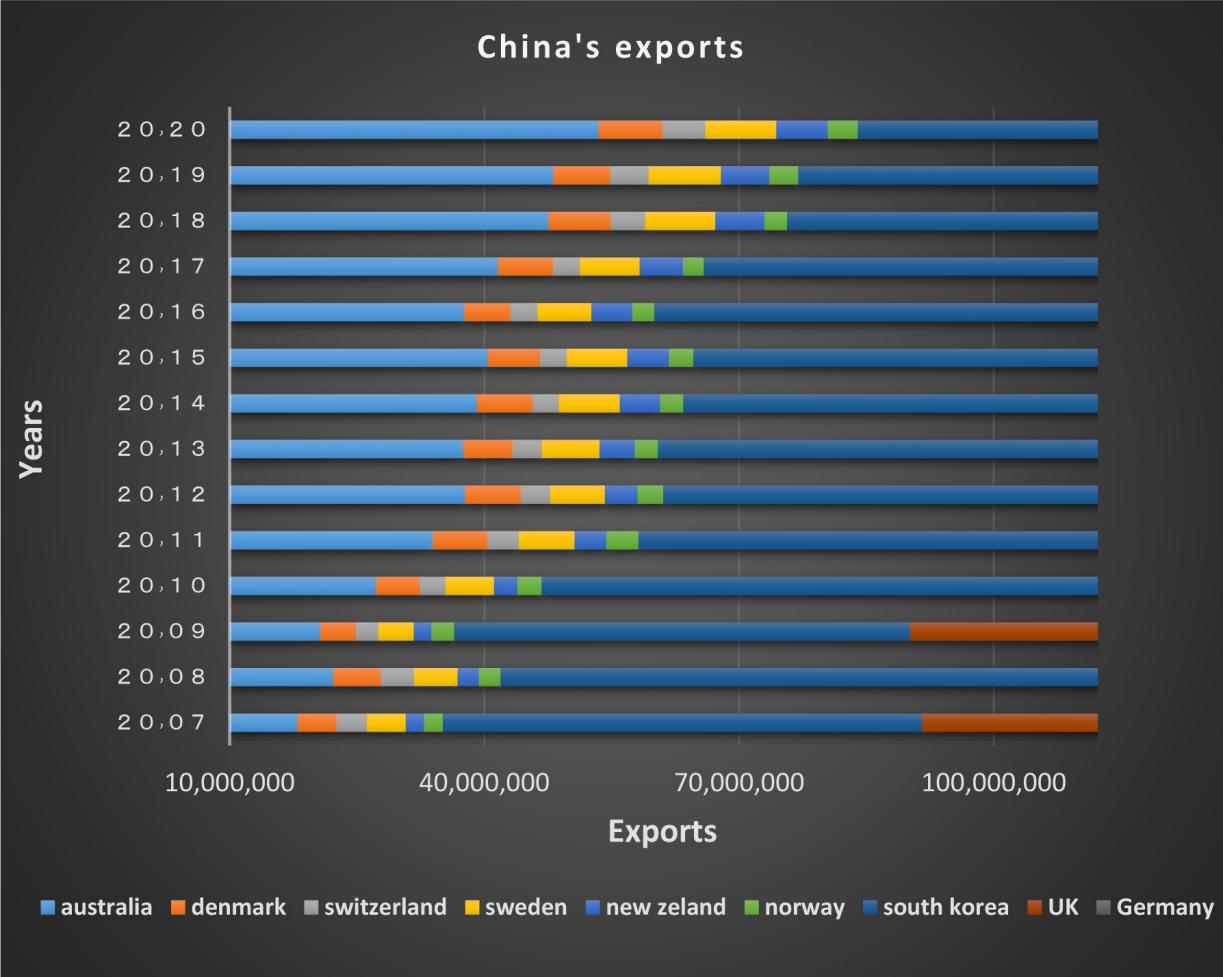
China’s exports to other member countries of the convergence club in the money market, 2007–2020. Source: Trade Map.

In the first club, as depicted from Figs 18 and 19, it’s obvious that imports and exports have been established between China and its partner countries. Most of these countries including South Korea, Germany, the United Kingdom and Australia have been among the top 15 countries in terms of having transactions with China in 2020; besides, China, South Korea, New Zealand and Australia have signed the Regional Comprehensive Economic Partnership Agreement regarding the establishment of the largest free trade area in the world. The mentioned agreement brings the rates in the financial markets of the member countries closer together, and as a result convergence is formed.

Switzerland also has extensive trade relations with China. Since 2010 China has been Switzerland’s largest trading partner in Asia and its third largest trading partner in the world after the European Union and the United States. Both countries signed the S-CFTA (Switzerland- China free trade agreement) Free Trade Agreement in 2013, which removed many trade barriers. Bilateral trade between China and Denmark peaked to 14.7 percent from 2019 to 2020. China is Denmark’s sixth largest export market. Norway is another important trading partner in northern Europe for china. China and Norway have been conducting extensive FTA studies since 2007 so as to deepen their bilateral relations and cooperation in the economy and trade. Trades and transactions between them has increased significantly during the recent years.

**Fig 20:**
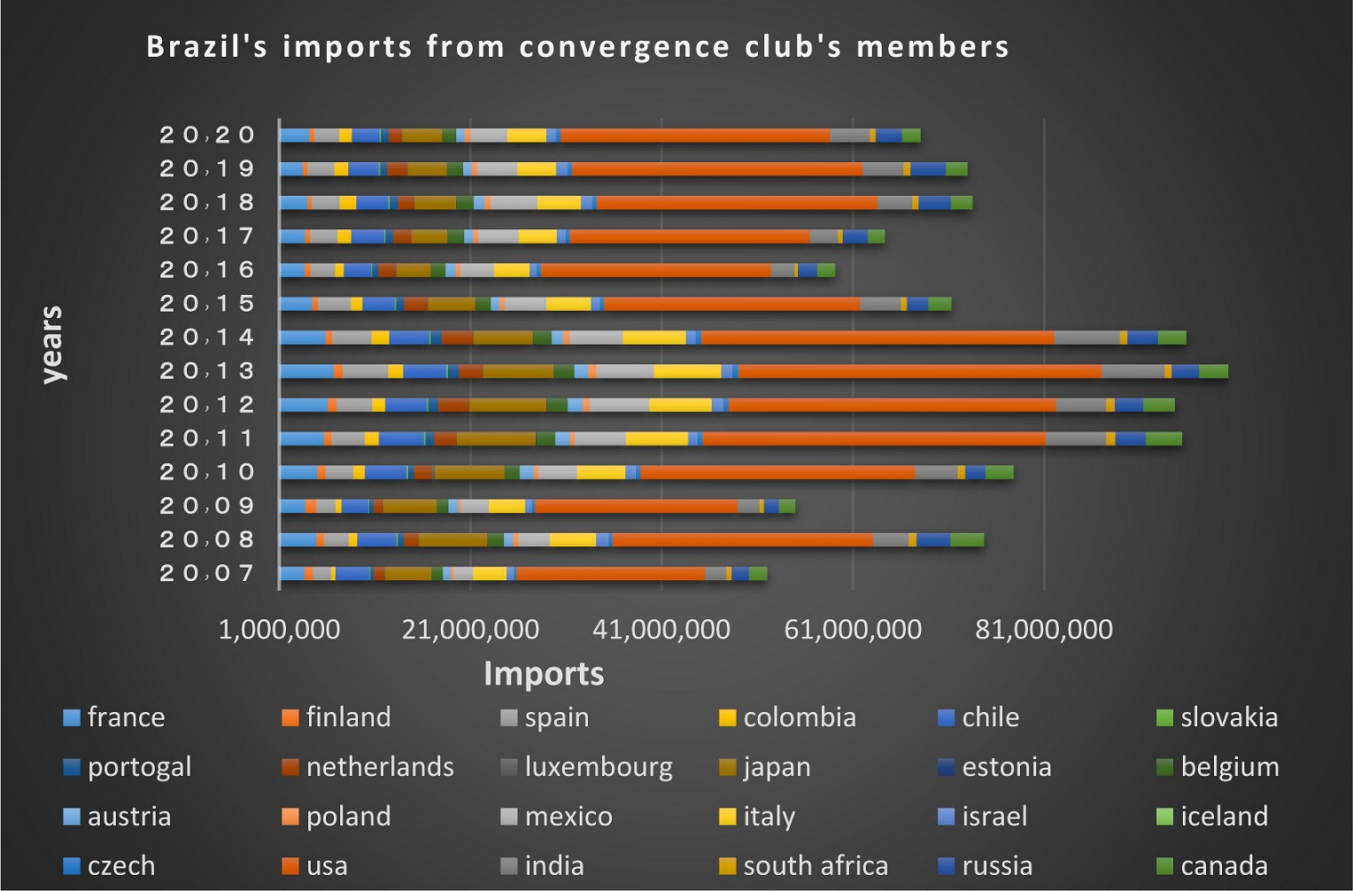
Brazil’s imports from other member countries of the convergence club in the money market, from 2007–2020. Source: Trade Map.

**Fig 21:**
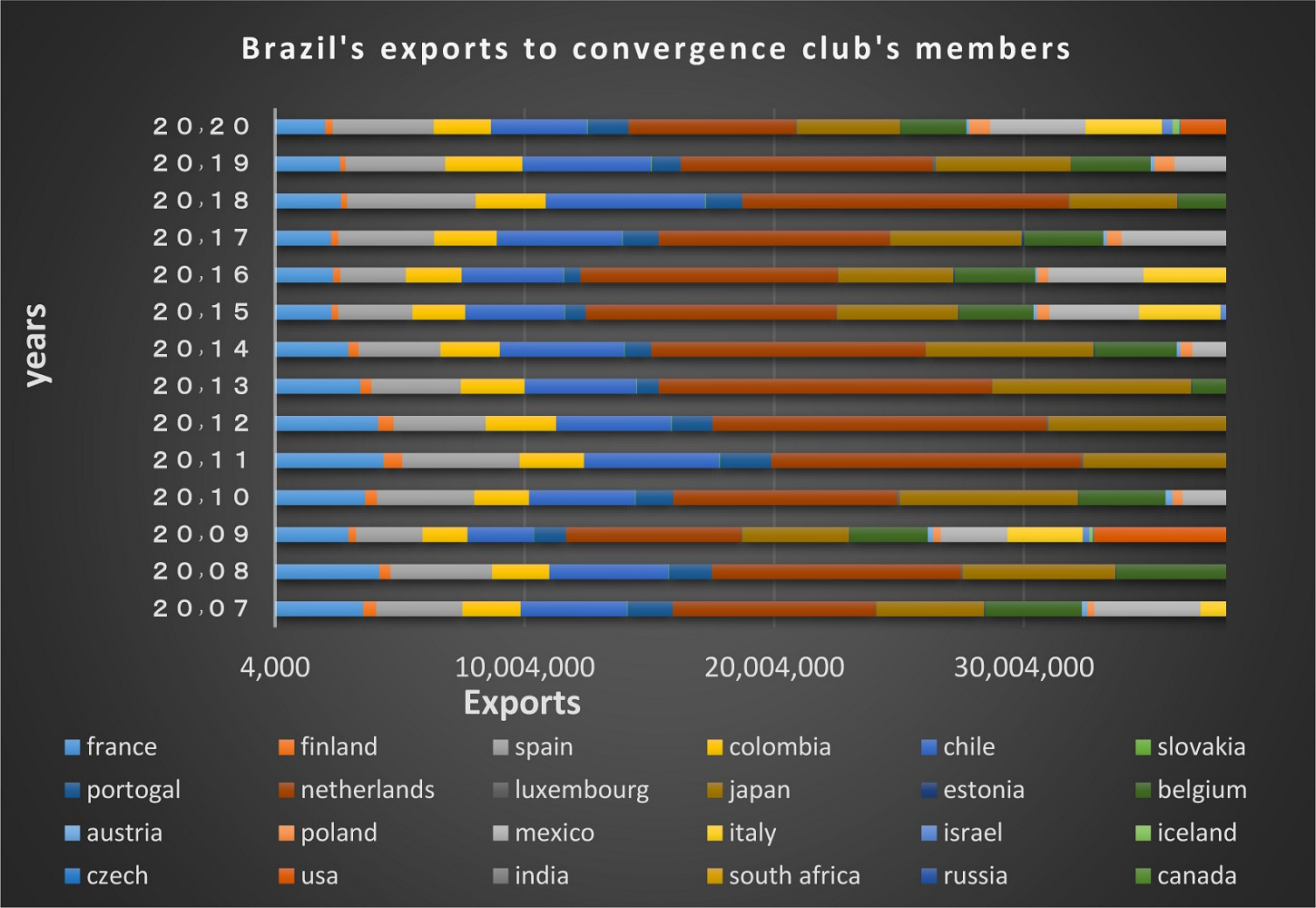
Brazil’s exports to other member countries of the convergence club in the money market, from 2007–2020. Source: Trade Map.

Figs 20 and 21 show the significant trade volume of Brazil with countries converging with that in the second club. Many EU countries in this club that are of Brazil’s main trading partners. The free trade agreement already made between the EU and Mercosur (Southern Common Market) is important reason for Brazil’s convergence with these countries, thereby many trade restrictions in these two groups of countries have been eliminated resulting in the boost of trades between them. Among the members of this club, the Netherlands, Canada, Japan, Spain, Chile, Mexico, Italy and India are the countries that have imported the most shipments of Brazil in dollar value in 2020. The United States, India, Japan, Italy and France are Brazil’s main partners. The Latin American countries are also in a joint club with Brazil, and there are trading relations between them based on the charts.

**Fig 22:**
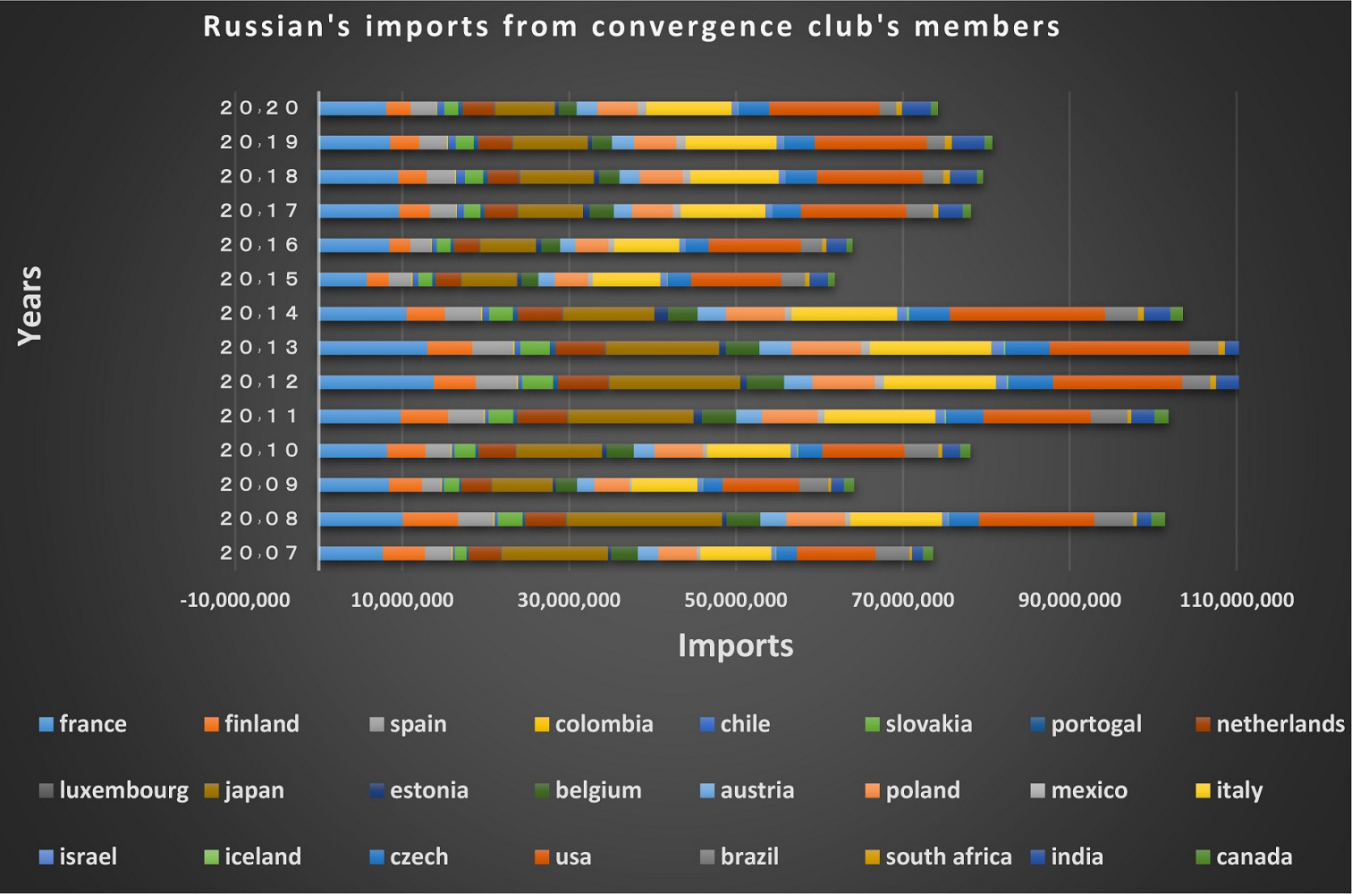
Russian Federation’s imports from other member countries of the convergence club in the money market, from 2007–2020. Source: Trade Map.

**Fig 23:**
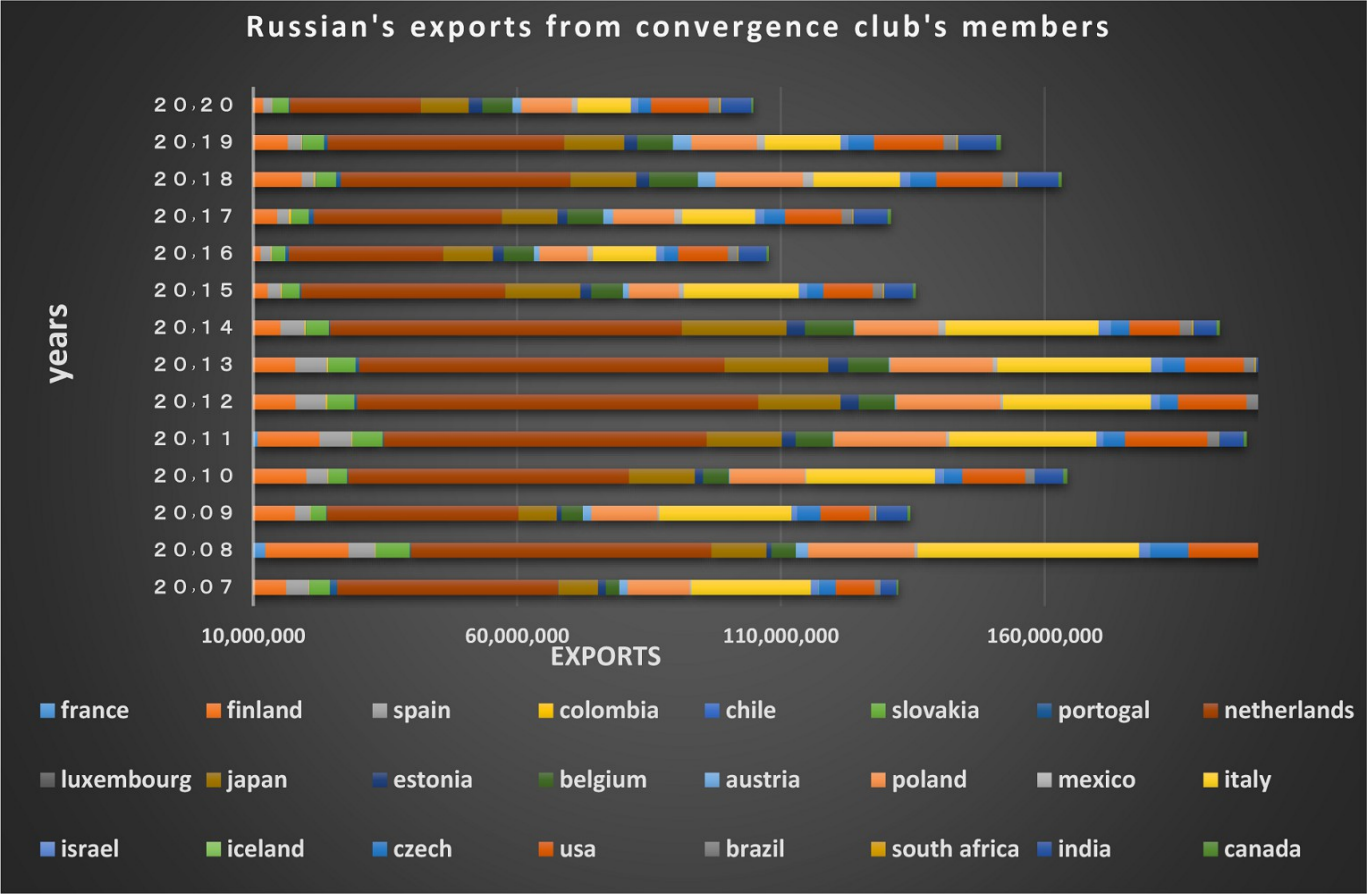
Russian Federation’s exports to other member countries of the convergence club in the money market, from 2007–2020. Source: Trade Map.

Figs 22 and 23 show the trade relations of the countries with Russia in the mentioned club. Most of the European countries in the club are Russia’s top trading partners in 2020 (i.e. France, Finland, the Netherlands, Poland, Belgium, and Italy). According to the charts, the positive trading trend between Russia and other countries in the same group is confirmed during the period under review.

**Fig 24:**
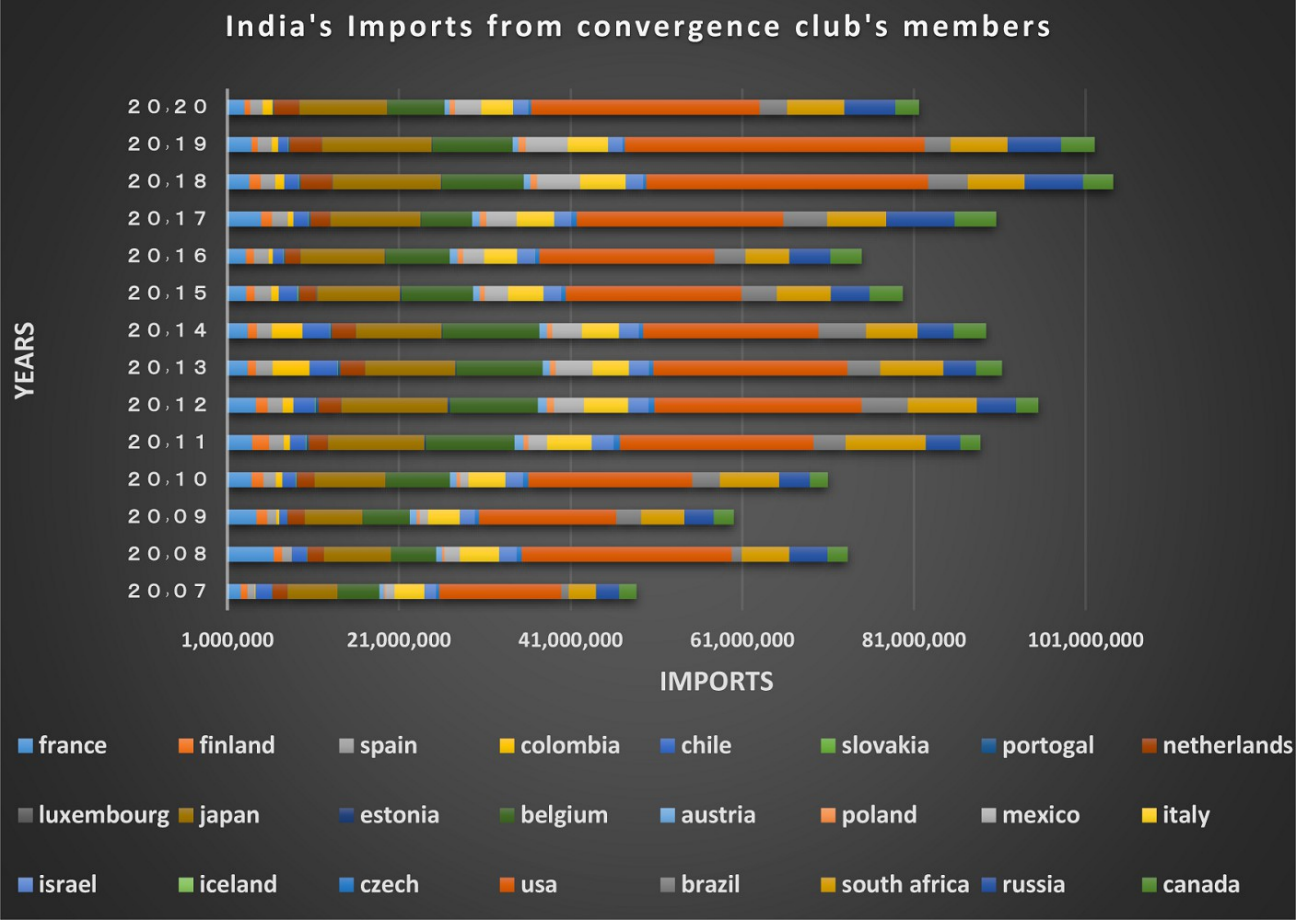
India’s imports from other countries in the convergence club in the money market, from 2007–2020. Source: Trade Map.

**Fig 25:**
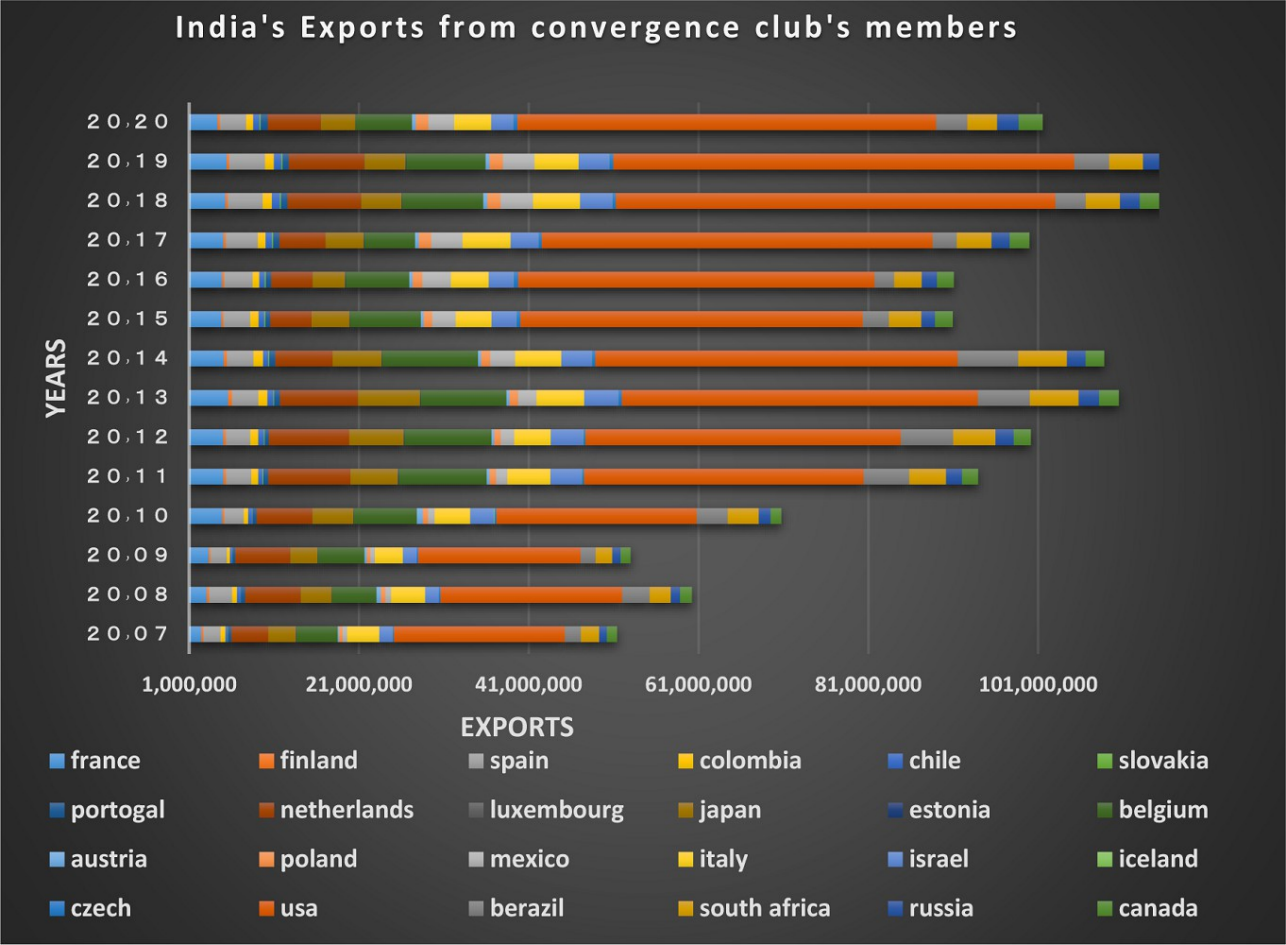
India’s exports to other countries in the convergence club in the money market, from 2007–2020. Source: Trade Map.

Figs 24 and 25 show the trade relations of India with the countries of the related cluster. From the Figs it can be seen that exports and imports have been established continuously between these countries during the period under study, and the volume of trade between them is also significant.

**Fig 26:**
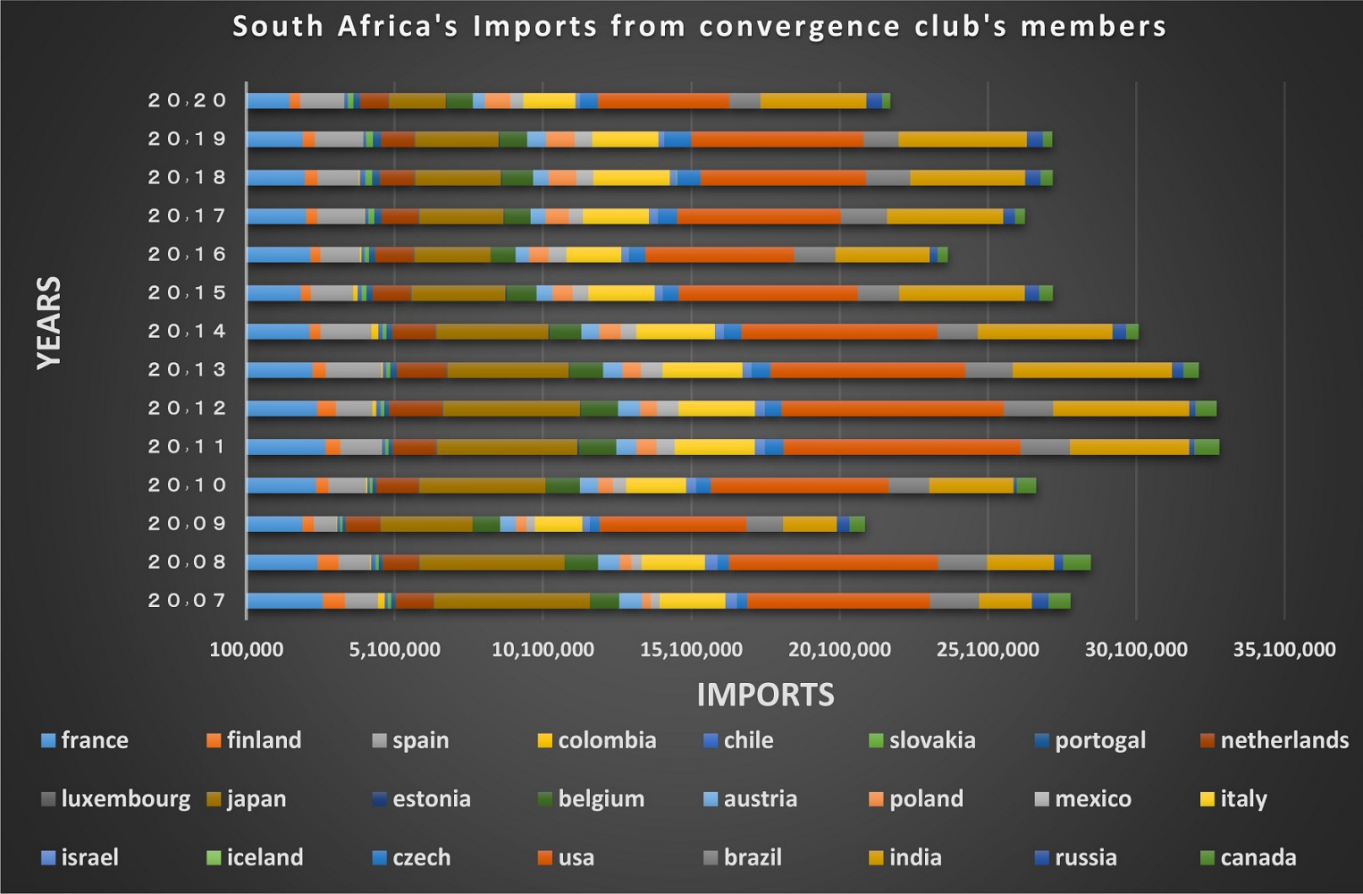
South Africa’s imports from other countries in the convergence club in the money market, from 2007–2020. Source: Trade Map.

**Fig 27:**
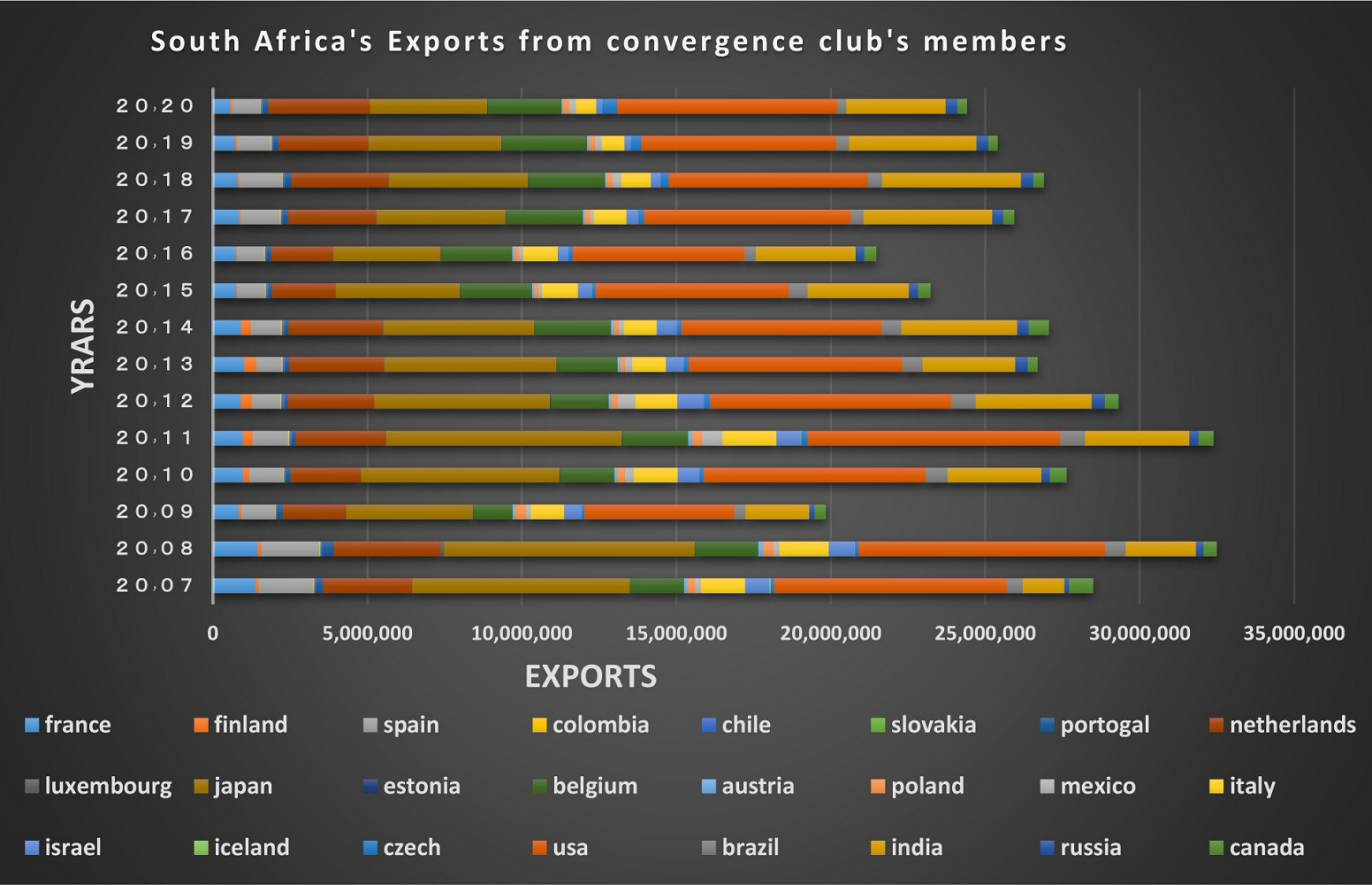
South Africa’s exports to other countries in the convergence club in the money market, from 2007–2020. Source: Trade Map.

South Africa exported commodities worth of about $ 86.1 billion worldwide in 2020, reflecting an increase of 12.45% from 2016. This shows that Africa in recent years has taken important steps towards trade freedom. The European Union, the United States, India, Japan and Mexico are the main trading partners of South Africa, which are in a joint club with the country. South Africa is an important country for the European Union and its exports to other countries particularly to the Netherlands, Belgium, Spain and Italy cannot be ignored (See Figs 26 and 27).

The financial and trade freedom of the BRICS countries is investigated in the following as another necessary condition for converging with OECD countries. Freedom indexes play a key role in creating convergence. According to the Trade Freedom Fig 2, it is obvious that all BRICS countries, to form a convergence club with OECD developed countries, have first brought their level of trade freedom to the global average. Based on the financial freedom charts of these countries, this index has reached the global average only in Brazil, South Africa, and China. India and Russia yet have a long way to the financial freedom. The weak economy of these countries in financial freedom might be one of the reasons of such slow pace.

After examining the necessary condition, a brief description of the economic structure of the BRICS countries is provided below. Then the degree of similarity of their economic structure with the OECD countries in the same group is examined as a sufficient condition. For this purpose, Figs of the variables of GDP growth, inflation rate, real interest rate and money supply are plotted and assessed for each club members.

GDP growth is the most important variable that describes a country’s economic strength. The development of an economy requires low inflation. So when the government succeeds in keeping inflation low, the upward trend in growth is commenced.

**Fig 28:**
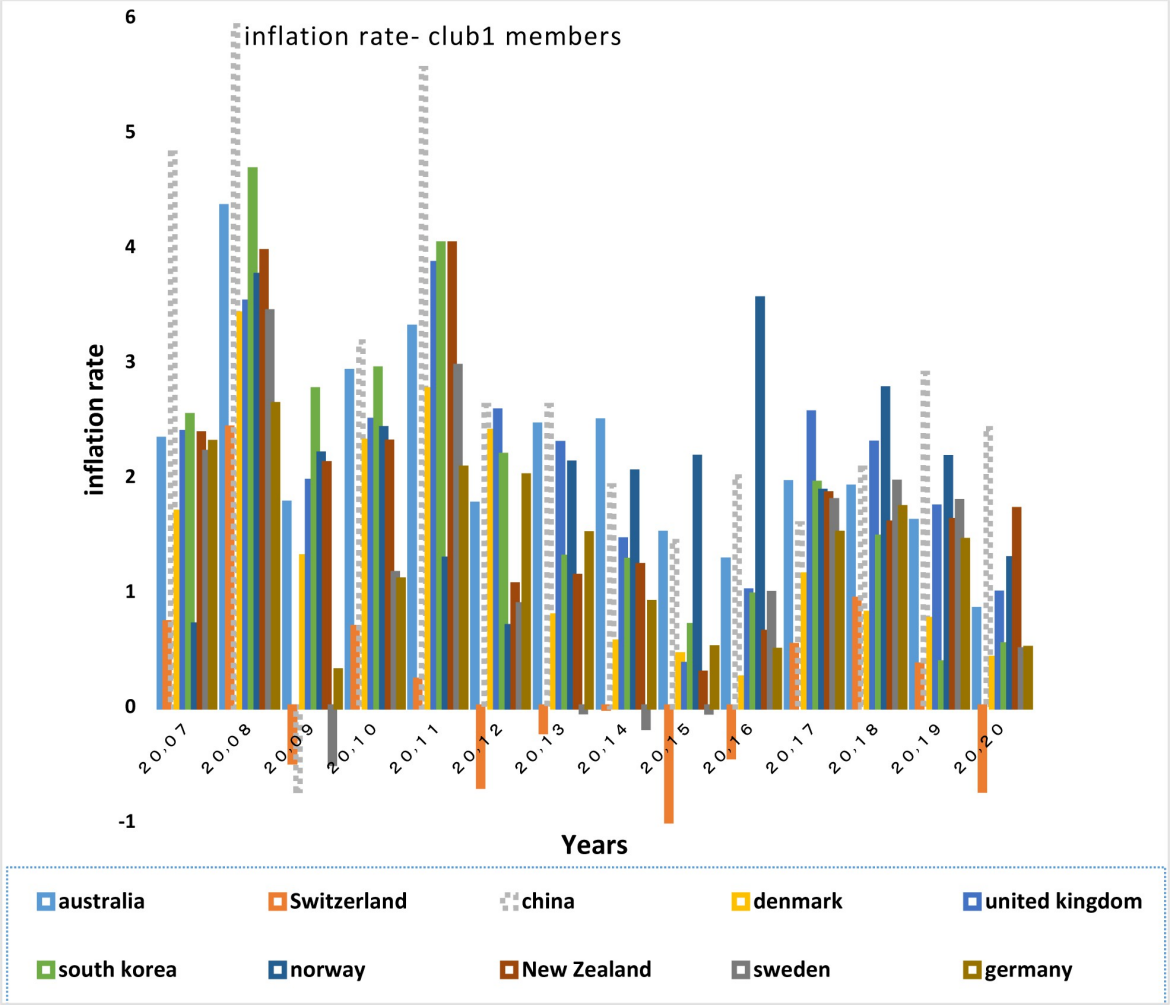
Inflation rate- club1 members in the money market, from 2007 to 2020. The data are obtained from the World Bank. Sours: world Bank.

**Fig 29:**
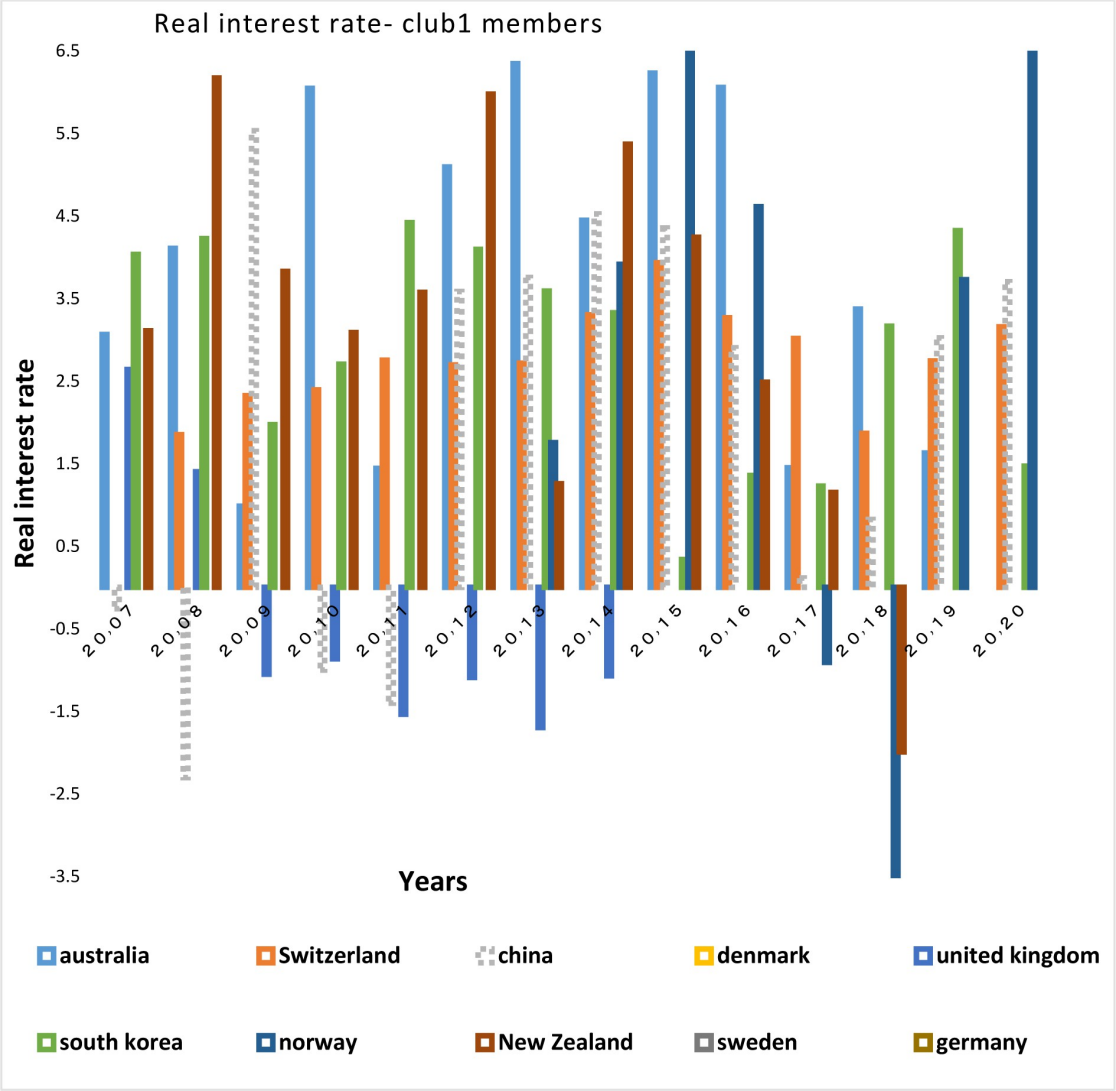
Real interest rate- club1 members in the money market, from 2007 to 2020. The data are obtained from the World Bank. Sours: world Bank.

**Fig 30:**
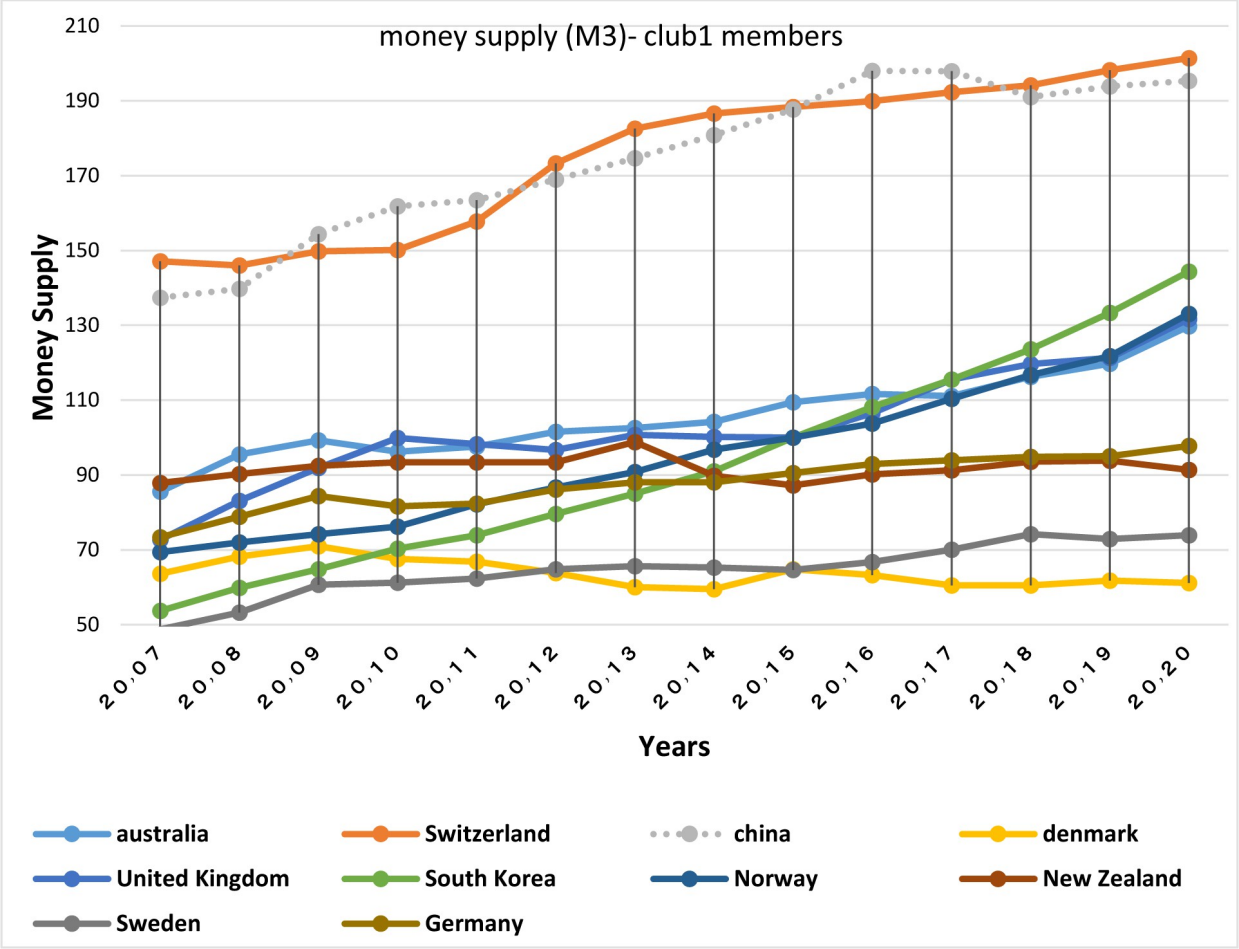
Money supply (% of GDP) (M3)- club1 members in the money market, from 2007 to 2020. The data are obtained from the International Monetary Fund (IMF). Sours: world Bank.

Figs 28 and 29 show inflation rates and interest rates. According to the Figs, the dispersion between countries’ rates is high. Although the similarity is less in their interest rate fluctuations, during the period, interest and inflation rates of these countries have become closer to each other. Fig 30 is associated with the money supply (M3) of the first club countries. It shows that due to the difference between countries in terms of economy and population size, these countries are far apart in terms of money supply. The chart shows that the changes of their money supply during these years have been similar and consistent. Another important point in examining the inflation and money supply charts of this group is that in most of the countries of this club that have high money supply i.e. China and Switzerland, a controlled inflation has been reported, which confirms the efficient financial system of these countries. In fact, although the charts of countries member in this club show a high distance, their changes rhythms are quite similar.

In addition to the similar economic structures and trade relations between countries, interactions among them in other non-economic areas strengthens the relationship between them. the extensive China-Switzerland relations in terms of cultural and artistic fields, the promotion of human rights, and China’s comprehensive strategic partnership with Denmark since 2008, which has increased the bilateral relations between the two countries and has led their collaborations in other areas i.e. research, innovation and education, energy, health, public welfare and the environment, and trade relations are of such interactions. All of the above-mentioned items have a significant effect on the convergence among the countries, in a way that according to Table 2, a greater financial convergence coefficient between the countries has been made. The BRICS countries in the second club have each experienced many fluctuations in GDP growth and inflation.

**Fig 31:**
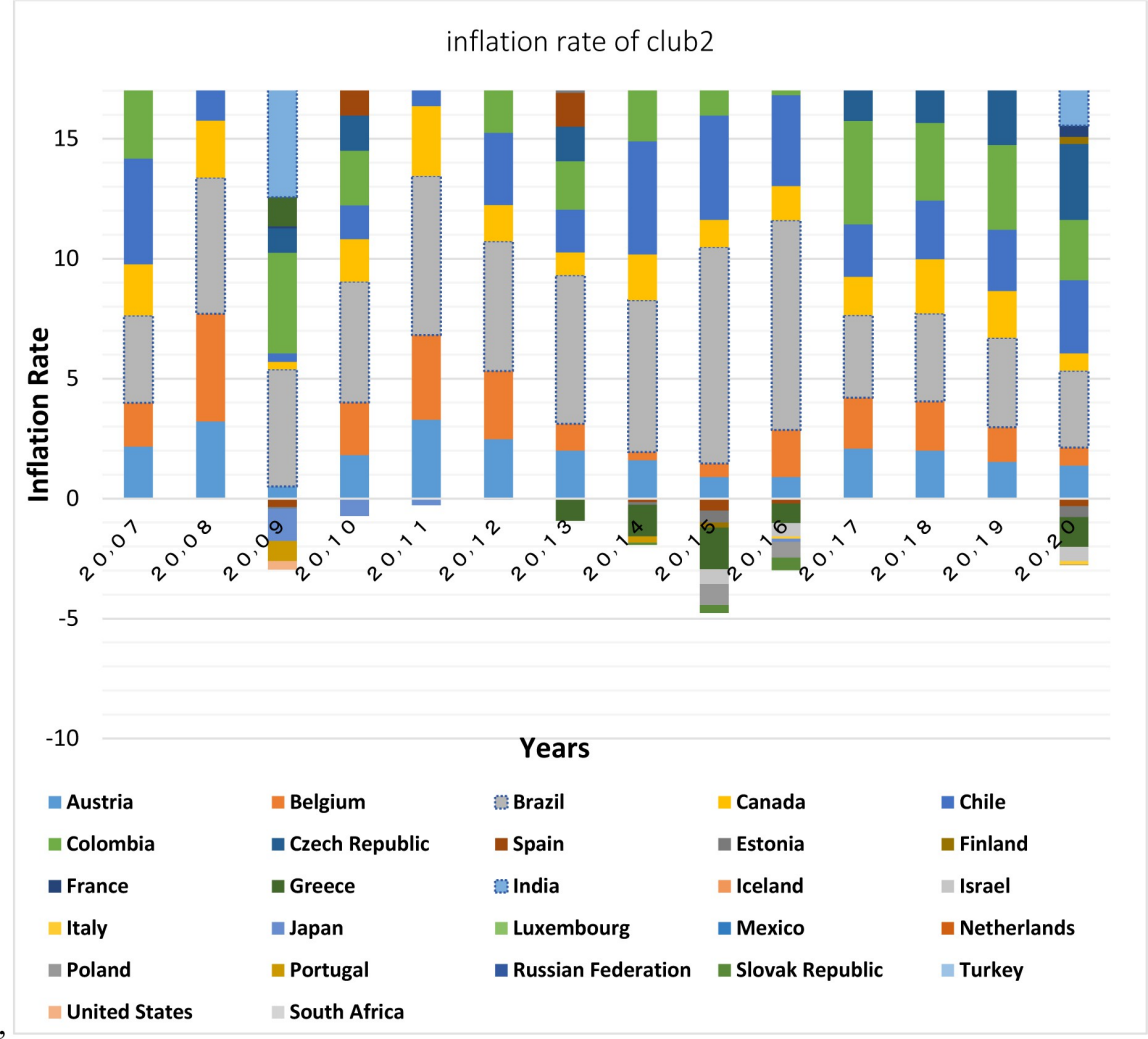
Inflation rate- club2 members in the money market, from 2007 to 2020. The data are obtained from the World Bank. Sours: world Bank.

**Fig 32:**
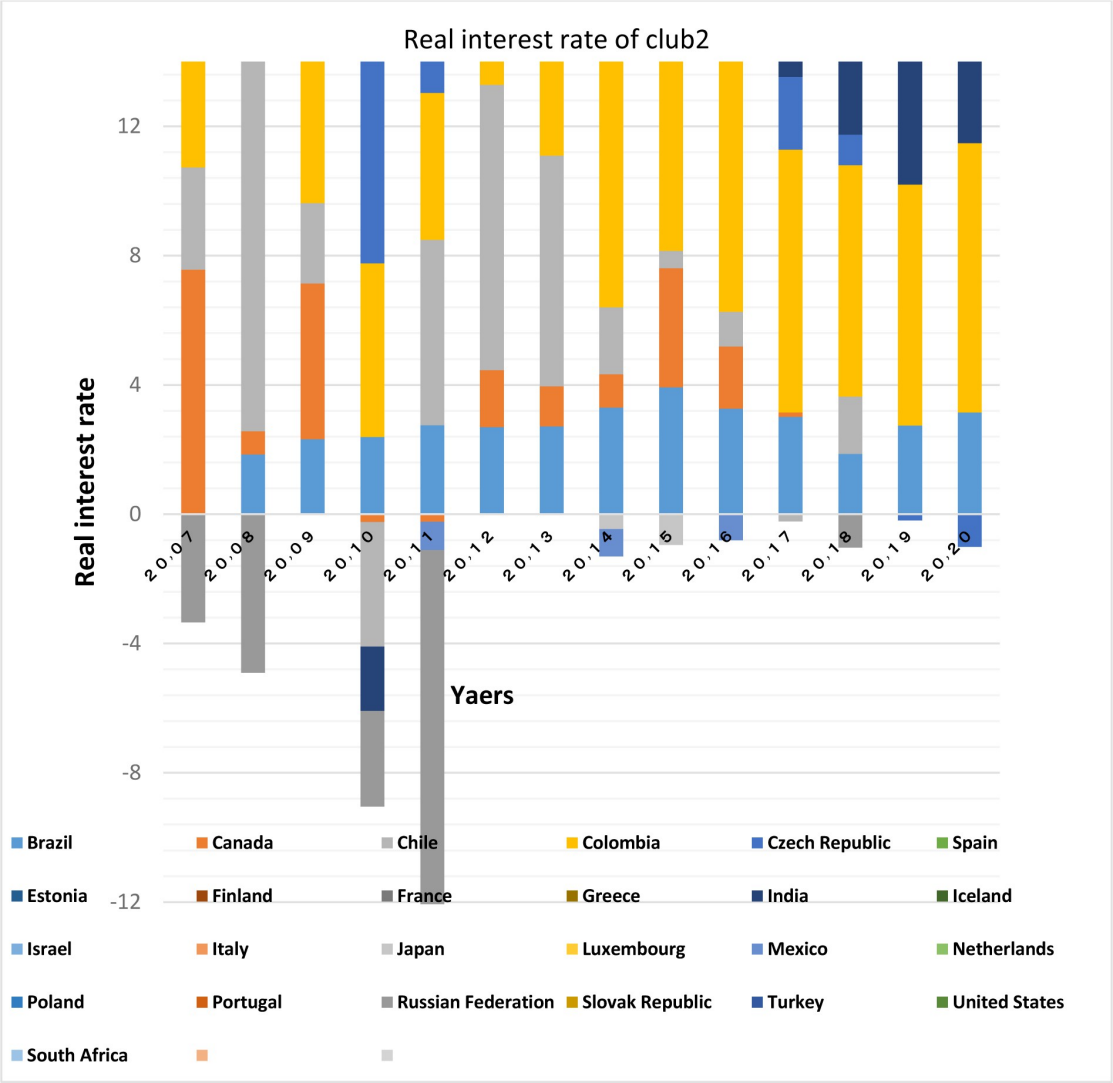
Real interest rate- club2 members in the money market, from 2007 to 2020. The data are obtained from the World Bank. Sours: world Bank.

**Fig 33:**
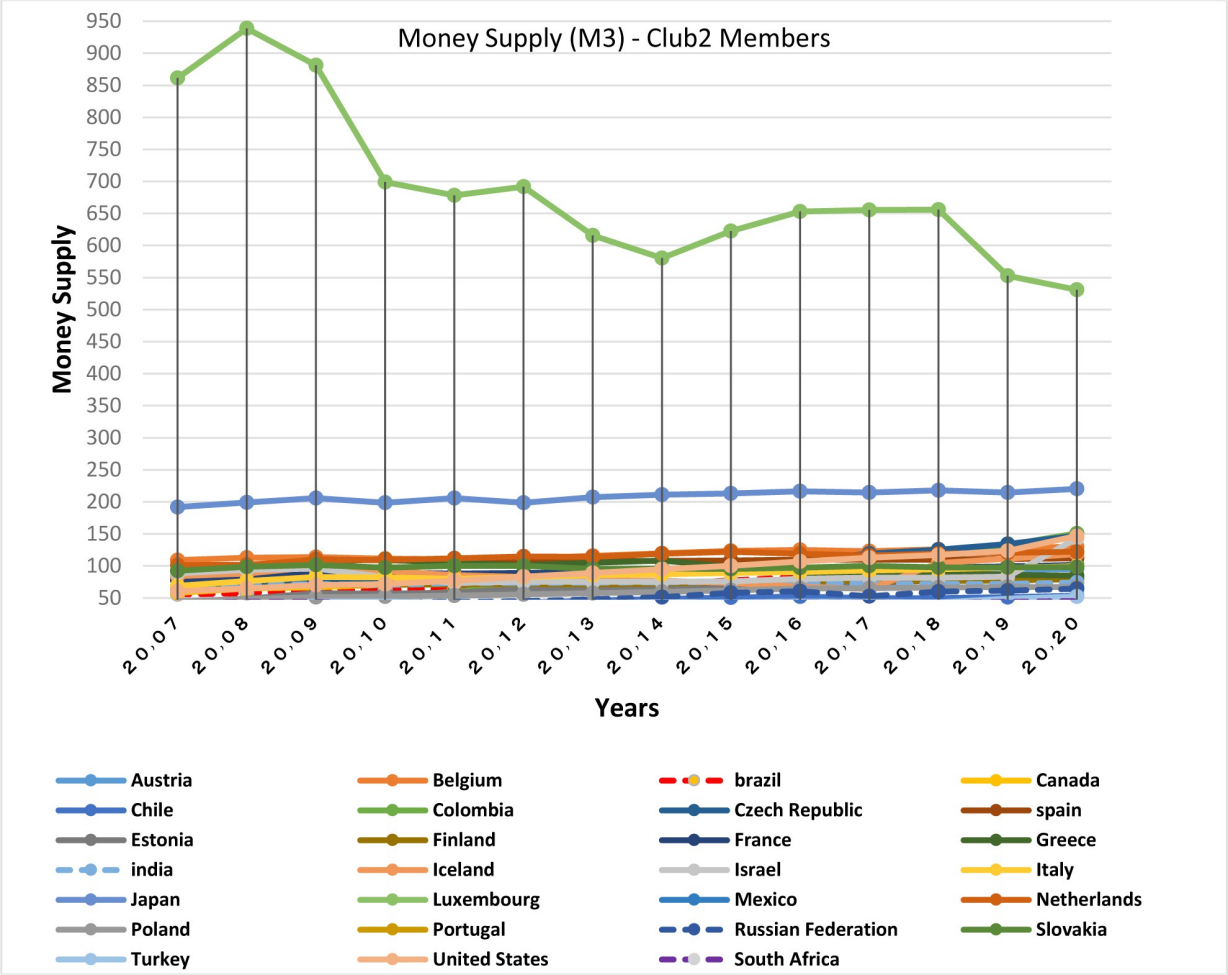
Money supply (% of GDP) (M3)- club2 members in the money market, from 2007 to 2020. The data are obtained from the International Monetary Fund (IMF). Sours: world Bank.

Figs 11 and 12 show that the countries member in the second convergence club have same behaviors in terms of GDP growth. Over time, their fluctuation has decreased and they have become closer to each other. Fig 31 shows the inflation rate of the members of this club. Inflation rate in emerging BRICS countries has been higher and more fluctuating than that in the OECD countries until the middle of the period, but in the recent years, due to development and evolutions in globalization and improvement of macroeconomic policies making, inflation rate has decreased getting closer to other developed countries of their club. Fig 32 shows the interest rates of the countries of the second club. Interest rates are of the most important tools of monetary policy for controlling inflation. Most developed countries with strong monetary policies use interest rates to curb inflation, but statistics show that in recent years emerging BRICS countries have raised their interest rates to reduce inflation. The implementation of similar policies between countries indicates the closeness of their economic structure. Fig 33 is about money supply. According to this chart, the growth trend of money supply (M3) in the member countries of the second club is similar and upward. Since the emerging BRICS countries in this club like the developed OECD countries have a downward trend in inflation as the money supply increases, it can be acknowledged that the policies used by financial institutions have been correct, efficient and with no side effects. This indicates the development of the financial system of these countries over time.

In terms of the growth and development of the BRICS emerging countries in recent years, it would be enough to state that until 2014, Brazil, India and Russia were in the category of emerging economies; but in 2015, India became the sixth largest economy in the world and Russia replaced Canada in this list. In recent years China, India and Brazil have all been among the top 10 economies in the world; therefore, it can be said that the BRICS countries have made significant progresses in their economic structure, which has led to their convergence with developed and mostly powerful countries.

Regarding the conditions of convergence among Hungary, Ireland, Lithuania, Latvia, and Slovenia in the third club, it can be acknowledged that trade relations among these countries have always been established and these countries are almost each other’s top trading partners. They also have similar economic structures, development status and geographical proximity. Though, differences in industrialization size compared to other countries under study have prevented them from converging with the two other clubs.

### Capital market

**Fig 34:**
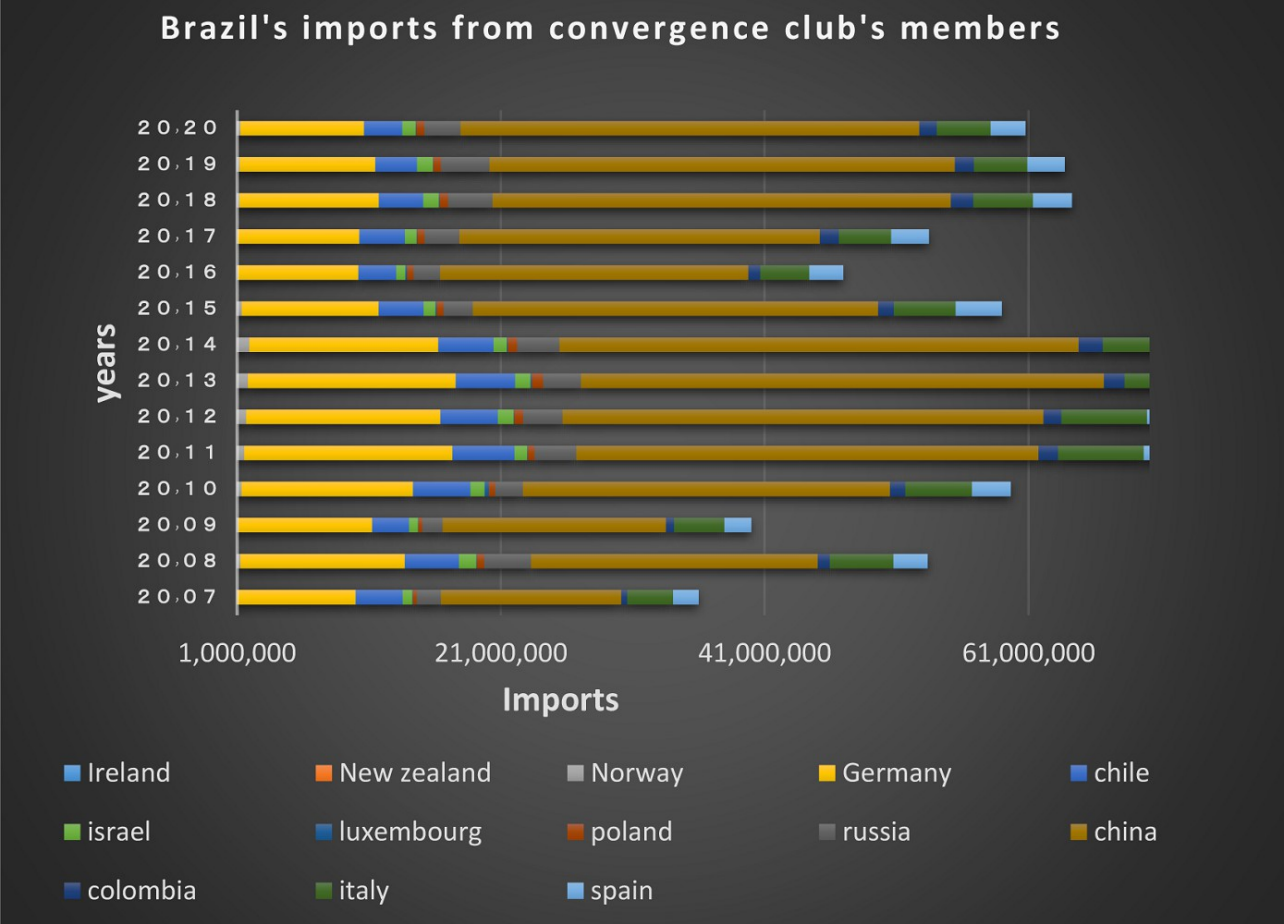
Brazil’s imports from other member countries of the convergence club in the capital market, from 2007–2020. Source: Trade Map.

**Fig 35:**
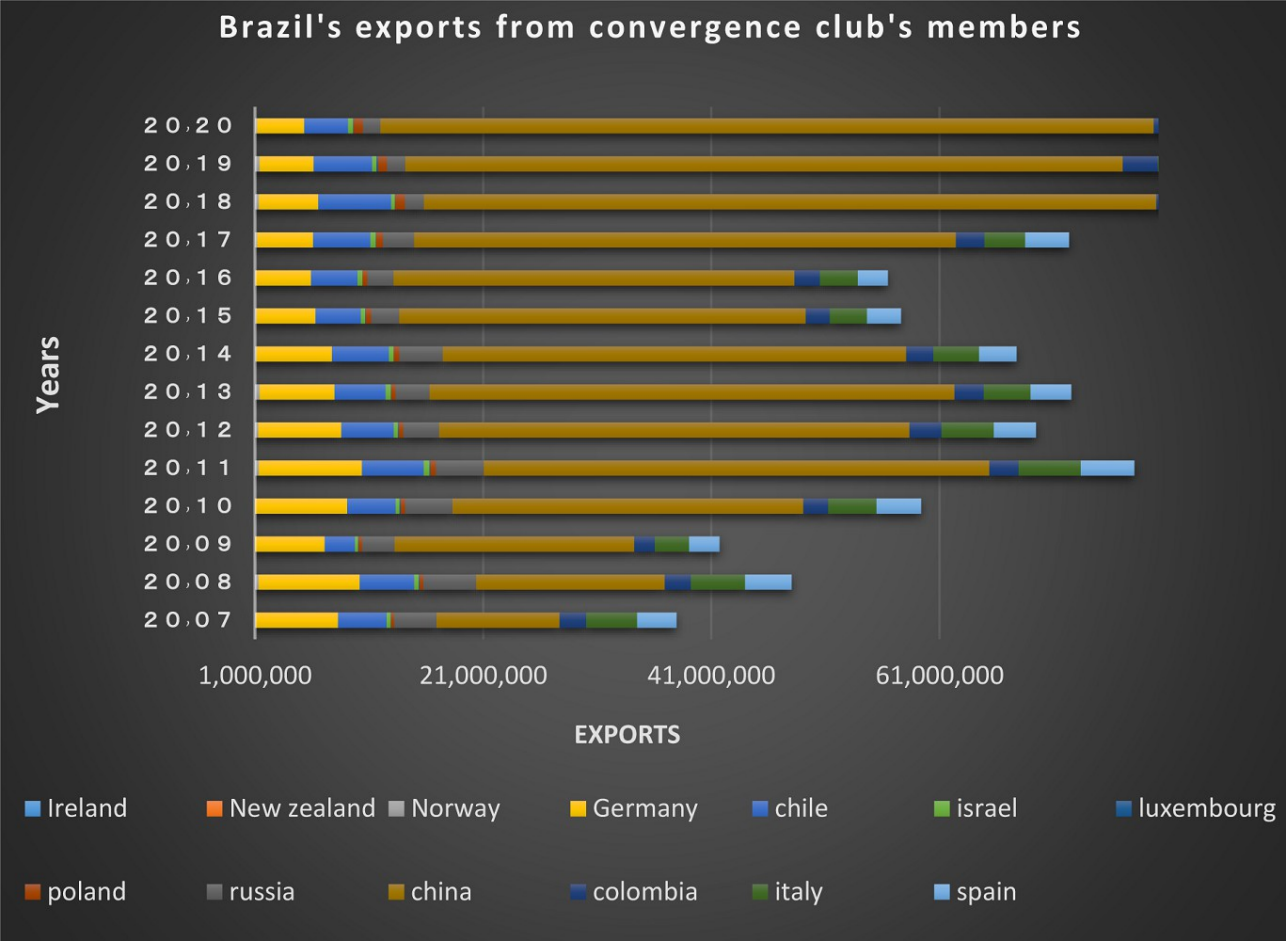
Brazil’s exports to other member countries of the convergence club in the capital market, from 2007–2020. Source: Trade Map.

**Fig 36:**
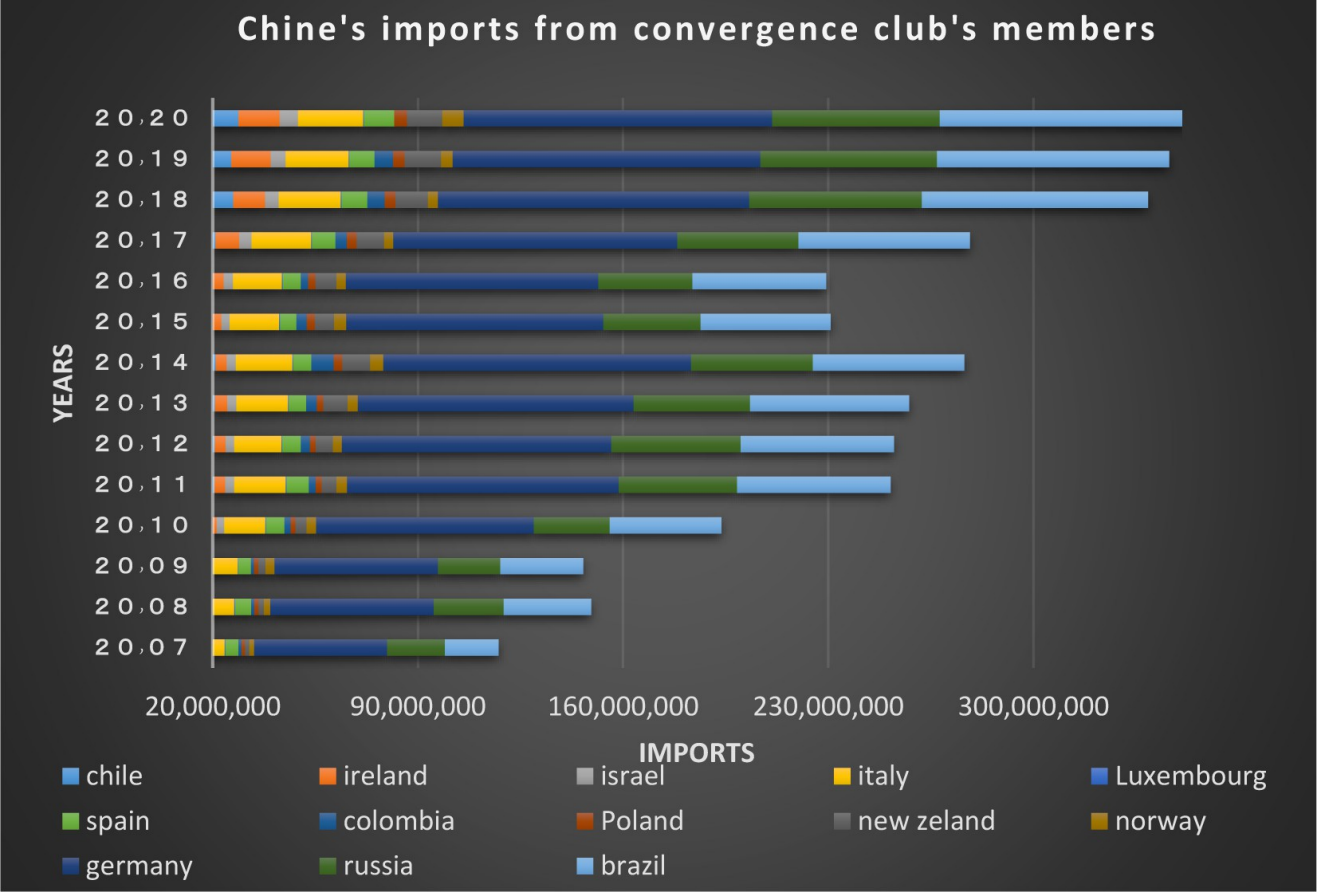
China’s imports from other member countries of the convergence club in the capital market, 2007–2020. Source: Trade Map.

**Fig 37:**
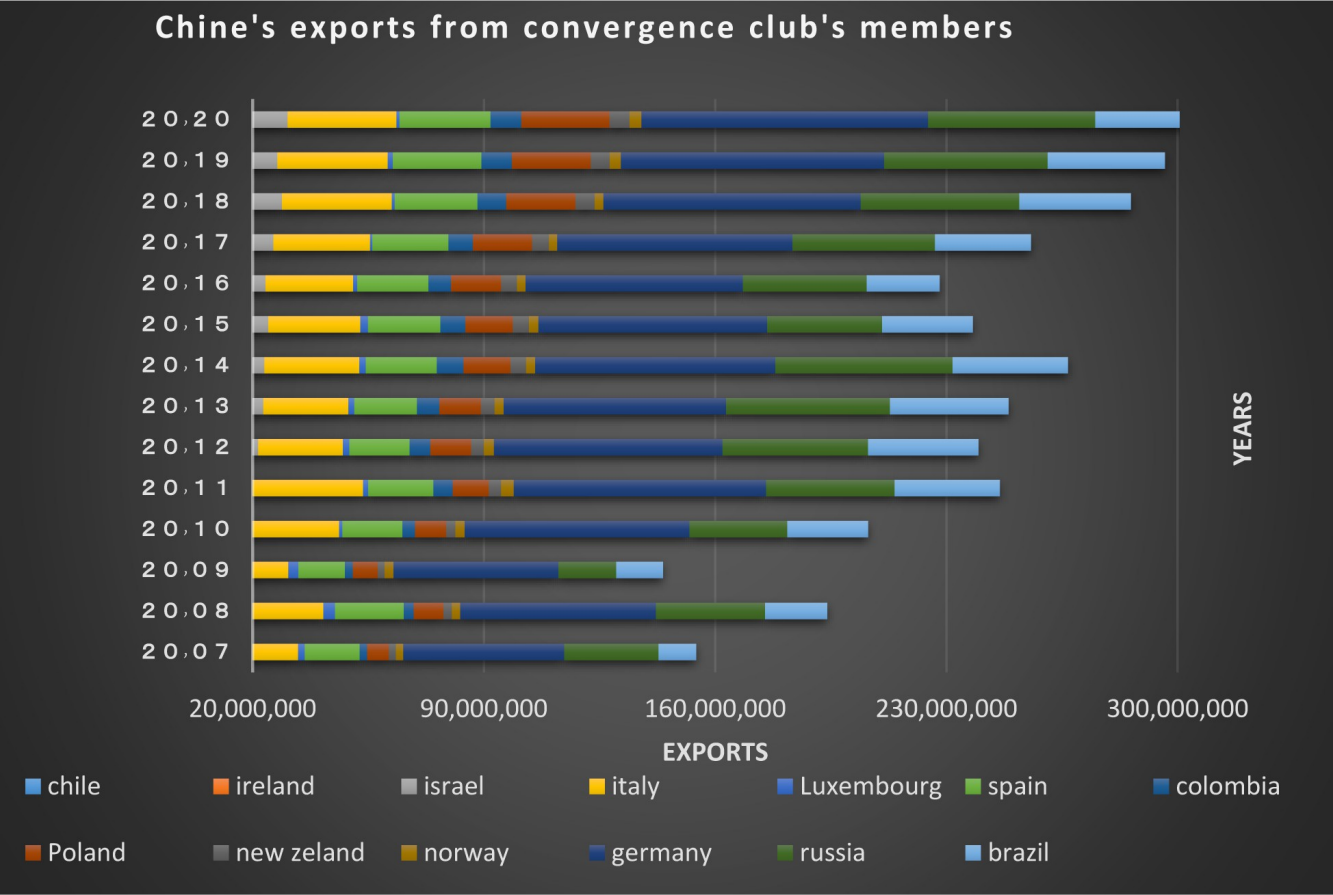
China’s exports to other member countries of the convergence club in the capital market, 2007–2020. Source: Trade Map.

**Fig 38:**
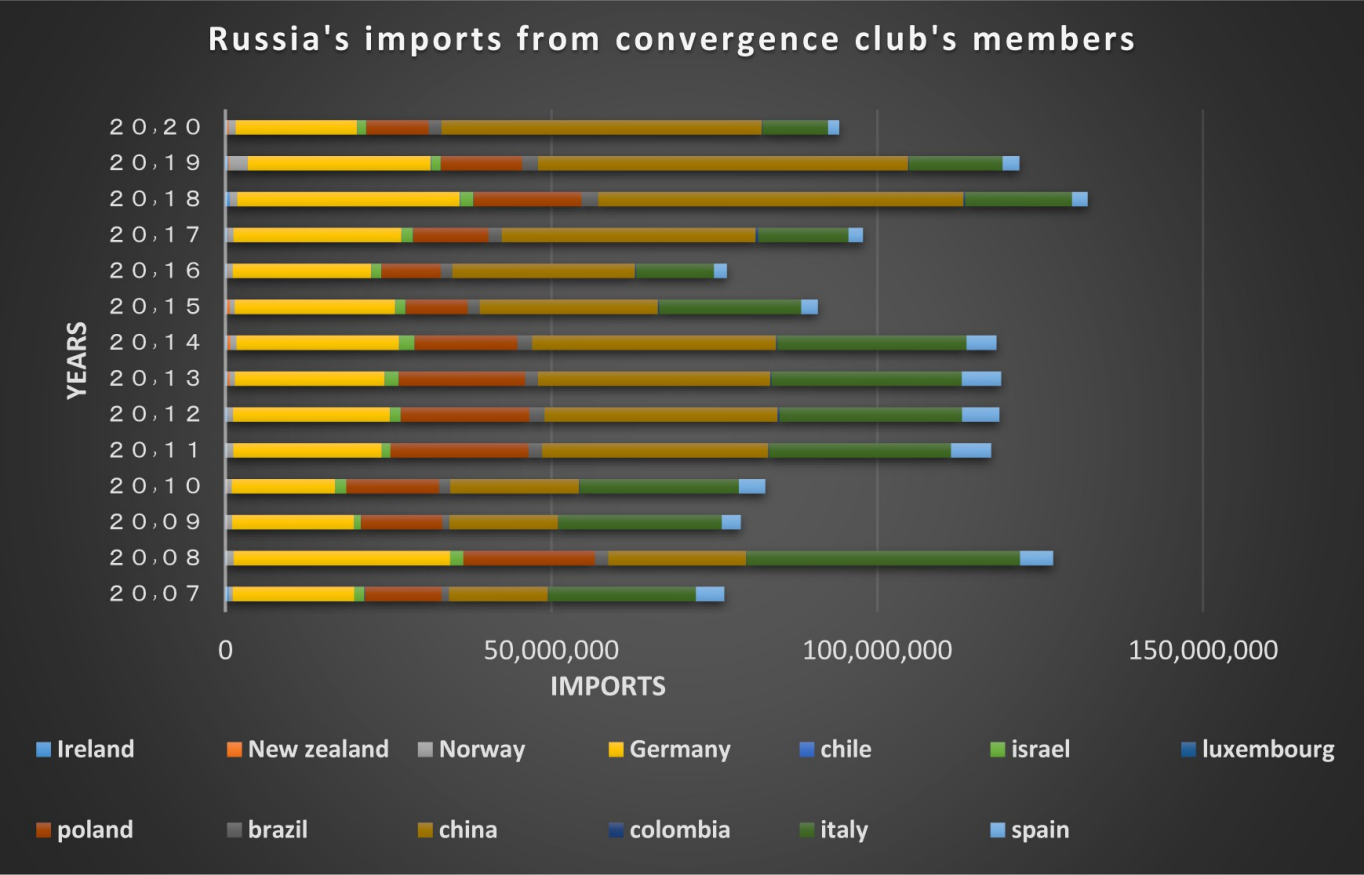
Russian Federation’s imports from other member countries of the convergence club in the capital market, from 2007–2020. Source: Trade Map.

**Fig 39:**
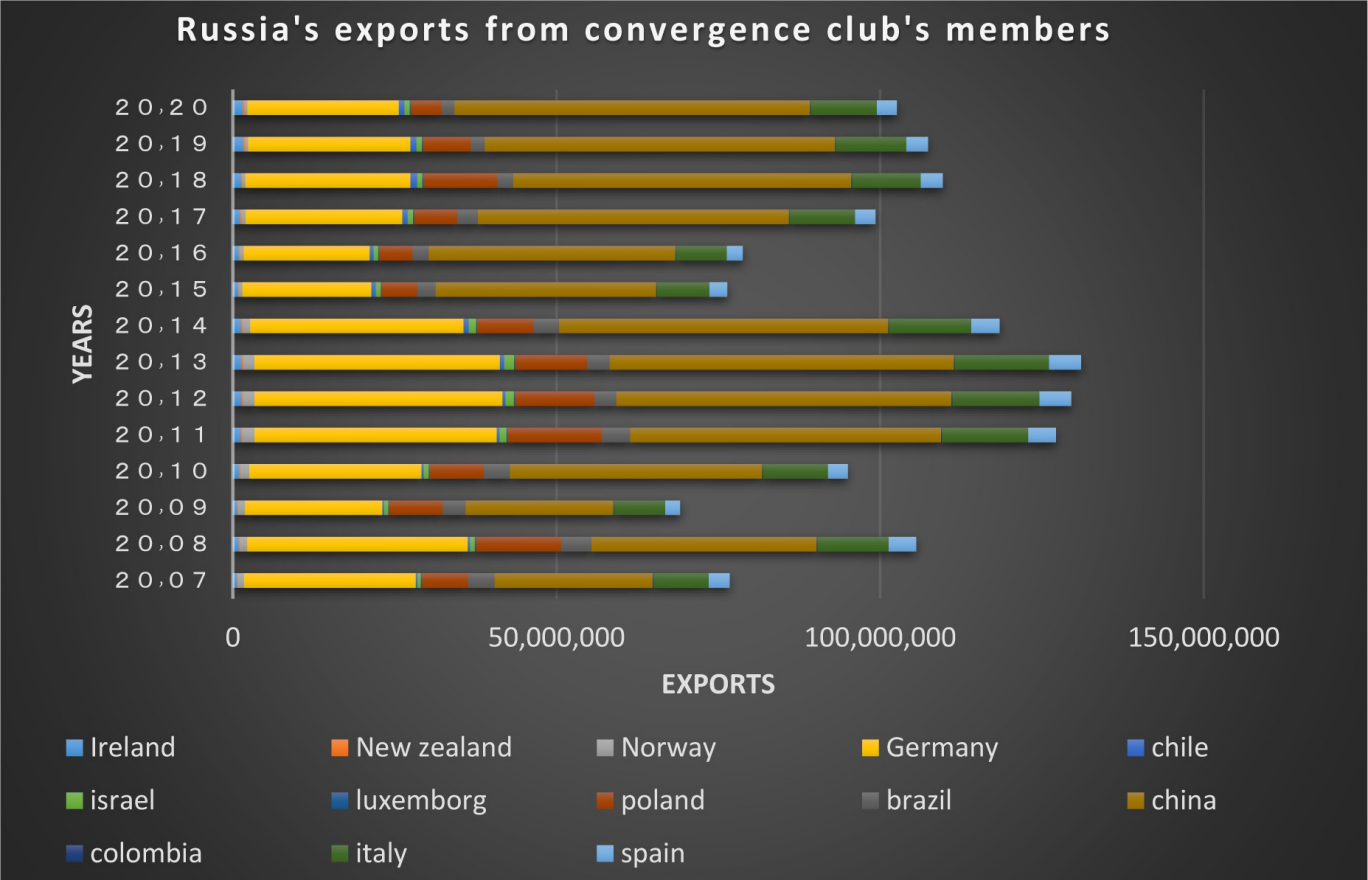
Russian Federation’s exports to other member countries of the convergence club in the capital market, from 2007–2020. Source: Trade Map.

**Fig 40:**
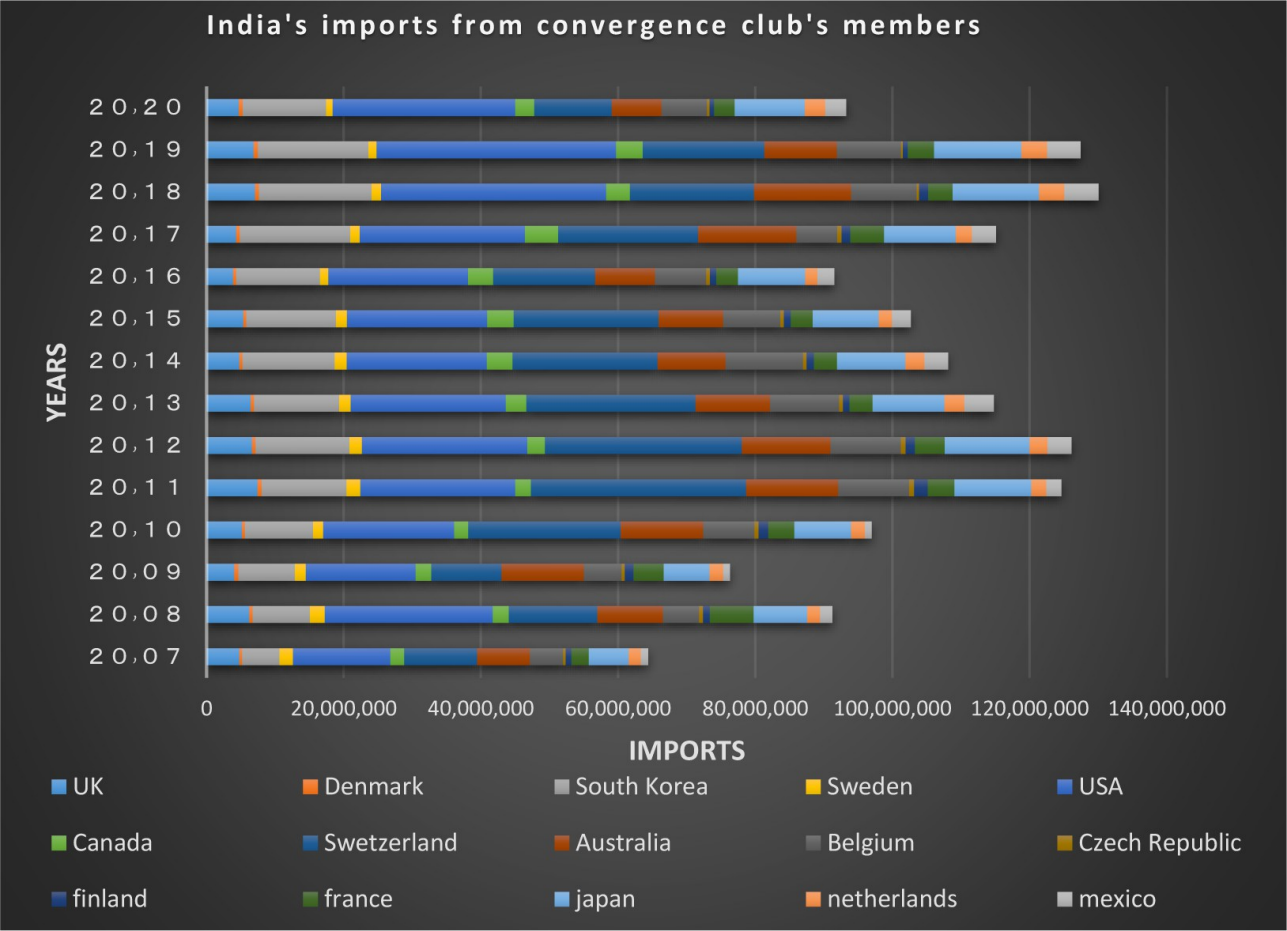
India’s imports from other countries in the convergence club in the capital market, from 2007–2020. Source: Trade Map.

**Fig 41:**
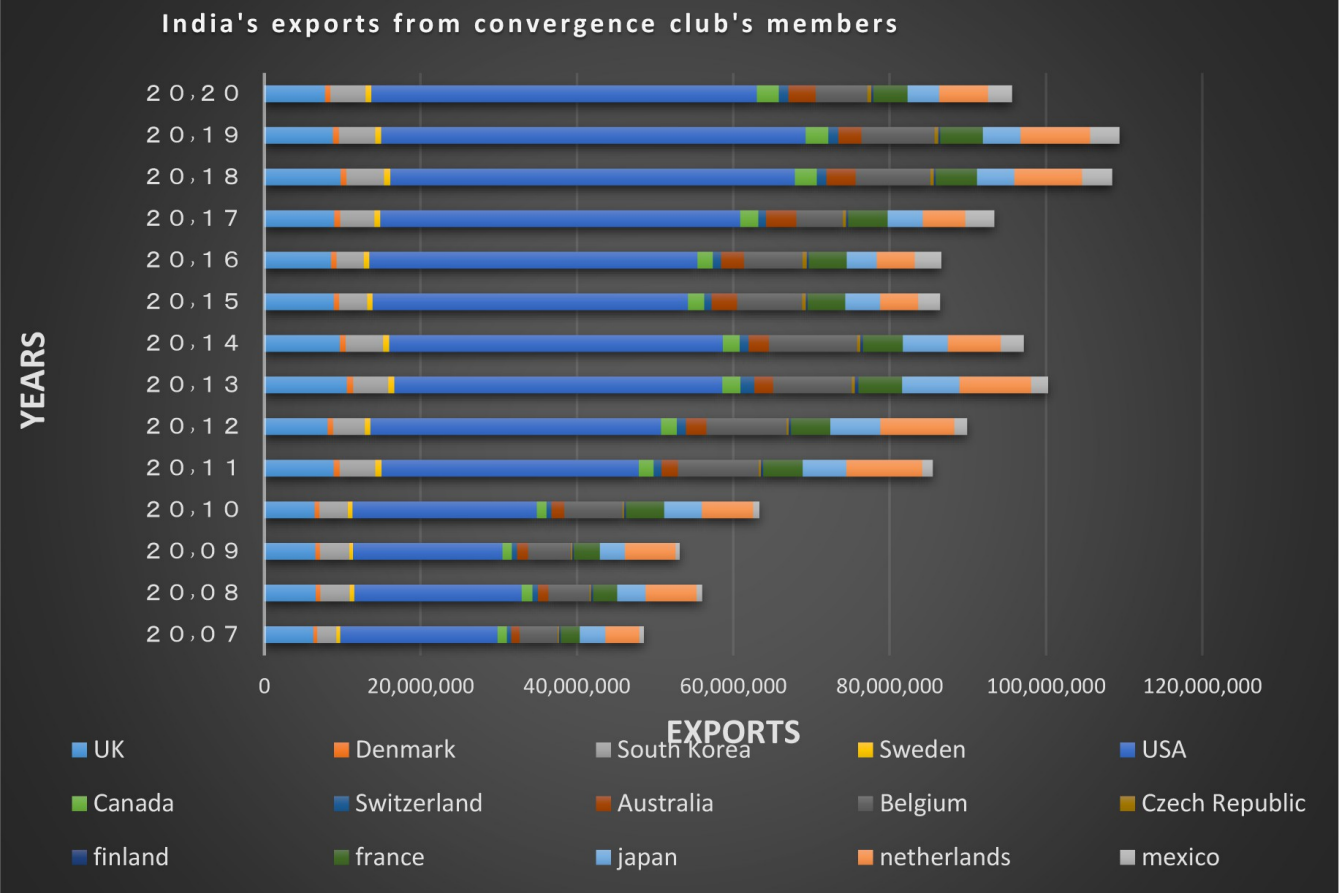
India’s exports to other countries in the convergence club in the capital market, from 2007–2020. Source: Trade Map.

According to Figs 34–41, the necessary condition for convergence, i.e. the existence of trade relations among converging countries in the capital market of each of the clubs, is established. In addition, the largest trading partners of each BRICS members including the United States, Japan, Australia, Canada, which are India’s main trading partners, are in a joint club with them. The European Union, Russia, Brazil, the main trading partners of China and Germany, Italy, Poland, Brazil, the main partners of Russia and Germany, Spain, Chile, Mexico, Italy, which are the main trading partners of Brazil, are also in a joint club with converging countries in the capital market.

First-club countries all have strong stock markets and the majority of them are of the top countries in terms of stock market value. The Indian stock market is also the third largest stock market in Asia, so its convergence with the world’s top stock markets is acceptable. One of the important factors of presence of a large number of countries in the first club is the presence of the United States in the club; in facts, as America has the world’s largest national economy and leading global trades, other stock markets tend to be in harmony with the US stock market.

**Fig 42:**
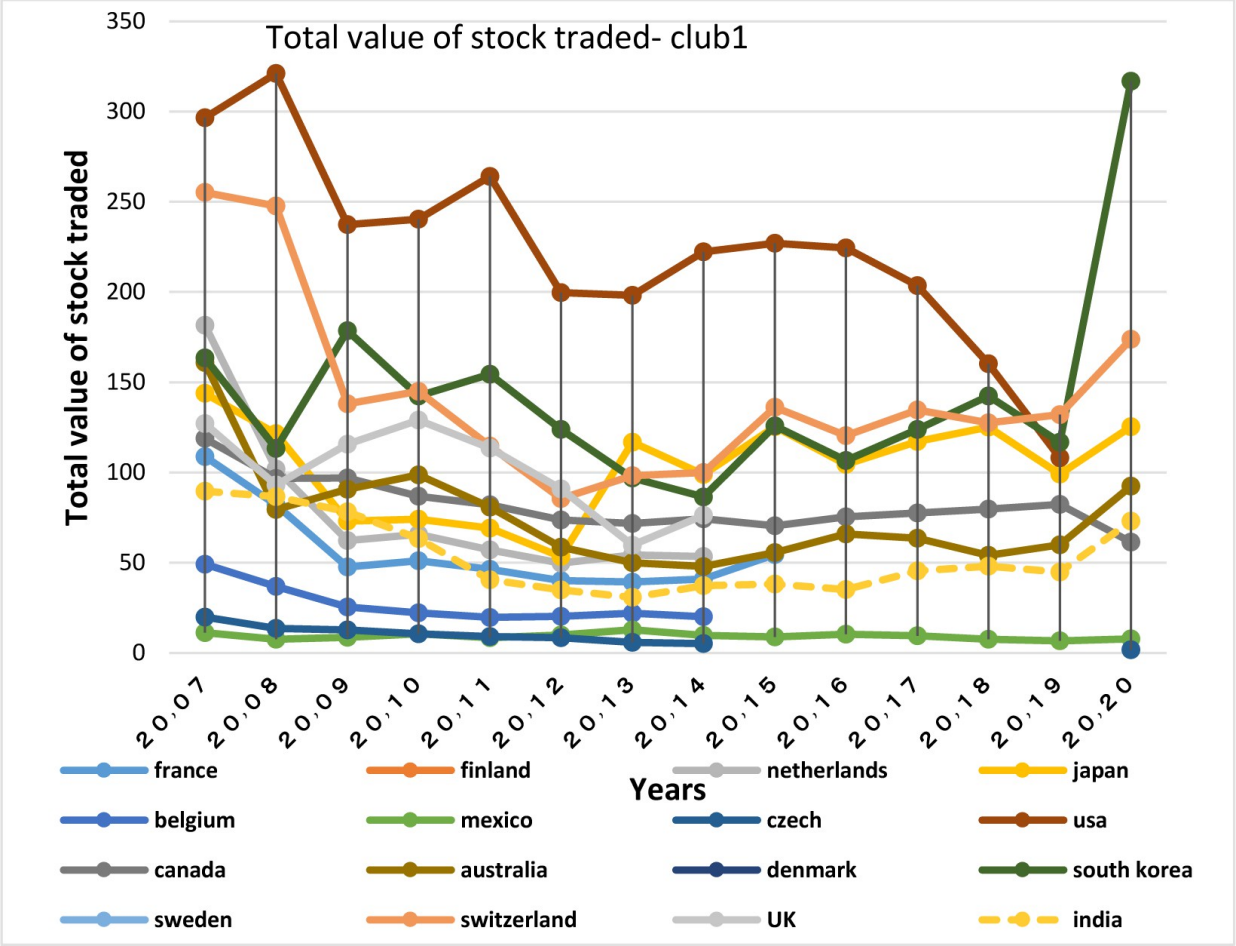
Total value of stock traded, convergence Club (1) in the capital market. From 2007 to 2020. The value of shares traded is the total number of shares traded, both domestic and foreign, multiplied by their respective matching prices. Sours: World Bank.

**Fig 43:**
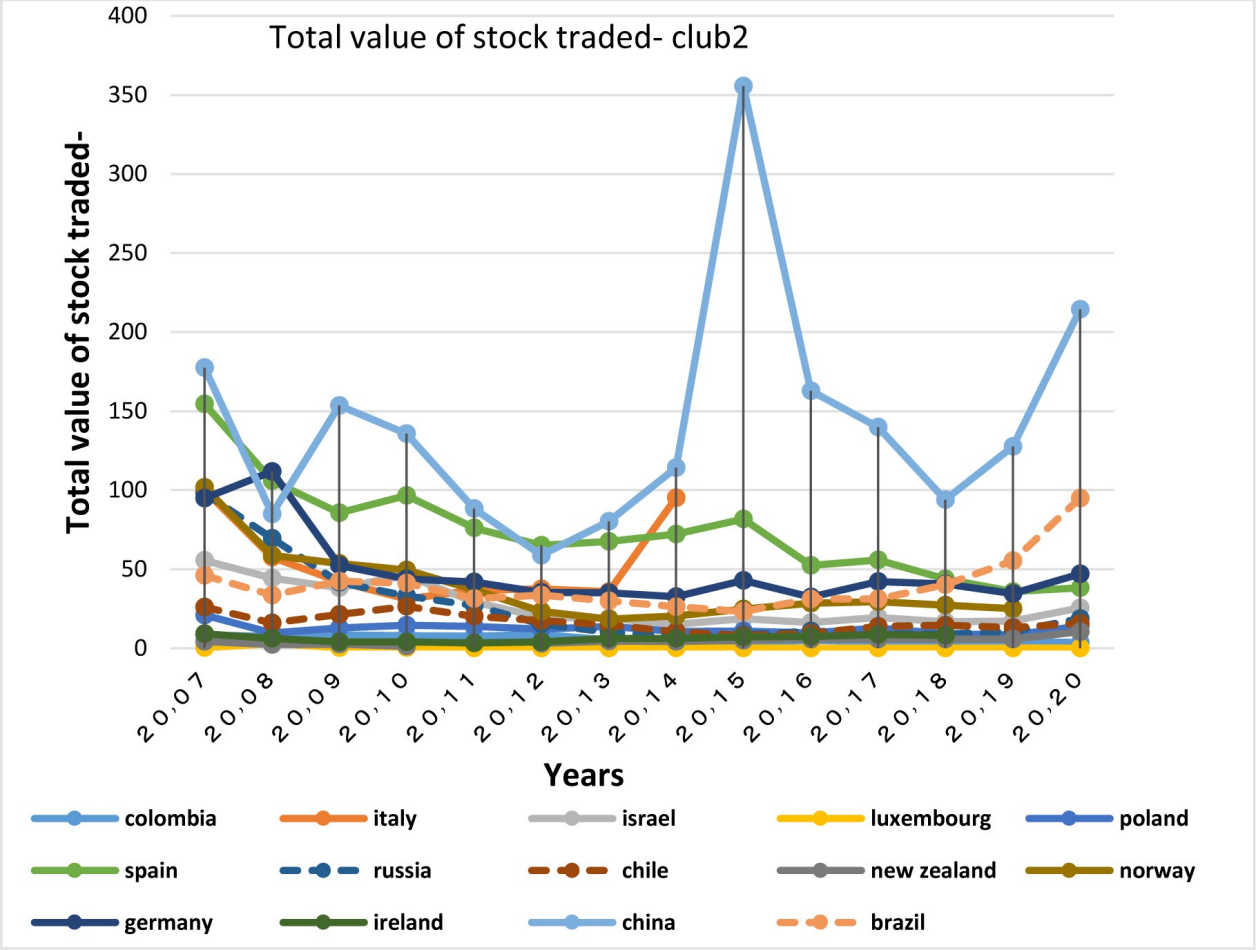
Total value of stock traded, convergence Club (2) in the capital market. From 2007 to 2020. Sours: World Bank.

**Fig 44:**
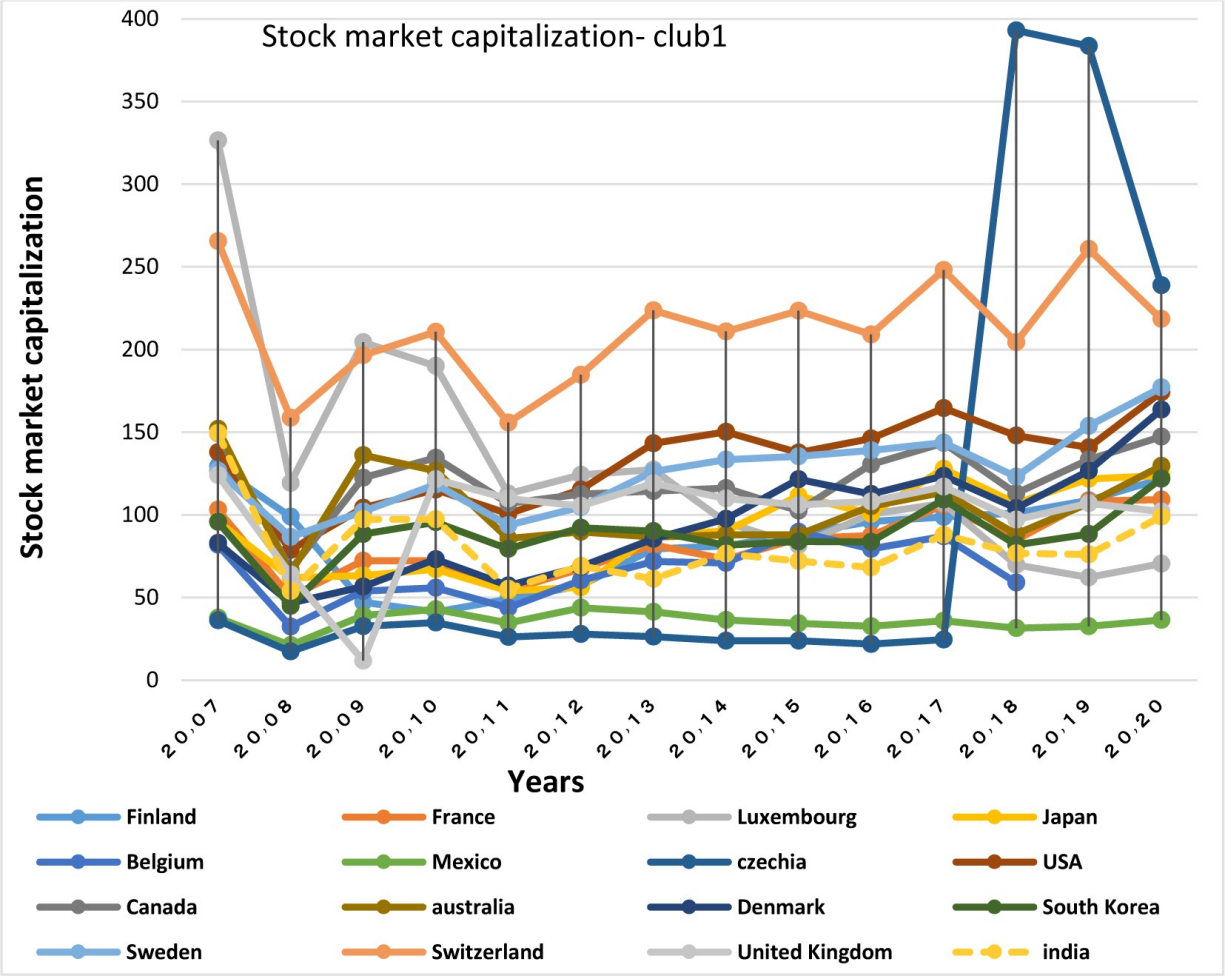
Stock market capitalization, convergence Club (1) in the capital market. From 2007 to 2020. Stock market capitalization (% of GDP) is the share price times the number of shares outstanding (including their several classes) for listed domestic companies. Investment funds, unit trusts, and companies whose only business goal is to hold shares of other listed companies are excluded. Sours: World Bank.

**Fig 45:**
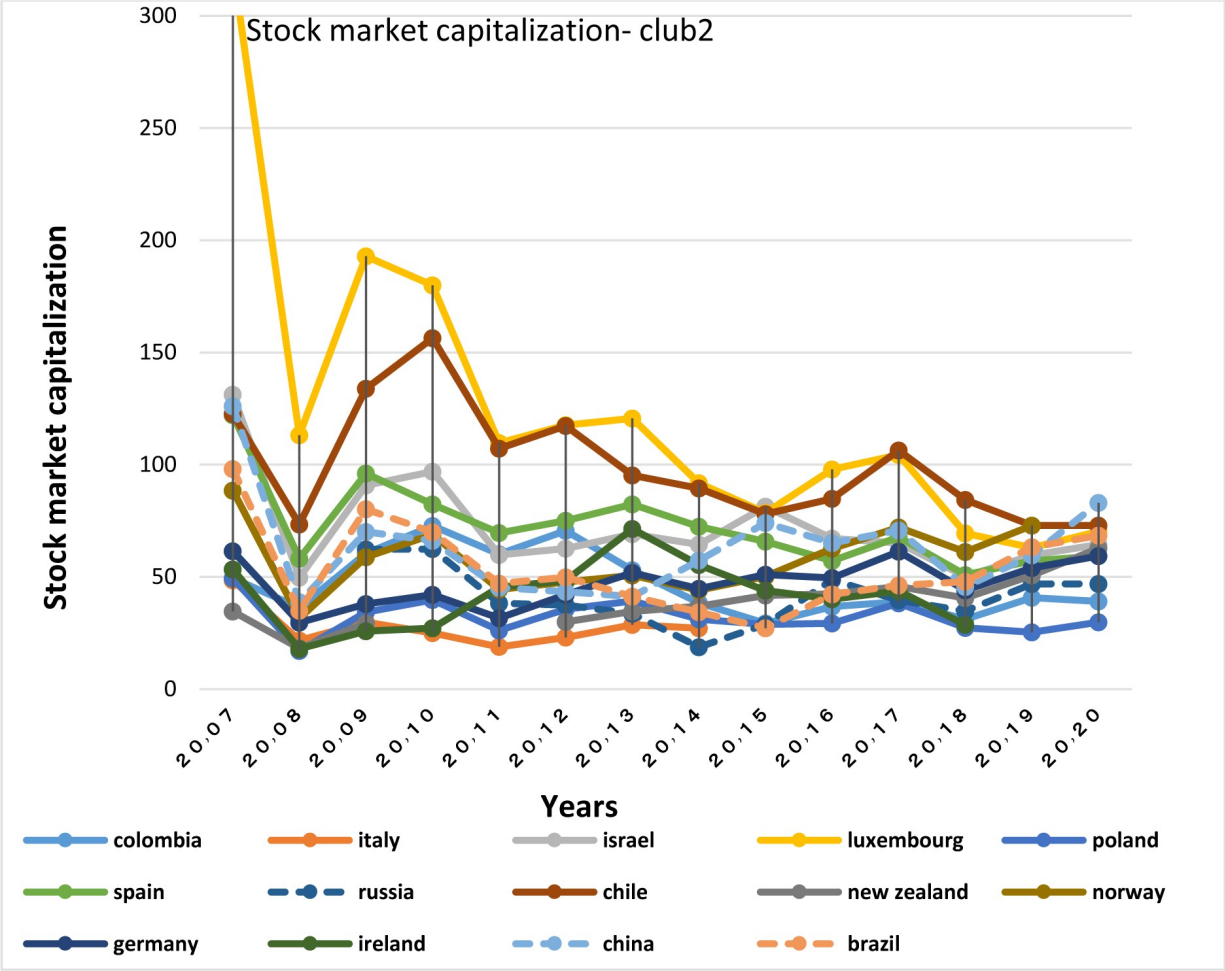
Stock market capitalization, convergence Club (2) in the capital market. From 2007 to 2020. Sours: World Bank.

The World Bank (2013) determines the total value of stock traded as the total value of shares traded during the period. Besides, it emphasizes that this index by showing whether market size corresponds to transactions or not, completes the market capitalization ratio. This is also used to measure the market depth in terms of liquidity or easiness of shares trading [69]. Therefore, to examine the stock market structure of the countries of the same club, Total value of stock traded (Figs 42 and 43) and stock market capitalization (Figs 44 and 45) diagrams are examined.

According to the diagrams it’s obvious that firstly the stock markets of the common countries in each club have followed a similar trend over a period of time. Secondly, in countries with stronger and more developed stock markets, the higher the market cap, the higher the value of their traded stock would be. Thirdly, the charts of Total value of stock traded, and stock market capitalization for the members of each club are similar and in agreement. In addition, the uptrend in the total value of stock traded charts during the study period of emerging BRICS countries in their clubs is considered; empathetically China which in addition to being above other groups in the mentioned index, has surpassed other countries in terms of market capitalization in 2020. It shows that in the ambiguous atmosphere of the Corona crisis, investors have seriously relied on the world’s second largest economy. Various factors have been involved in the growth of Chinese stocks. The factors include the rapid recovery of the China’s economy, the strength of the Yuan, and the increase in the number of initial public offerings of Chinese companies in recent years.

On the other hand, a comparison of economic growth (Figs 11, 12) and stock market capitalization to GDP [44, 45] shows that countries, by the increase in their level of income, tend to have a larger share of market capital.

In addition to what was mentioned above, foreign direct investment is another important factor that has a great effect on the stock market and the convergence of countries. The relationship between the stock market and FDI is quite undeniable. Three theoretical reasons confirm the relationship between these two indicators: firstly, FDI net flows strengthen the stock market by increasing the amount of funds to the host country’s economy. Secondly, the FDI flow prompt the host government to implement policies, rules, and controls that ultimately strengthen the stock market. Thirdly, the relationship between the stock market and foreign direct investment is a bidirectional relationship. The reason is that in case the stock markets are well developed and efficient enough, they would attract more FDI, due to the fact that multinational companies perceive security and low risk from such markets [70].

**Fig 46:**
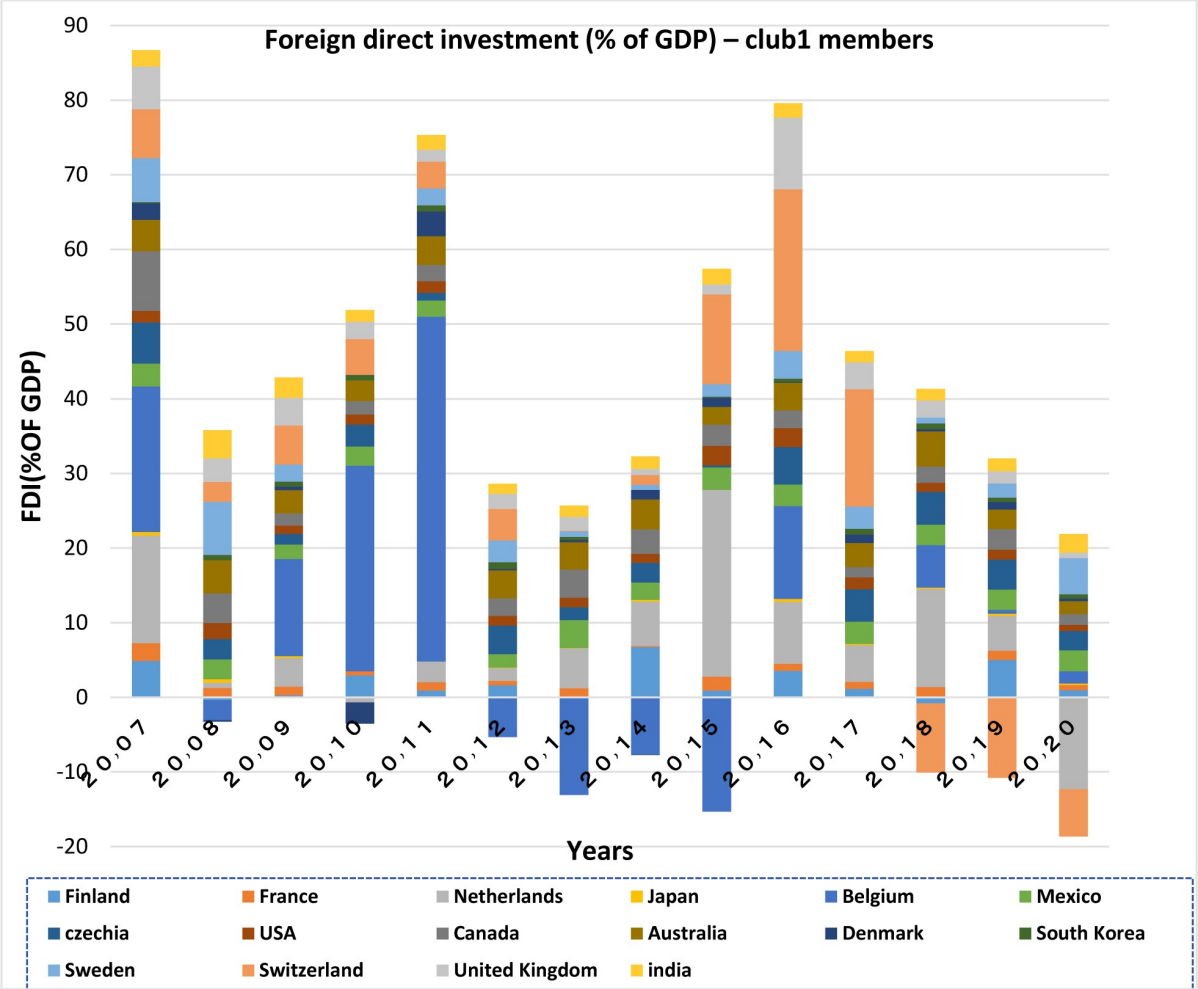
Foreign direct investment (% of GDP), convergence Club (1) in the capital market. From 2007 to 2020. Sours: World Bank.

**Fig 47:**
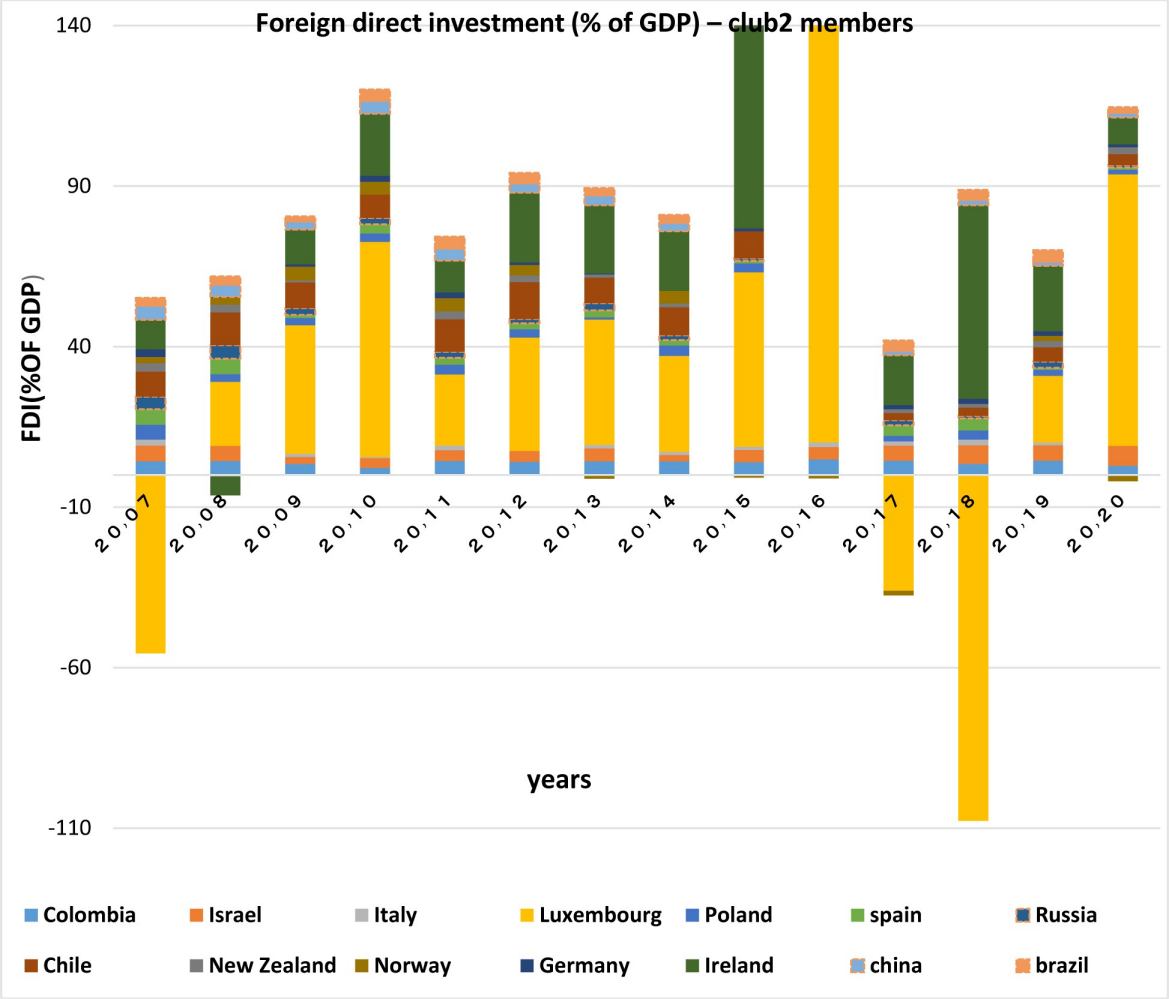
Foreign direct investment (% of GDP), convergence Club (2) in the capital market. From 2007 to 2020. Sours: World Bank.

Figs 46 and 47 show the foreign direct investment of countries within a joint club. By comparing FDI charts and stock market capitalization, it is evident that countries with a high market capitalization also receive significant FDI inflows. In moreover, the international flow of capital between countries reflects the development level of their financial markets and confirms their commercial and financial freedom. Emerging BRICS countries are key destinations for foreign investors, with foreign direct investment playing a crucial role in their influence in the global economy. Many foreign companies see BRICS countries as promising economies for investment opportunities. Therefore, as can be seen from the Figs; most of the OECD countries that have a convergence club with BRICS countries in the stock market, are the countries that have attracted the most foreign direct investment in their economies. Finally, the Figs demonstrate that FDI inflows into converging countries behave similarly, confirming their similar economic infrastructure.

### Summary of the discussion

In this section, we examined the necessary conditions for convergence between countries and found that trade relations have always been established between each of the BRICS countries and the OECD countries in their convergence club during the review period. All BRICS countries also enjoy trade freedom as another prerequisite. However, in terms of financial freedom, only Brazil and South Africa have reached the global average. Then, the economic structure of the converging countries was examined and the result showed that in recent years, the emerging BRICS countries have made great efforts to develop their economies. Now the similarity of their economic infrastructure with the developed OECD countries provides a sufficient condition for their convergence.

## 7. Conclusion and policy implications

In this paper, financial convergence between the OECD countries and BRICS countries has been examined. To do so, the annual statistical data of the period of 2007–2020 was used. Phillips and Sul’s method, the clustering algorithm, and a non-linear single-factor model with common and idiosyncratic components having an endogenous technological progress which differs across countries and over time was used.

This article, separating money and capital markets, is the first study work that has analyzed the convergence between BRICS and OECD financial markets. The idea of drawing the relative transfer path has also helped in understanding the subject comprehensively.

In this paper the conclusions drawn from the analysis have had significant theoretical and policy implications, shedding light on the dynamics of financial convergence between countries, particularly within the OECD and BRICS groups.

From a theoretical standpoint, the findings underscore the complexity of achieving financial convergence, as evidenced by the lack of general convergence observed between both OECD and BRICS countries, as well as within each group individually. This challenges the notion of a straightforward path towards financial integration and highlights the importance of considering various factors influencing convergence. The findings of [5, 31, 32, 71] also support these results.

In the course of this research, evaluating the clustering algorithm test in each financial market confirms the formation of convergence clubs among members of the BRICS and a considerable number of developed OECD countries [72]. Have reached similar results, while [73] have reached opposite results compared to the present study. After identifying the convergent clubs, it was determined that the BRICS countries have the necessary and sufficient conditions to integrate with OECD financial markets. Factors such as Financial and trade freedom, and the presence of commercial relations and agreements are required conditions for achieving financial convergence; similar trends in interest rates, inflation rates, money supply, and GDP growth provide sufficient conditions for convergence; however, the dispersion among the rates indicates relative convergence or the slow speed of convergence.

Analyzing sufficient and necessary conditions for financial market integration is a requirement of this topic, which had been given less attention before this survey. Understanding these items results in efficient policy adoptions in macroeconomics with the aim of convergence of financial markets at the international level. Future studies will develop knowledge in financial convergence by more precisely investigating this conditions and analyzing them for other emerging countries.

Also, by proposing the countries of each club as model countries for financial convergence the findings of this research help policy making.

On a policy level, the results suggest several key considerations for policymakers aiming to foster financial convergence. Firstly, the importance of bilateral relationships particularly in terms of trade treaties, cross-border investment and broader social, educational and health ties in driving convergence cannot be overstated. Strengthening these relationships might facilitate greater alignment in financial markets.

Moreover, as evidenced by the inclusion of most EU countries in convergence clubs the findings emphasize the role of institutional frameworks such as strong monetary unions, legal systems and Common borders in promoting convergence. Policymakers shall therefore prioritize measures to enhance institutional quality and stability including efforts to increase financial transparency, strengthen legal frameworks, and reduce political risk.

For emerging economies seeking deeper financial integration with developed countries, targeted policies are needed to address specific challenges including reducing investment risk, enhancing financial market stability, and promoting financial liberalization and market deepening. Measures such as capital account liberalization and diversification of financial institutions might help attract investment and facilitate convergence.

However, policymakers must also be mindful of the potential drawbacks associated with financial convergence, particularly for countries with weaker financial institutions. Financial liberalization might increase vulnerability to speculate attacks and exacerbate the spread of financial crises. Therefore, future researches shall aim to provide a more comprehensive understanding of both benefits and risks of convergence, guiding policymakers in formulating balanced and effective policy responses.

Last but not least, according to this survey literature, it is obvious that in previous studies, the financial convergence has been investigated using approximately equal empirical strategies. This continuation makes the researchers and policymakers hesitate in decision making only by increasing the number of contradictory results. So it is suggested that the future researchers provide more consistent and reliable results by concentrating on new techniques.

## Abbreviations

OECD: Organization for Economic Co-operation and Development
BRICS: is an acronym for five leading emerging economies: Brazil, Russia, India, China, and South Africa
PS: Philips and Sul
FDI: Foreign Direct Investment
OLS: Ordinary Least Squares method
FTA: Free Trade Agreements
S-CFTA: Switzerland-China Free Trade Agreement
Mercosur: Southern Common Market
M3: Money Supply
IMF: International Monetary Fund
